# Novel two-parameter quadratic exponential distribution: Properties, simulation, and applications

**DOI:** 10.1016/j.heliyon.2024.e38201

**Published:** 2024-09-24

**Authors:** Fatma Zohra Bousseba, Halim Zeghdoudi, Laxmi Prasad Sapkota, Yusra A. Tashkandy, M.E. Bakr, Anoop Kumar, Ahmed M. Gemeay

**Affiliations:** a LaPS Laboratory, Badji Mokhtar-Annaba University, Annaba 23000, Algeria; b Department of Statistics, Tribhuvan University, Tribhuvan Multiple Campus, Palpa, Nepal; c Department of Statistics and Operations Research, College of Science, King Saud University, P.O. Box 2455, Riyadh 11451, Saudi Arabia; d Department of Statistics, Faculty of Basic Science, Central University of Haryana, Mahendergarh, 123031, India; e Department of Mathematics, Faculty of Science, Tanta University, Tanta 31527, Egypt

## Abstract

This research paper introduces a novel two-parameter quadratic exponential distribution (NTPQED), thoroughly examining its statistical properties and practical applications. The study delves into essential characteristics of the distribution, including its asymptotic behavior, moments, order statistics, and entropies. Additionally, we present fuzzy reliability, value at risk, mean excess function, limited expected value function, tail value at risk, and tail variance. A comprehensive simulation study evaluates fifteen parameter estimation methods. To illustrate the practical relevance of the proposed distribution, three sets of real-world data are analyzed. The performance of the proposed distribution model is rigorously assessed using various model selection criteria and goodness-of-fit test statistics. Empirical findings consistently demonstrate the proposed distribution's superiority over existing models, highlighting its potential for multiple applications.

## 1 Introduction

Modeling data by generalized distributions is still widespread nowadays. Several researchers proposed new generalizations for lifetime distributions used in various fields such as finance, actuarial science, medicine, and engineering. For example, Zeghdoudi and Nedjar [19] derived the Gamma Lindley distribution to enhance the capability of analyzing various forms of lifetime data. Eghwerido et al. [9] define a three-parameter class for lifetime Poisson processes in the Marshall-Olkin transformation family. Gemeay et al. [11] introduced a novel general two-parameter statistical distribution, which can be presented as a mix of both exponential and gamma distributions. Bouhadjar et al. [14] proved that the power XLindley (PXL) distribution is significant for addressing fuzzy reliability modeling and modeling the data of the COVID-19 case. Belil et al. [5] defined the new flexible two-parameter family of distributions illustrated by the annual maximum flood and survival times of patients with breast cancer real data analyses. Additionally, Beghriche et al. [4] introduced a new polynomial exponential distribution with one parameter. The resulting model, known as polynomial exponential extended distribution, involves the original distribution as a special case and provides greater flexibility to model different real data types. Almetwally and Meraou [2] derived a sin extension of the exponential distribution. Muhammed and Almetwally [15] presented a bivariate version of inverse Weibull distribution.

Typically, the analysis utilizes distributions with a minimum of three parameters to adjust the higher-order moments accurately. Nevertheless, there are instances where the utilization of two-parameter probability distributions is favored, such as in the calculation of maximum flow, maximum precipitation, and maximum volumes. Additionally, they may be employed in applying probability distribution functions like the synthetic unit hydrograph.

The new approach introduces novel elements and connections for various distributions relevant to frequency analysis. This approach offers distinct benefits compared to the commonly used two-parameter distributions in hydrology, such as Extension of exponential, Burr type X (BurrX), Gumbel, new XLindley, and exponential. It provides greater flexibility by allowing for the modeling of various heavy-tailed curves, which is advantageous because these distributions exhibit different values for higher-order moments (coefficient of variation, skewness, kurtosis). The motivations of this proposed model can be summarized as follows:

• The NTPQED model has simple closed forms for the cumulative distribution function (CDF) and hazard rate function (HRF), making it suitable for analyzing censored data.
• The NTPQED model can be expressed as a mixture of the Lindley distribution and a specific case of the generalized gamma distribution.
• The NTPQED distribution exhibits various hazard rates, including increasing, decreasing, and bathtub-shaped, whereas the RL model only has an increasing hazard rate.
• The NTPQED distribution outperformed several well-known distributions, such as the Extension of exponential, new XLindley, Modified XLindley, Burr type X (BurrX), and exponential distributions. This was demonstrated using three real datasets from medicine, survival analysis, and geology.
  Additionally, this study aims to demonstrate how various frequentist estimators of the NTPQED parameters select the optimal parameter estimate method for the proposed model. This finding should be precious to practitioners and applied statisticians. Several writers have studied the estimate of parameters in generalized models using conventional estimation methods and have compared them based on numerical simulations.

The remaining sections of this article are managed as follows. The newly developed model is presented in Section 2 along with some survival-related functions. In Section 3, we briefly explained the mathematical and statistical properties of the suggested model. Several parameter estimation techniques are discussed in Section 4. Section 5, a numerical simulation of the parameter estimation is carried out. In section 6, the application of the suggested model is provided. Finally, some conclusive remarks are provided in Section 7.

## 2 Distribution model formulation

The distribution of *X* is a Noval Two-parameter quadratic exponential distribution (shortly NTPQED), and the probability density function (PDF) can be written as

$$f(x;\alpha ,\beta )=C\left(\alpha +\beta x+x^{2}\right)\exp(-\beta x),$$

where C=C(α,β) is real-valued functions on [0,+∞[. We can check immediately:

• It is non-negative for x>0.
• $P[a<x<b]=\int_{a}^{b}f(x;\alpha ,\beta )dx$.
• $\int_{0}^{\infty }f(x;\alpha ,\beta )dx=1$.

In this paper, we proposed a general version of NTPQED when

$$C(\alpha ,\beta )=\frac{\beta^{3}}{\beta^{2}+\alpha \beta^{2}+2}.$$

Then, its PDF is given by

(1) $$f(x;\alpha ,\beta )=\frac{\beta^{3}\left(\alpha +\beta x+x^{2}\right)}{\beta^{2}+\alpha \beta^{2}+2}\exp(-\beta x);\;x,\alpha ,\beta >0.$$

The corresponding cumulative distribution function (CDF), survival function (SF), and hazard rate function (HRF) are given by

(2) $$F(x;\alpha ,\beta )=1-e^{{}^{-\beta x}}\left(1+\frac{\beta^{2}x^{2}+\left(\beta^{3}+2\beta \right)x}{\beta^{2}+\alpha \beta^{2}+2}\right),$$

$$S(x;\alpha ,\beta )=e^{-\beta x}\left(1+\frac{\beta^{2}x^{2}+\left(\beta^{3}+2\beta \right)x}{\beta^{2}+\alpha \beta^{2}+2}\right),$$

(3) $$h(x;\alpha ,\beta )=\frac{\beta^{3}\left(\alpha +\beta x+x^{2}\right)}{\beta^{2}x^{2}+\left(\beta^{3}+2\beta \right)x+\beta^{2}+\alpha \beta^{2}+2}.$$

**Figure 1:**
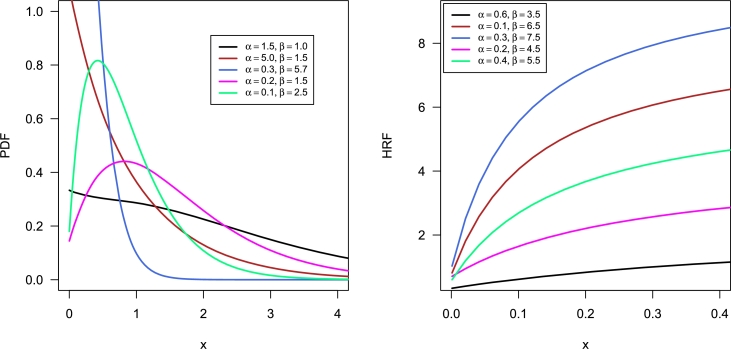
Some shapes of PDF and HRF of NTPQED.

In Fig. 1, we have displayed the various possible shapes of PDF and HRF of NTPQED. It is observed that PDF of NTPQED can have uni-model, right-skewed, decreasing, and reverse J-shaped, while HRF can have constant or increasing curves.

## 3 A general theoretical results

### 3.1 Asymptotic behavior

This subsection discusses the shape characteristics of the PDF NTPQED and HRF NTPQED in Equations (1) and (3), respectively, of the NTPQED. The behavior of NTPQED at x=0 and x=∞, respectively, are given by

$$\lim_{y\to 0}f(x;\alpha ,\beta )=\frac{\alpha \beta^{3}}{\beta^{2}+\alpha \beta^{2}+2},\lim_{y\to \infty }f(x;\alpha ,\beta )=0.$$

The behavior of h(x;α,β) at x=0 and x=∞, respectively, are given by

$$\lim_{y\to 0}h(x;\alpha ,\beta )=\frac{\alpha \beta^{3}}{\beta^{2}+\alpha \beta^{2}+2},\lim_{y\to \infty }h(x;\alpha ,\beta )=\beta .$$

The following proposition states that there are two shapes for the PDF of the two-parameter polynomial exponential distribution, depending on the range of the parameters *α* and *β*.

Proposition 1 *The PDF f* (x;α,β) *in Equation* (1) *of the NTPQED is*

*1.* *Decreasing if* $\sqrt{\beta^{4}-4\beta^{2}\alpha +4}-\beta^{2}+2<0$ *.*
*2.* *Unimodel if* $\sqrt{\beta^{4}-4\beta^{2}\alpha +4}-\beta^{2}+2>0$ *.*

Proof The first and the second derivative of the PDF in (1) is determined as follows

$$\frac{df(x;\alpha ,\beta )}{dx}=-be^{-x\beta }\left(\beta \alpha -\beta +\left(\beta^{2}-2\right)x+\beta x^{2}\right),$$

$$\frac{d^{2}f(x;\alpha ,\beta )}{dx^{2}}=be^{-x\beta }\left(\beta^{2}x^{2}+\left(\beta^{3}-4\beta \right)x+\alpha \beta^{2}-2\beta^{2}+2\right),$$

by equating the last equation to zero and solving it concerning *y*, we have its solutions as follows

$$x_{1}=-\frac{1}{2\beta }\left(\sqrt{\beta^{4}-4\beta^{2}\alpha +4}+\beta^{2}-2\right)<0,$$

$$x_{2}=\frac{1}{2\beta }\left(\sqrt{\beta^{4}-4\beta^{2}\alpha +4}-\beta^{2}+2\right)$$

then, our critical point is $\overset{ˆ}{x}=\frac{1}{2\beta }\left(\sqrt{\beta^{4}-4\beta^{2}\alpha +4}-\beta^{2}+2\right)$, if $\sqrt{\beta^{4}-4\beta^{2}\alpha +4}-\beta^{2}+2>0$.

$$\frac{d^{2}f(x;\alpha ,\beta )}{dx^{2}}=-\sqrt{\beta^{4}-4\alpha \beta^{2}+4}<0,$$

then, ∀α,β>0, $\overset{ˆ}{x}=\frac{1}{2\beta }\left(\sqrt{\beta^{4}-4\beta^{2}\alpha +4}-\beta^{2}+2\right)$ is the unique critical point which maximize the PDF (1) and it is unimodal function.

Therefore, the mode of NTPQED is defined as follows

$$Mod=\frac{1}{2\beta }\left(\sqrt{\beta^{4}-4\beta^{2}\alpha +4}-\beta^{2}+2\right).$$

If $\sqrt{\beta^{4}-4\beta^{2}\alpha +4}-\beta^{2}+2<0$, the PDF is decreasing, the mode will be

$$Mod=\frac{\alpha \beta^{3}}{\beta^{2}+\alpha \beta^{2}+2}.$$

 

Proposition 2 *Let* h(x;α,β) *be the hazard rate function of the NTPQED. Then* h(x;α,β) *is*

*1.* *Increasing if* $-\beta^{4}+4\beta^{2}\alpha +4<0$ *.*
*2.* *Decreasing if* $\beta^{2}+2-\sqrt{-\beta^{4}+4\beta^{2}\alpha +4}>0$ *and* $-\beta^{4}+4\beta^{2}\alpha +4>0$ *.*
*3.* *bathtub-shaped if* $\beta^{2}+2-\sqrt{-\beta^{4}+4\alpha \beta^{2}+4}<0$ *and* $-\beta^{4}+4\beta^{2}\alpha +4>0$ *.*

Proof The first derivative of h(x;α,β) is

$$\frac{dh(x;\alpha ,\beta )}{dx}=\frac{\beta^{3}L\left(y\right)\left(2\beta x^{2}+\left(2\beta^{2}+4\right)x+\beta^{3}+2\beta -2\beta \alpha \right)}{{\left(\beta^{2}x^{2}+\beta^{3}x+\alpha \beta^{2}+2\beta x+\beta^{2}+2\right)}^{2}},$$

where $L\left(x\right)=2cx^{2}+\left(2\beta^{2}+4\right)x+\beta^{3}+2\beta -2\beta \alpha$.

We can see that $\frac{dh(x;\alpha ,\beta )}{dx}$ and L(x) have the same sign. Using the characteristics of the quadratic function L(x), the function $L\left(x^{\alpha }\right)$ is negative for $-\beta^{4}+4\beta^{2}\alpha +4<0$, positive for $\beta^{2}+2-\sqrt{-\beta^{4}+4c^{2}\alpha +4}>0$ and $-\beta^{4}+4\beta^{2}\alpha +4>0$ and changes sign from negative to positive for $\beta^{2}+2-\sqrt{-\beta^{4}+4\beta^{2}\alpha +4}<0$ and $-\beta^{4}+4\beta^{2}\alpha +4>0$. 

### 3.2 Moments and related measures of NTPQED

Let *X*∼ NTPQED, then the *r*th moment of *X* is determined as follows

(4) $$E(X^{r})=\int_{0}^{\infty }x^{r}f(x,\alpha ,\beta )dx=\int_{0}^{\infty }x^{r}\frac{\beta^{3}\left(\alpha +\beta x+x^{2}\right)}{\beta^{2}+\alpha \beta^{2}+2}\exp(-\beta x)dx,=\frac{1}{\beta^{r}}\frac{\Gamma \left(r+3\right)+\beta^{2}\Gamma \left(r+2\right)+\alpha \beta^{2}\Gamma \left(r+1\right)}{\beta^{2}+\alpha \beta^{2}+2}.$$

Hence, the first four moments of the NTPQED random variable can be found by substituting i=1,2,3,4, respectively, in Equation (4). They are used to determine mean, variance, skewness, kurtosis, and coefficient of variation of NTPQED, respectively, as follows

$$E(X)=\frac{\left(2\beta^{2}+\alpha \beta^{2}+6\right)}{\beta \left(\beta^{2}+\alpha \beta^{2}+2\right)}.$$

$$Var(X)=E(X^{2})-E(X)=\frac{\left(6\beta^{2}-6\beta -2\beta^{3}+2\alpha \beta^{2}-\alpha \beta^{3}+24\right)}{\beta^{2}\left(\beta^{2}+\alpha \beta^{2}+2\right)}\;\frac{1}{\beta^{2}}\frac{6\beta^{2}+2\alpha \beta^{2}+24}{\beta^{2}+\alpha \beta^{2}+2}-\frac{1}{\beta }\frac{2\beta^{2}+\alpha \beta^{2}+6}{\beta^{2}+\alpha \beta^{2}+2}.$$

$$Skewness=\sqrt{\beta_{1}}=\frac{E(X^{3})}{{\left(Var(X)\right)}^{\frac{3}{2}}}=\frac{\frac{\left(24\beta^{2}+6\alpha \beta^{2}+120\right)}{\beta^{3}\left(\beta^{2}+\alpha \beta^{2}+2\right)}}{{\left(\frac{\left(6\beta^{2}-6\beta -2\beta^{3}+2\alpha \beta^{2}-\alpha \beta^{3}+24\right)}{\beta^{2}\left(\beta^{2}+\alpha \beta^{2}+2\right)}\right)}^{\frac{3}{2}}}.$$

$$Kurtosis=\beta_{2}=\frac{E(X^{4})}{{\left(Var(X)\right)}^{2}}=\frac{\frac{\left(120\beta^{2}+24\alpha \beta^{2}+720\right)}{\beta^{4}\left(\beta^{2}+\alpha \beta^{2}+2\right)}}{{\left(\frac{\left(6\beta^{2}-6\beta -2\beta^{3}+2\alpha \beta^{2}-\alpha \beta^{3}+24\right)}{\beta^{2}\left(\beta^{2}+\alpha \beta^{2}+2\right)}\right)}^{2}}.$$

$$C.V=\gamma =\frac{\sqrt{Var(X)}}{E(X)}=\frac{\sqrt{\frac{\left(6\beta^{2}-6\beta -2\beta^{3}+2\alpha \beta^{2}-\alpha \beta^{3}+24\right)}{\beta^{2}\left(\beta^{2}+\alpha \beta^{2}+2\right)}}}{\frac{\left(2\beta^{2}+\alpha \beta^{2}+6\right)}{\beta \left(\beta^{2}+\alpha \beta^{2}+2\right)}}.$$

Theorem 1 *Let X follow the NTPQED, then* E(X)<Mod *.*

Proof We have $\mu =E(X)=\frac{\left(2\beta^{2}+\alpha \beta^{2}+6\right)}{\beta \left(\beta^{2}+\alpha \beta^{2}+2\right)}$ and $Mod=\frac{\alpha \beta^{3}}{\beta^{2}+\alpha \beta^{2}+2}$.

From the CDF defined in Equation (2), we have

$$F(\mu )=1-(\frac{\left(\alpha^{3}\beta^{6}+4\alpha^{2}\beta^{6}+9\alpha^{2}\beta^{4}+6\alpha \beta^{6}+30\alpha \beta^{4}+40\alpha \beta^{2}+3\beta^{6}+24\beta^{4}+68\beta^{2}+68\right)}{{\left(\beta^{2}+\alpha \beta^{2}+2\right)}^{3}})e^{-\frac{\left(2\beta^{2}+\alpha \beta^{2}+6\right)}{\left(\beta^{2}+\alpha \beta^{2}+2\right)}},$$

$$F(Mod)=1-\left(\frac{\left(\alpha^{3}\beta^{6}+2\alpha^{2}\beta^{8}+5\alpha^{2}\beta^{6}+6\alpha^{2}\beta^{4}+\alpha \beta^{8}+7\alpha \beta^{6}+16\alpha \beta^{4}+12\alpha \beta^{2}+\beta^{6}+6\beta^{4}+12\beta^{2}+8\right)}{{\left(\beta^{2}+\alpha \beta^{2}+2\right)}^{3}}\right)e^{{}^{-\beta \frac{\alpha \beta^{3}}{\left(\beta^{2}+\alpha \beta^{2}+2\right)}}},$$

where

$$P_{mod}\left(\alpha ,\beta \right)=\frac{\left(\alpha^{3}\beta^{6}+2\alpha^{2}\beta^{8}+5\alpha^{2}\beta^{6}+6\alpha^{2}\beta^{4}+\alpha \beta^{8}+7\alpha \beta^{6}+16\alpha \beta^{4}+12\alpha \beta^{2}+\beta^{6}+6\beta^{4}+12\beta^{2}+8\right)}{{\left(\beta^{2}+\alpha \beta^{2}+2\right)}^{3}},P_{\mu }\left(\alpha ,\beta \right)=\frac{\left(\alpha^{3}\beta^{6}+4\alpha^{2}\beta^{6}+9\alpha^{2}\beta^{4}+6\alpha \beta^{6}+30\alpha \beta^{4}+40\alpha \beta^{2}+3\beta^{6}+24\beta^{4}+68\beta^{2}+68\right)}{{\left(\beta^{2}+\alpha \beta^{2}+2\right)}^{3}}.$$

Now, we can check that

$$e^{-\frac{\left(2\beta^{2}+\alpha \beta^{2}+6\right)}{\left(\beta^{2}+\alpha \beta^{2}+2\right)}}>e^{{}^{-\beta \frac{\alpha \beta^{3}}{\left(\beta^{2}+\alpha \beta^{2}+2\right)}}}\text{ and }P_{\mu }\left(\alpha ,\beta \right)>P_{mod}\left(\alpha ,\beta \right).$$

Then

$$1-P_{\mu }e^{{}^{-\beta \frac{\alpha \beta^{3}}{\left(\beta^{2}+\alpha \beta^{2}+2\right)}}}<1-P_{Mod}e^{-\frac{\left(2\beta^{2}+\alpha \beta^{2}+6\right)}{\left(\beta^{2}+\alpha \beta^{2}+2\right)}},$$

thus,

F(μ;α,β)<F(Mod;α,β).

Finally, since F(x;α,β) is an increasing function in x, for all α,β>0, we have μ<Mod. 

The moment-generating function of the NTPQED is determined as follows

$$M\left(s\right)=\int e^{sx}f(x,\alpha ,\beta )dx=\int e^{sx}\;\frac{\beta^{3}\left(\alpha +\beta x+x^{2}\right)}{\beta^{2}+\alpha \beta^{2}+2}\exp(-\beta x)dx=\beta^{3}\frac{e^{sx-x\beta }}{{\left(s-\beta \right)}^{3}\left(\beta^{2}+\alpha \beta^{2}+2\right)}\times \left(x^{2}\beta^{2}-s\beta +2x\beta +\beta^{2}+s^{2}\alpha +x\beta^{3}+\alpha \beta^{2}+s^{2}x^{2}-2sx-2s\alpha \beta -2sx\beta^{2}-2sx^{2}\beta +s^{2}x\beta +2\right),\;s<\beta ,$$

its characteristic function is obtained by replacing t with it in the last equation.

The ith incomplete moments of NTPQED is determined as follows

(5) $$T_{r}\left(s\right)=\int_{0}^{s}t^{r}f(x,\alpha ,\beta )dx=\int_{0}^{s}t^{r}\frac{\beta^{3}\left(\alpha +\beta x+x^{2}\right)}{\beta^{2}+\alpha \beta^{2}+2}\exp(-\beta x)dx=\frac{\left(\Gamma \left(r+3\right)-\Gamma \left(r+3,s\beta \right)-\beta^{2}\Gamma \left(r+2,s\beta \right)+\beta^{2}\Gamma \left(r+2\right)-\alpha \beta^{2}\Gamma \left(r+1,s\beta \right)+\alpha \beta^{2}\Gamma \left(r+1\right)\right)}{\beta^{r}\left(\beta^{2}+\alpha \beta^{2}+2\right)},$$

where $\Gamma \left(\alpha ,x\right)=\int_{x}^{\infty }t^{\alpha -1}e^{-t}dx$. We have first incomplete moments $T_{1}\left(s\right)$ in Equation (5) when r=1 which is used to calculate the mean residual life and the mean waiting time, which are, respectively, defined as follows

$$\Psi \left(s\right)=\frac{1-T_{1}\left(s\right)}{S(x;\alpha ,\beta )-1}=\frac{\beta \left(\beta^{2}+\alpha \beta^{2}+2\right)-\left(\Gamma \left(4\right)-\Gamma \left(4,s\beta \right)-\beta^{2}\Gamma \left(3,s\beta \right)+\beta^{2}\Gamma \left(3\right)-\alpha \beta^{2}\Gamma \left(2,s\beta \right)+\alpha \beta^{2}\Gamma \left(2\right)\right)}{\beta \left((\beta^{2}+\alpha \beta^{2}+2+\beta^{2}x^{2}+(\beta^{3}+2\beta )x)e^{-\beta x}-\left(\beta^{2}+\alpha \beta^{2}+2\right)\right)}.$$

$$M_{1}\left(s\right)=\frac{1-T_{1}\left(s\right)}{F(x;\alpha ,\beta )}=\frac{\beta \left(\beta^{2}+\alpha \beta^{2}+2\right)-\left(\Gamma \left(4\right)-\Gamma \left(4,s\beta \right)-\beta^{2}\Gamma \left(3,s\beta \right)+\beta^{2}\Gamma \left(3\right)-\alpha \beta^{2}\Gamma \left(2,s\beta \right)+\alpha \beta^{2}\Gamma \left(2\right)\right)}{\beta \left(\left(\beta^{2}+\alpha \beta^{2}+2\right)-e^{{}^{-\beta x}}\left(\beta^{2}+\alpha \beta^{2}+2+\beta^{2}x^{2}+\left(\beta^{3}+2\beta \right)x\right)\right)}.$$

Another use of $T_{1}\left(s\right)$ is to calculate Bonferroni and Lorenz curves, which are, respectively, defined as follows

$$L(p)=\frac{T_{1}(t)}{E(x)}=\frac{-1}{2\beta^{2}+\alpha \beta^{2}+6}(6e^{-t\beta }-2\beta^{2}+2\beta^{2}e^{-t\beta }-\alpha \beta^{2}+2t\beta^{3}e^{-t\beta }+\alpha \beta^{2}e^{-t\beta }+3t^{2}\beta^{2}e^{-t\beta }+t^{2}\beta^{4}e^{-t\beta }+t^{3}\beta^{3}e^{-t\beta }+6t\beta e^{-t\beta }+t\alpha \beta^{3}e^{-t\beta }-6),B(p)=\frac{T_{1}(x_{p})}{\left(pE(x)\right)},$$

where $x_{p}$ is the quantile function of NTPQED.

### 3.3 Stochastic orders

Stochastic Order is an order of -largeness -on random variables. More broadly, stochastic orders are orders used to compare random variable's probability distributions or measurements.

Now, we consider two random variables, $Z_{1}$ and $Z_{2}$. Then $Z_{1}$ is said smaller than $Z_{2}$ in the following

• Likelihood ratio order ($Z_{1}{<}_{lr}Z_{2})$, if $\frac{f_{Z_{1}}(t)}{f_{Z_{2}}(t)}$ is decreasing in *t*.
• Hazard rate order ($Z_{1}{<}_{hr}Z_{2}$), if $h_{Z_{1}}(t)<h_{Z_{2}}(t)$,∀*t*.
• Convex order ($Z_{1}\leq_{cx}Z_{2})$, if for all convex functions *φ*, $E[\varphi (Z_{1})]\leq E[\varphi (Z_{2})]$ (expectation exist).

Remark 1 Likelihood ratio order ⟹ hazard rate order ⟹ Stochastic order. If $E[Z_{1}]=E[Z_{2}]$, then convex order ⟹Stochastic order.

Theorem 2 *Let* $Z_{1},Z_{2}∼TPQEF$ *be two random variables. If* $\alpha_{1}\geq \alpha_{2},\beta_{1}\geq \beta_{2}$ *and* $\alpha_{1}\beta_{2}\geq \alpha_{2},\beta_{1}$ *then:* $Z_{1}{<}_{lr}Z_{2}$ *;* $Z_{1}{<}_{hr}Z_{2}$ *;* $Z_{1}{<}_{S}Z_{2}$ *and* $Z_{1}\leq_{cx}Z_{2}$ *.*

Proof $$\frac{f_{Z_{1}}(t)}{f_{Z_{2}}(t)}=\frac{\beta_{1}^{3}\frac{t^{2}+\beta_{1}t+\alpha_{1}}{\beta_{1}^{2}+\alpha_{1}\beta_{1}^{2}+2}}{\beta_{2}^{3}\frac{t^{2}+\beta_{2}t+\alpha_{2}}{\beta_{2}^{2}+\alpha_{2}\beta_{2}^{2}+2}}e^{-(\beta_{1}-\beta_{2})t}.$$

To keep it simple, we use $\ln\frac{f_{Z_{1}}(t)}{f_{Z_{2}}(t)}$, which we find after derivation:

$$\frac{d}{dt}\ln\left(\frac{f_{Z_{1}}(t)}{f_{Z_{2}}(t)}\right)=\frac{2t+\beta_{1}}{t^{2}+\beta_{1}t+\alpha_{1}}-\frac{2t+\beta_{2}}{t^{2}+\beta_{2}t+\alpha_{2}}-(\beta_{1}-\beta_{2}).$$

We can check that $\frac{2t+\beta_{1}}{t^{2}+\beta_{1}t+\alpha_{1}}\leq \frac{2t+\beta_{2}}{t^{2}+\beta_{2}t+\alpha_{2}}$, if $\alpha_{1}\geq \alpha_{2},\beta_{1}\geq \beta_{2}$ and $\alpha_{1}\beta_{2}\geq \alpha_{2},\beta_{1}$. Since, $\frac{d}{dt}\ln\left(\frac{f_{Z_{1}}(t)}{f_{Z_{2}}(t)}\right)\leq 0$. 

### 3.4 Entropies

It is commonly understood that entropy and information can be used to calculate the degree of uncertainty in a probability distribution. However, many correlations have been created based on the features of entropy. The entropy of a random variable *X* measures the uncertainty's variation. The entropy of Rényi is defined as:

$$I_{R}\left(s\right)=\frac{1}{1-s}\log\left\{\int_{0}^{\infty }f^{s}(x,\alpha ,\beta )dx\right\},$$

where s(integer)>0 and s≠1. For the NTPQED, we have:

$$I_{R}\left(s\right)=\frac{1}{1-s}\log\left(\int_{0}^{\infty }{\left(\frac{\beta^{3}\left(\alpha +\beta x+x^{2}\right)}{\beta^{2}+\alpha \beta^{2}+2}\exp(-\beta x)\right)}^{s}dx\right)=\frac{1}{1-s}\log\left(\int_{0}^{\infty }\frac{\beta^{3s}}{{\left(\beta^{2}+\alpha \beta^{2}+2\right)}^{s}}{\left(\alpha +\beta x+x^{2}\right)}^{s}\exp(-s\beta x)dx\right).$$

We observe that

$$\int_{0}^{\infty }{\left(\alpha +\beta x+x^{2}\right)}^{s}\exp(-s\beta x)dx=\sum_{i=0}^{s}\frac{s!}{\left(s-i\right)!i!}\int_{0}^{\infty }{\left(\alpha +\beta x\right)}^{i}{\left(x^{2}\right)}^{\left(s-i\right)}e^{-\theta sx}dx=\sum_{i=0}^{s}\sum_{j=0}^{i}\frac{s!}{\left(s-i\right)!i!}\frac{i!}{\left(i-j\right)!j!}\alpha^{j}\beta^{i-j}\int_{0}^{\infty }x^{2s-i-j}e^{-\theta sx}dx,$$

where

$$\int_{0}^{\infty }x^{2s-i-j}e^{-\theta sx}dx={\left(s\theta \right)}^{j-2s+i-1}\Gamma \left(2s-j+1-i\right).$$

Now, the Rényi entropy observes as

$$I_{R}\left(s\right)=\frac{1}{1-s}\log(\frac{\beta^{3s}}{{\left(\beta^{2}+\alpha \beta^{2}+2\right)}^{s}}\sum_{i=0}^{s}\sum_{j=0}^{i}\frac{s!}{\left(s-i\right)!i!}\frac{i!}{\left(i-j\right)!j!}\alpha^{j}\beta^{i-j}\Gamma \left(2s-j+1-i\right){\left(s\theta \right)}^{j-2s+i-1}).$$

### 3.5 Fuzzy reliability

Let *T* be a continuous random variable that represents a system's failure time (component). According to Chen et al. [7], the fuzzy dependability can then be calculated using fuzzy probability, which is

$$R_{F}(t)=P\left(T>t\right)=\int_{t}^{\infty }\mu (x)\;f(x,\alpha ,\beta )dx,0\leq t\leq x<\infty ,$$

where

$$\mu (x)=\left\{ \begin{array}{cc} 0\; & ,x\leq t_{1}\\ \frac{x-t_{1}}{t_{2}-t_{1}}\; & ,t_{1}<x<t_{2}\\ 1\; & ,x\geq t_{2} \end{array}\right..$$

For μ(x), by the computational analysis of the function of fuzzy numbers, the lifetime x(λ) can be obtained corresponds to a certain value of λ−Cut,λ∈[0,1], can be obtained as: $\mu (x)=\lambda \to \;\frac{x-t_{1}}{t_{2}-t_{1}}=\lambda$, then

$$\left\{ \begin{array}{cc} x(\lambda )\leq t_{1}\; & ,\lambda =0\\ x(\lambda )=t_{1}+\lambda (t_{2}-t_{1})\; & ,0<\lambda <1\\ x(\lambda )\geq t_{2}\; & ,\lambda =1 \end{array}\right..$$

As a result, the fuzzy reliability values may be determined for all *λ* values. The fuzzy dependability of the NTPQED is determined by the fuzzy reliability definition. The fuzzy reliability of the NTPQED can be defined as,

$$R_{F}(t)=e^{-\beta t_{1}}\left(1+\frac{\beta^{2}t_{1}^{2}+\left(\beta^{3}+2\beta \right)t_{1}}{\beta^{2}+\alpha \beta^{2}+2}\right)-e^{-\beta x(\lambda )}\left(1+\frac{\beta^{2}x{\left(\lambda \right)}^{2}+\left(\beta^{3}+2\beta \right)x(\lambda )}{\beta^{2}+\alpha \beta^{2}+2}\right).$$

Then $R_{F}{\left(t\right)}_{\lambda =0}=0$.

#### 3.5.1 Numerical values of fuzzy reliability

In this subsection, we obtained a comparison between traditional reliability and Fuzzy reliability, where traditional reliability is a survival function as

$$S(x;\alpha ,\beta )=\left(1+\frac{\beta^{2}x^{2}+\left(\beta^{3}+2\beta \right)x}{\beta^{2}+\alpha \beta^{2}+2}\right)e^{-\beta x}.$$

Table 1 discussed in a comparison. The following observations are made based on the findings:

1. When the λ−Cut is increased the Fuzzy reliability increases.
2. When the $t_{2}$ of the interval of the membership function is increased, the Fuzzy reliability increases.
3. When the $t_{1}$ is decreased, the Fuzzy reliability increases, and vice versa.
4. The traditional reliability with $t_{2}$ is lower than the traditional reliability with $t_{1}$.

**Table 1:** Traditional and fuzzy reliability with different values.

						*R*_*F*_	*R*_*F*_	*R*_*F*_
*β*	*α*	*t*_1_	*t*_2_	*S*(*t*_1_)	*S*(*t*_2_)	*λ* = 0.25	*λ* = 0.55	*λ* = 0.90

0.75			1		0.90651	0.012842	0.037475	0.079196
0.75			1.5		0.82289	0.0021838	0.069185	0.15017
0.75	0.25	0.001	2	0.99996	0.72614	0.032662	0.10872	0.23419
0.75			1		0.90651	0.013275	0.0022727	0.077848
0.75			1.5		0.82289	0.022804	0.034652	0.149
0.75		0.05	2	0.99794	0.72614	0.034169	0.074754	0.23312

0.75		0.1	1		0.83485	0.035168	0.078975	0.13338
0.75		0.1	1.5		0.74065	0.055158	0.12626	0.21683
0.75		0.1	3	0.98443	0.45792	0.11833	0.28172	0.47550

0.75	1.2	0.2	1		0.83485	0.031557	0.070968	0.11961

0.75		0.2	2		0.64311	0.072655	0.16909	0.29061
0.75		0.2	4	0.96888	0.30641	0.16162	0.38177	0.60967
0.75		1	3		0.45792	0.094196	0.21117	0.34204
0.75		1	5	0.83485	0.19547	0.19173	0.4104	0.5997
1.5		0.2	1		0.50004	0.10772	0.22787	0.35350
1.5		0.2	1.5		0.32719	0.71164	0.3514	0.51942
1.5		0.2	3	0.88632	0.074047	0.34508	0.62334	0.78673
1.5		0.8	2		0.20560	0.12356	0.24559	0.35402
1.5		0.8	3		0.074047	0.21130	0.38100	0.4911
1.5		0.8	1	0.58462	0.50004	0.021964	0.047605	0.076521

The fuzzy values algorithm produces a series of draws from NTPQED.

**Algorithm 1:**
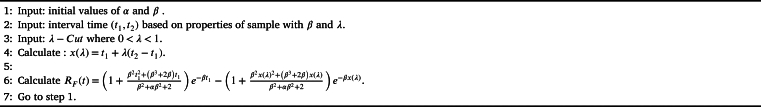
Fuzzy values algorithm.

### 3.6 Value at risk of the NTPQED (quantile function)

It may be noted that $F_{X}(x)$ in Eq. ((2) is continuous and strictly increasing, so the quantile function of *X* is defined

$$Q_{X}(u)=VaR=x_{u}=F_{X}^{-1}(u)\;u\epsilon \left[0.1\right].$$

Definition 1 Risk managers use value at risk (*VaR*) to measure and control the level of risk exposure. The mathematical definition is

VaR=inf⁡(x∈R,P(X>x≤1−u)),

where u∈(0,1) is the level. The formula tells us what the maximum loss we can expect with normal market conditions or what amount of loss we should not exceed with a given level of probability; thus, *VaR* is also known as a quantile risk measure and is defined as $VaR=F_{X}^{-1}(u)$ for a continuous distribution.

For $u=F_{X}(x)$, we can not give an explicit expression for $Q_{X}(u)$ (cannot use Lambert W function in our case), but we give a numerical solution in Table 2.

**Table 2:** Some numerical solutions of $F_{X}^{-1}(u)$.

*α*	*β*	*u*	$VaR=F_{X}^{-1}(u)$
0.5	0.1	0.1	10.901
		0.3	19.037
		0.5	26.647
		0.7	36.067
		0.9	53.139

1	0.2	0.1	5.1433
		0.3	9.2734
		0.5	13.103
		0.7	17.829
		0.9	26.380

1	1	0.1	0.3932
		0.3	1.1297
		0.5	1.8905
		0.7	2.8439
		0.9	4.5691

2	3	0.1	0.056027
		0.3	0.18574
		0.5	0.35253
		0.7	0.59476
		0.9	1.0863

### 3.7 Mean excess function

For a claim amount random variable *X*, the mean excess or residual life function (e(x)) is the expected payment per claim on a policy with a fixed amount deductible of *x*, where claims with amounts less than or equal to *x* is completely ignored.

It is defined for the NTPQED as follows

$$e(x)=E(X-x/X>x)=\frac{1}{1-F(x)}\int_{x}^{\infty }(1-F(u))du=\frac{1}{e^{{}^{-\beta x}}\left(1+\frac{\beta^{2}x^{2}+\left(\beta^{3}+2\beta \right)x}{\beta^{2}+\alpha \beta^{2}+2}\right)}\;\int_{x}^{\infty }e^{{}^{-\beta u}}\left(1+\frac{\beta^{2}u^{2}+\left(\beta^{3}+2\beta \right)u}{\beta^{2}+\alpha \beta^{2}+2}\right)du=\frac{\left(x^{2}\beta^{2}+2x\beta +\beta^{2}+x\beta^{3}+\alpha \beta^{2}+2\right)\left(x^{2}\beta^{2}+4x\beta +2\beta^{2}+x\beta^{3}+\alpha \beta^{2}+6\right)}{\beta {\left(\beta^{2}+\alpha \beta^{2}+2\right)}^{2}}.$$

A numerical representation for the mean excess function is presented in Table 3.

**Table 3:** Numerical representation of *e*(*x*).

*x*	*α*	*β*	*e*(*x*)
0.1	6	2	0.687 97
0.2	0.5	0.1	30.911
0.5	0.5	0.5	8.2438
1	1	1	7.5
2	2	1	14.4
3	2	5	18.268
4	0.2	5	197.63
5	0.1	0.1	66.836
10	5	2	239.88
50	6	2	64004

### 3.8 Limited expected value function

The limited expected value function *L* of a claim size variable *X* or of the corresponding CDF F(x), is defined as follows

$$L(u)=E\left(\min(X,u\right)=\int_{0}^{u}xdF(x)+u\left(1-F(u)\right),u>0.$$

The value of the function *L* at point *x* equals the expectation of the CDF F(x) tr*π*uncated. Given a policy limit or deductible from a reinsurance perspective, say *u*, the limited loss random variable is defined as follows.

$$X\wedge u=\min(X,u)=\left\{ \begin{array}{cc} X,\; & X\leq u\\ u,\; & X\;>u \end{array}\right..$$

The limited expected value function is defined as the expectation of the limited, which is calculated as follows

$$E(X\wedge u)=\int_{0}^{\infty }xdF(x)=\int_{0}^{u}xdF(x)+\int_{u}^{\infty }udF(x)=\int_{0}^{u}xf(x,\alpha ,\beta )dx+u\left({F(x)]}_{u}^{\infty }\right)=\int_{0}^{u}xf(x,\alpha ,\beta )dx+u\left(1-F(u)\right)=m_{1}(u)+u(1-F(u)),$$

where

$$m_{1}(u)=\int_{0}^{u}xf(x,\alpha ,\beta )dx=\int_{0}^{u}x\frac{\beta^{3}\left(\alpha +\beta x+x^{2}\right)}{\beta^{2}+\alpha \beta^{2}+2}\exp(-\beta x)dx=\frac{2\beta^{2}+\alpha \beta^{2}+6}{2\beta +\beta^{3}+\alpha \beta^{3}}-\frac{\left(3u^{2}\beta^{2}+u^{2}\beta^{4}+u^{3}\beta^{3}+6u\beta +2\beta^{2}+2u\beta^{3}+\alpha \beta^{2}+u\alpha \beta^{3}+6\right)}{2\beta +\beta^{3}+\alpha \beta^{3}}e^{-u\beta }.$$

Then, we have

$$E(X\wedge u)=\frac{1}{2\beta +\beta^{3}+\alpha \beta^{3}}(6+2\beta^{2}+\alpha \beta^{2}-(6+2\beta^{2}+\alpha \beta^{2}+\left(8\beta +3\beta^{3}+2\alpha \beta^{3}\right)u+\left(5\beta^{2}+2\beta^{4}\right)u^{2}+2\beta^{3}u^{3})e^{-u\beta }).$$

### 3.9 Tail value at risk

The tail value at risk (TVaR), also known as the tail conditional expectation, is a risk measure associated with the general value at risk. TVaR measures the expectation of the losses beyond VaR. The TVaR is defined as the NTPQED as follows

$$TVaR=E(X/X>VaR)=\frac{1}{1-p}\int_{VaR}^{\infty }xf(x,\alpha ,\beta )dx=\frac{1}{1-p}\int_{VaR}^{\infty }x\frac{\beta^{3}\left(\alpha +\beta x+x^{2}\right)}{\beta^{2}+\alpha \beta^{2}+2}e^{-\beta x}dx=e^{-VaR\beta }\frac{1}{\left(1-p)(2\beta +\beta^{3}+\alpha \beta^{3}\right)}\;\frac{3\beta^{2}VaR^{2}+\beta^{4}VaR^{2}+\beta^{3}VaR^{3}+6\beta VaR+2\beta^{3}VaR++\alpha \beta^{3}VaR+\alpha \beta^{2}+2\beta^{2}+6}{}.$$

Although it virtually always represents a loss, *VaR* is conventionally reported as a positive number.

### 3.10 Tail variance

Tail variance (TV) measures losses conditional variance, given that they exceed *VaR* at a given probability *P*. *TV* is defined for the NTPQED as follows

$$TV=E(X^{2}/X>VaR)-{\left(TVaR\right)}^{2}=\frac{e^{-\beta VaR}}{\left(1-p)(2\beta^{2}+\beta^{4}+\alpha \beta^{4}\right)}(12VaR^{2}\beta^{2}+3VaR^{2}\beta^{4}+4VaR^{3}\beta^{3}+VaR^{3}\beta^{5}+VaR^{4}\beta^{4}+24VaR\beta +6\beta^{2}+6VaR\beta^{3}+2\alpha \beta^{2}+VaR^{2}\alpha \beta^{4}+2VaR\alpha \beta^{3}+24)-{\left(TVaR\right)}^{2}.$$

## 4 Estimation of the NTPQED parameters

This section will focus on the estimation of the NTPQED parameters using various methods. We will obtain the estimator by maximizing or minimizing an objective function, as we will demonstrate.

### 4.1 Method of maximum likelihood (MLE)

Here, the parameters of the proposed model are estimated using the MLE method by optimizing the following log-likelihood function

$$\log L=3n\log\beta +\sum_{i=1}^{n}\log(\alpha +\beta x_{i}+x_{i}^{2})-n\log(\beta^{2}+\alpha \beta^{2}+2)-\beta \sum_{i=1}^{n}x_{i}.$$

### 4.2 Method of Anderson-Darling (ADE)

Suppose we have an ordered random sample of size n given by $X_{1},...,X_{n}$, where each $X_{i}$ is distributed as NTPQED(α,β). To estimate the parameters of the NTPQED using the ADE method, we must minimize the following expression concerning its parameters. For more detail, see [3].

$$A(x_{i})=-n-\frac{1}{n}\sum_{i=1}^{n}(2i-1)\left[\log F(x_{i:n})+\log S(x_{n-i-1:n})\right].$$

### 4.3 Method of Cramer_von_Mises (CVME)

To estimate the NTPQED unknown coefficients by using the CVME technique [8], we have to minimize the following equation

$$C(x_{i})=\frac{1}{12n}+\sum_{i=1}^{n}{\left[F(x_{i:n})-\frac{2i-1}{2n}\right]}^{2}.$$

### 4.4 Method of maximum product of spacings (MPSE)

Another well-known estimation method is MPSE, proposed by [12]. To estimate the model's parameters, we have to optimize the expression below

$$\delta \left(x_{i}\right)=\frac{1}{n+1}\sum_{i=1}^{n+1}\log\Lambda_{i}(x_{i}),$$

where $F_{i:n}$ is the CDF of NTPQED of the ordered variables, $\Lambda_{i}(x_{i})=F(x_{i:n})-F(x_{i-1:n})$, $F(x_{0:n})=0$ and $F(x_{n+1:n})=1$.

### 4.5 Methods of ordinary least squares (OLSE)

The OLSE is an alternative to the MLE method introduced by [22]. For the NTPQED model, the OLSEs, $\overset{ˆ}{\alpha }$, and $\overset{ˆ}{\beta }$ of *α* and *β* are defined as those arguments that minimize the objective function

$$V(x_{i})=\sum_{i=1}^{n}{\left[F(x_{i:n})-\frac{i}{n+1}\right]}^{2}=\sum_{i=1}^{n}{\left[F(x_{i:n})-\frac{i}{n+1}\right]}^{2}.$$

### 4.6 Methods of right_tail Anderson_Darling (RTADE)

To estimate the NTPQED distribution's coefficients using the RTADE technique [3], we have to minimize the following expression the following equation

$$R(x_{i})=\frac{n}{2}-2\sum_{i=1}^{n}F(x_{i:n})-\frac{1}{n}\sum_{i=1}^{n}(2i-1)\log S(x_{i:n}).$$

### 4.7 Methods of weighted least squares (WLSE)

To estimate the parameters of the suggested model, we have used another method known as WLSE. Using this method, we need to optimize the expression below

$$W(x_{i})=\sum_{i=1}^{n}\frac{{\left(n+1\right)}^{2}(n+2)}{i(n-i+1)}{\left[F(x_{i:n})-\frac{i}{n+1}\right]}^{2}.$$

### 4.8 Methods of left tail Anderson Darling (LTADE)

To estimate the parameters of the suggested model, we have used another method known as LTADE. Using this method, we need to optimize the expression below

$$L(x_{i})=-\frac{3}{2}n+2\sum_{i=1}^{n}F(x_{i:n})-\frac{1}{n}\sum_{i=1}^{n}(2i-1)\log F(x_{i:n}).$$

### 4.9 Minimum spacing absolute distance (MSADE)

To estimate the parameters of the NTPQED using the MSADE approach, we have to minimize the following equation

$$\zeta \left(x_{i}\right)=\sum_{i=1}^{n+1}|\Lambda_{i}-\frac{1}{n+1}|.$$

### 4.10 Minimum spacing absolute-log distance (MSALDE)

To estimate the NTPQED coefficients using the MSALDE approach, we have to optimize the following equation

$$ϒ\left(x_{i}\right)=\sum_{i=1}^{n+1}|\log\Lambda_{i}-\log\frac{1}{n+1}|.$$

### 4.11 Anderson Darling left tail second order (ADSOE)

To estimate the NTPQED distribution's coefficients using the ADSOE technique [1], we have to minimize the following expression the following equation

$$LTS=2\sum_{i=1}^{n}\log F(x_{i})+\frac{1}{n}\sum_{i=1}^{n}\frac{\left(2i-1\right)}{F(x_{i})}.$$

### 4.12 Kolmogorov Method (KE)

We have used another method known as KE to estimate the parameters of the suggested model. Using this method, we need to optimize the expression below

$$KM=\underset{1\leq i\leq n}{MAX}\left[\frac{i}{n}-F(x_{i}),F(x_{i})-\frac{i-1}{n}\right].$$

### 4.13 Minimum spacing square distance (MSSD)

To estimate the parameters of the suggested model, we have used another method known as MSSD. Using this method, we need to optimize the expression below

$$\phi_{\left(x_{i}\right)}=\sum_{i=1}^{n+1}(I_{i}^{2}-\frac{1}{n+1}).$$

### 4.14 Minimum spacing square-log distance (MSSLD)

To estimate the parameters of the suggested model, we have used another method known as MSSLD. Using this method, we need to optimize the expression below

$$\Psi_{\left(x_{i}\right)}=\sum_{i=1}^{n+1}{\left(\log I_{i}-\log\frac{1}{n+1}\right)}^{2}.$$

### 4.15 Minimum spacing Linex distance (MSLND)

To estimate the parameters of the suggested model, we have used another method known as MSLND [23]. Using this method, we need to optimize the expression below

$$\Delta_{\left(x_{i}\right)}=\sum_{i=1}^{n+1}\left[e^{I_{i}-\frac{1}{n+1}}-\left(I_{i}-\frac{1}{n+1}\right)-1\right].$$

## 5 Numerical simulation

This portion of the study undertakes an examination to assess the efficacy of various estimation methods detailed in Section 4. Random datasets were created utilizing our suggested model, after which these estimation techniques were utilized to unveil the recommended model estimators. The evaluation of estimation method efficacy in this investigation is based on the utilization of six specific metrics, delineated as follows:

1. The average of absolute bias (BIAS) is determined by the formula: $\left|Bias(\overset{ˆ}{\Xi })|=\frac{1}{M}\sum_{i=1}^{M}|\overset{ˆ}{\Xi }-\Xi \right|$.
2. The mean squared error (MSE) is calculated as follows: $MSE=\frac{1}{M}\sum_{i=1}^{M}{\left(\overset{ˆ}{\Xi }-\Xi \right)}^{2}$.
3. The mean absolute relative error (MRE) is evaluated using the expression: $MRE=\frac{1}{M}\sum_{i=1}^{M}|\overset{ˆ}{\Xi }-\Xi |/\Xi$.
4. The average absolute difference, denoted as $D_{abs}$, is given by: $D_{abs}=\frac{1}{nH}\sum_{i=1}^{H}\sum_{j=1}^{n}|F(x_{ij};\Xi )-F(x_{ij};\overset{ˆ}{\Xi })|$, where F(x;Ξ)=F(x) and $x_{ij}$ represents values obtained at the *i*-th iteration sample and *j*-th component of this sample.
5. The maximum absolute difference, represented by $D_{max}$, is derived from: $D_{max}=\frac{1}{H}\sum_{i=1}^{H}\max_{j=1,\ldots ,n}|F(x_{ij};\Xi )-F(x_{ij};\overset{ˆ}{\Xi })|$.
6. The average squared absolute error (ASAE) is computed as $ASAE=\frac{1}{H}\sum_{i=1}^{H}\frac{\left|x_{\left(i\right)}-{\overset{ˆ}{x}}_{\left(i\right)}\right|}{x_{\left(n\right)}-x_{\left(1\right)}}$, where $x_{\left(i\right)}$ denotes the ascending ordered observations, and Ξ=(α,β).

Another aim of this simulation investigation is to pinpoint the most effective estimation approach for computing our suggested model estimators. The simulation encompasses creating random samples varying in size from our model, establishing the metrics we employ, and iterating this procedure numerous times.

**Figure 2:**
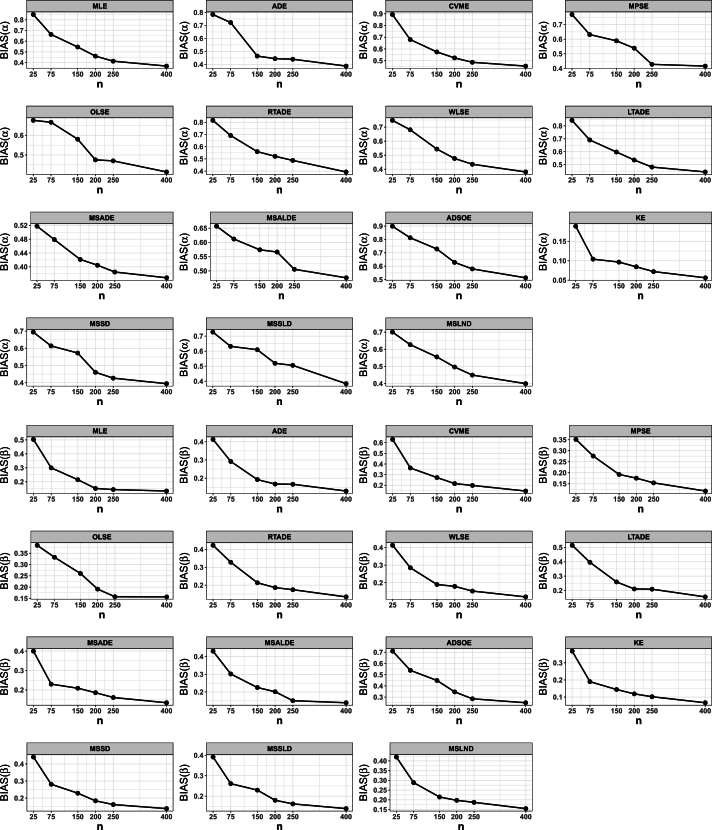
Graphical representation for BIAS values presented in Table 4.

**Figure 3:**
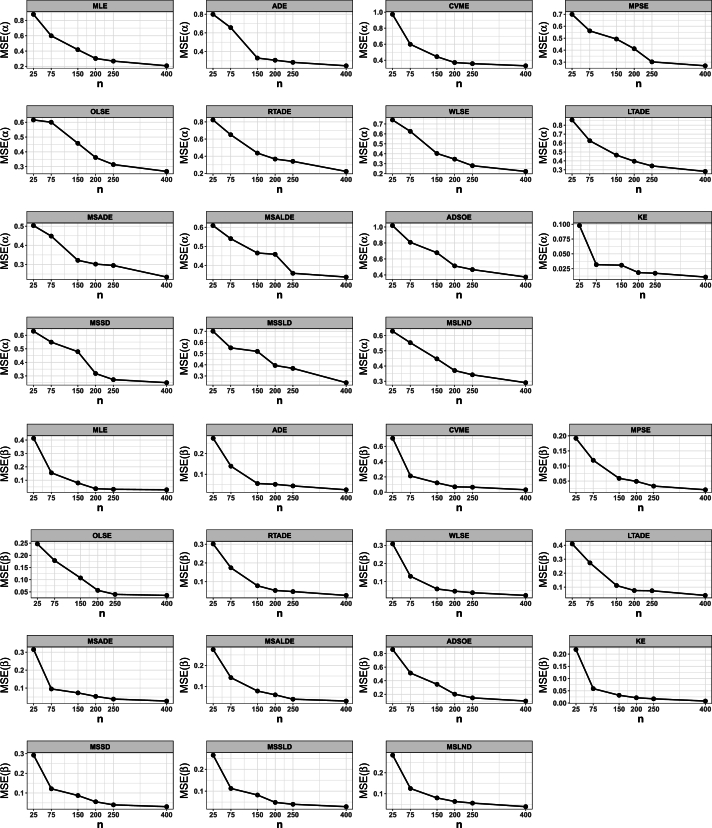
Graphical representation for MSE values presented in Table 4.

**Figure 4:**
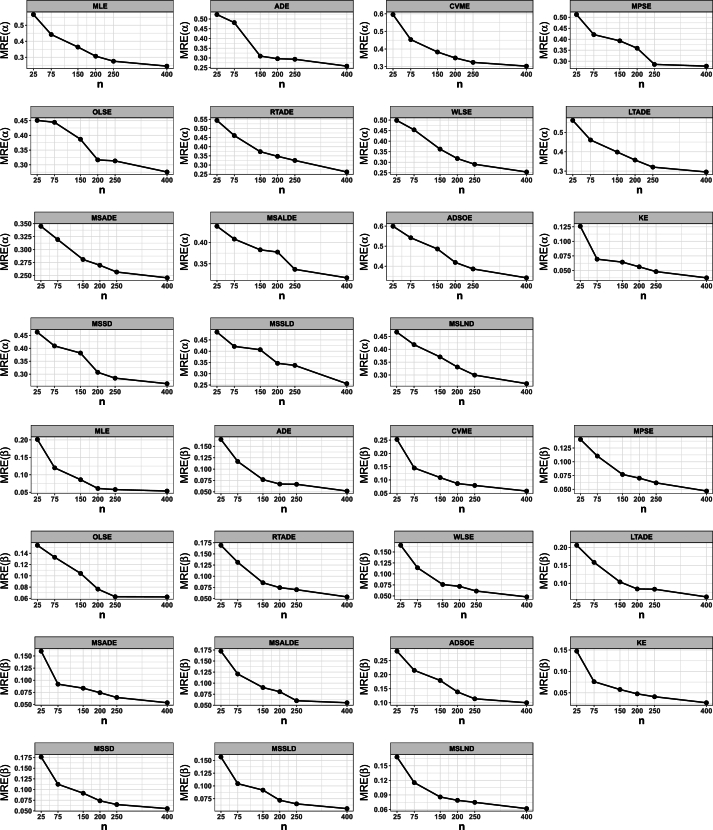
Graphical representation for MRE values presented in Table 4.

**Figure 5:**
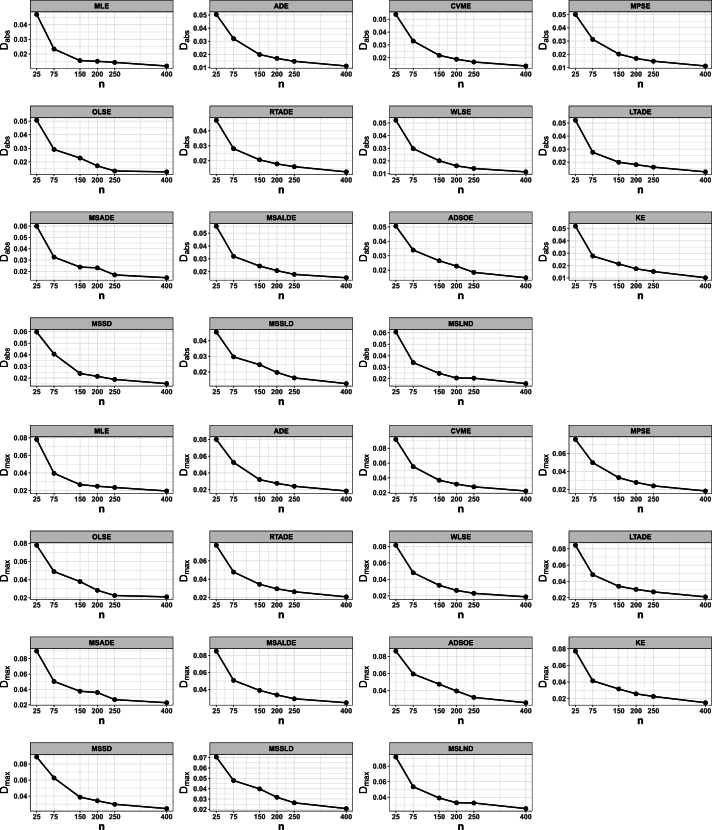
Graphical representation for *D*_*abs*_ and *D*_*max*_ values presented in Table 4.

**Figure 6:**
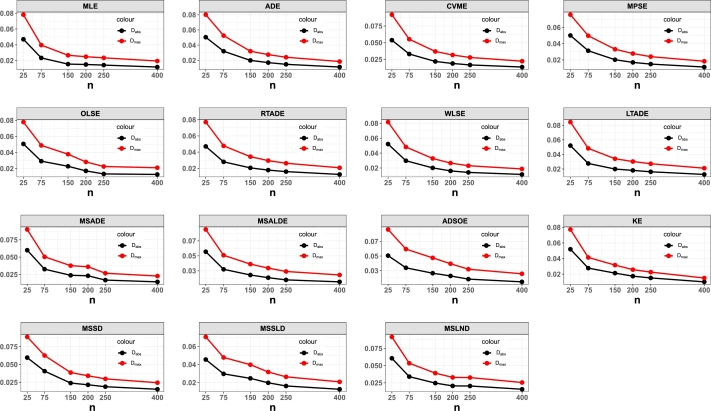
Comparison between *D*_*abs*_ and *D*_*max*_ values presented in Table 4.

**Figure 7:**
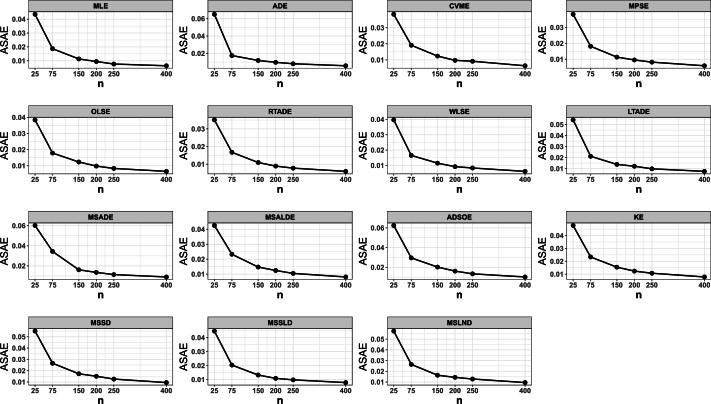
Graphical representation for ASAE values presented in Table 4.

Table 4, Table 5, Table 6, Table 7, Table 8 exhibit our simulation results. The data from Table 4 is further illustrated in Figure 2, Figure 3, Figure 4, Figure 5, Figure 6, Figure 7. The significance of each datum is determined by its rank compared to all estimation methods. Table 9 supplies our estimators' partial and total rankings. Table 4, Table 5, Table 6, Table 7, Table 8 showcase the outcomes of simulating the proposed model parameters using fifteen distinct estimation techniques. Key observations from these tables encompass the following:

1. It is notable that all parameter estimation methods for the proposed model exhibit high reliability, precisely approximating their true values.
2. Across all examined scenarios, the computed metrics consistently decrease as the sample size (*n*) increases.
3. Each estimation technique excels in determining the parameters of the proposed model.
4. According to our analysis, Kolmogorov estimation emerges as the most efficient method for evaluating the parameter values, achieving a total score of 36.0, as outlined in Table 9. The overall rankings for all estimation strategies are summarized in Table 9.

**Table 4:** Numerical values of simulation measures for *α* = 1.5, *β* = 2.5.

n	Est.	MLE	ADE	CVME	MPSE	OLSE	RTADE	WLSE	LTADE	MSADE	MSALDE	ADSOE	KE	MSSD	MSSLD	MSLND
25	BIAS($\overset{ˆ}{\alpha }$)	0.84996^{13}^	0.78436^{10}^	0.89406^{14}^	0.76891^{9}^	0.67625^{4}^	0.81597^{11}^	0.74796^{8}^	0.84295^{12}^	0.51759^{2}^	0.65686^{3}^	0.8989^{15}^	0.18894^{1}^	0.69437^{5}^	0.7271^{7}^	0.70033^{6}^
	BIAS($\overset{ˆ}{\beta }$)	0.50327^{12}^	0.41296^{7}^	0.63116^{14}^	0.35139^{1}^	0.38545^{3}^	0.42328^{9}^	0.41294^{6}^	0.51443^{13}^	0.39988^{5}^	0.43091^{10}^	0.70997^{15}^	0.36838^{2}^	0.44057^{11}^	0.39155^{4}^	0.42006^{8}^
	MSE($\overset{ˆ}{\alpha }$)	0.87982^{13}^	0.79904^{10}^	0.97301^{14}^	0.69944^{7}^	0.61547^{4}^	0.82289^{11}^	0.7407^{9}^	0.85869^{12}^	0.50328^{2}^	0.60901^{3}^	1.01816^{15}^	0.09777^{1}^	0.63026^{6}^	0.70109^{8}^	0.6292^{5}^
	MSE($\overset{ˆ}{\beta }$)	0.41312^{13}^	0.27344^{5}^	0.70535^{14}^	0.19187^{1}^	0.24663^{3}^	0.30162^{9}^	0.308^{10}^	0.40909^{12}^	0.31481^{11}^	0.27546^{6}^	0.85905^{15}^	0.21853^{2}^	0.29207^{8}^	0.26448^{4}^	0.28371^{7}^
	MRE($\overset{ˆ}{\alpha }$)	0.56664^{13}^	0.52291^{10}^	0.59604^{14}^	0.5126^{9}^	0.45084^{4}^	0.54398^{11}^	0.49864^{8}^	0.56197^{12}^	0.34506^{2}^	0.43791^{3}^	0.59927^{15}^	0.12596^{1}^	0.46291^{5}^	0.48473^{7}^	0.46688^{6}^
	MRE($\overset{ˆ}{\beta }$)	0.20131^{12}^	0.16518^{7}^	0.25246^{14}^	0.14056^{1}^	0.15418^{3}^	0.16931^{9}^	0.16517^{6}^	0.20577^{13}^	0.15995^{5}^	0.17237^{10}^	0.28399^{15}^	0.14735^{2}^	0.17623^{11}^	0.15662^{4}^	0.16802^{8}^
	*D*_*abs*_	0.04701^{2}^	0.05045^{5}^	0.0538^{11}^	0.05007^{4}^	0.05064^{7}^	0.04708^{3}^	0.05214^{9.5}^	0.05214^{9.5}^	0.05999^{14}^	0.05548^{12}^	0.05055^{6}^	0.05195^{8}^	0.05966^{13}^	0.04555^{1}^	0.06067^{15}^
	*D*_*max*_	0.07822^{6}^	0.0801^{7}^	0.09199^{15}^	0.07566^{2}^	0.07784^{5}^	0.07705^{3}^	0.08162^{8}^	0.08441^{9}^	0.08984^{13}^	0.08512^{10}^	0.08631^{11}^	0.07723^{4}^	0.08875^{12}^	0.07064^{1}^	0.0917^{14}^
	ASAE	0.0435^{6}^	0.06505^{7}^	0.03836^{15}^	0.03828^{2}^	0.03844^{5}^	0.0351^{3}^	0.03967^{8}^	0.05419^{9}^	0.0602^{13}^	0.04253^{10}^	0.06245^{11}^	0.04784^{4}^	0.05503^{12}^	0.04468^{1}^	0.05755^{14}^
	∑*Ranks*	91^{12}^	76^{9}^	113^{14}^	36^{2}^	37^{3}^	67^{6.5}^	69.5^{8}^	102.5^{13}^	67^{6.5}^	63^{5}^	121^{15}^	30^{1}^	82^{11}^	44^{4}^	81^{10}^
75	BIAS($\overset{ˆ}{\alpha }$)	0.66325^{8}^	0.72233^{14}^	0.6802^{10}^	0.63149^{6}^	0.66649^{9}^	0.69168^{13}^	0.68079^{11}^	0.69101^{12}^	0.479^{2}^	0.61192^{3}^	0.81256^{15}^	0.10419^{1}^	0.61398^{4}^	0.63191^{7}^	0.62697^{5}^
	BIAS($\overset{ˆ}{\beta }$)	0.29976^{9}^	0.29189^{8}^	0.36265^{13}^	0.27607^{4}^	0.33237^{12}^	0.32805^{11}^	0.28504^{6}^	0.39592^{14}^	0.22977^{2}^	0.30172^{10}^	0.53883^{15}^	0.18997^{1}^	0.28108^{5}^	0.26105^{3}^	0.2883^{7}^
	MSE($\overset{ˆ}{\alpha }$)	0.59945^{9}^	0.65618^{14}^	0.59908^{8}^	0.56079^{7}^	0.60004^{10}^	0.65039^{13}^	0.62607^{12}^	0.62546^{11}^	0.44833^{2}^	0.54028^{3}^	0.80898^{15}^	0.0318^{1}^	0.54963^{4}^	0.55168^{5}^	0.55357^{6}^
	MSE($\overset{ˆ}{\beta }$)	0.1551^{10}^	0.13923^{8}^	0.21277^{13}^	0.11903^{4}^	0.1785^{12}^	0.17414^{11}^	0.12958^{7}^	0.27345^{14}^	0.095^{2}^	0.14231^{9}^	0.51363^{15}^	0.05894^{1}^	0.12141^{5}^	0.11243^{3}^	0.12502^{6}^
	MRE($\overset{ˆ}{\alpha }$)	0.44216^{8}^	0.48156^{14}^	0.45347^{10}^	0.42099^{6}^	0.44433^{9}^	0.46112^{13}^	0.45386^{11}^	0.46068^{12}^	0.31933^{2}^	0.40795^{3}^	0.54171^{15}^	0.06946^{1}^	0.40932^{4}^	0.42127^{7}^	0.41798^{5}^
	MRE($\overset{ˆ}{\beta }$)	0.1199^{9}^	0.11676^{8}^	0.14506^{13}^	0.11043^{4}^	0.13295^{12}^	0.13122^{11}^	0.11402^{6}^	0.15837^{14}^	0.09191^{2}^	0.12069^{10}^	0.21553^{15}^	0.07599^{1}^	0.11243^{5}^	0.10442^{3}^	0.11532^{7}^
	*D*_*abs*_	0.02337^{1}^	0.03199^{10}^	0.03304^{12}^	0.03127^{8}^	0.02919^{5}^	0.02807^{4}^	0.02976^{7}^	0.0276^{2}^	0.03261^{11}^	0.03198^{9}^	0.03394^{13}^	0.02776^{3}^	0.0407^{15}^	0.02965^{6}^	0.03404^{14}^
	*D*_*max*_	0.03962^{1}^	0.05255^{11}^	0.05524^{13}^	0.04975^{8}^	0.04883^{7}^	0.04779^{3}^	0.04819^{5}^	0.04833^{6}^	0.05044^{9}^	0.05074^{10}^	0.05949^{14}^	0.0414^{2}^	0.06263^{15}^	0.04785^{4}^	0.05349^{12}^
	ASAE	0.0187^{1}^	0.01753^{11}^	0.01911^{13}^	0.01817^{8}^	0.01787^{7}^	0.01674^{3}^	0.01654^{5}^	0.02121^{6}^	0.03437^{9}^	0.0233^{10}^	0.02954^{14}^	0.02338^{2}^	0.02657^{15}^	0.02034^{4}^	0.02647^{12}^
	∑*Ranks*	61^{5}^	90^{12}^	99^{14}^	52^{4}^	80^{10}^	81^{11}^	66^{6}^	94^{13}^	47^{3}^	67^{7}^	131^{15}^	22^{1}^	70^{8}^	46^{2}^	74^{9}^
150	BIAS($\overset{ˆ}{\alpha }$)	0.54583^{5}^	0.46439^{3}^	0.57447^{10}^	0.58893^{12}^	0.58034^{11}^	0.55955^{7}^	0.54371^{4}^	0.59773^{13}^	0.42136^{2}^	0.57405^{9}^	0.72875^{15}^	0.09657^{1}^	0.57273^{8}^	0.60974^{14}^	0.55537^{6}^
	BIAS($\overset{ˆ}{\beta }$)	0.21501^{8}^	0.19215^{3}^	0.2726^{14}^	0.19243^{4}^	0.2607^{13}^	0.21335^{6}^	0.19004^{2}^	0.25957^{12}^	0.20889^{5}^	0.22523^{9}^	0.44877^{15}^	0.14406^{1}^	0.22871^{10}^	0.22946^{11}^	0.21448^{7}^
	MSE($\overset{ˆ}{\alpha }$)	0.41821^{5}^	0.33026^{3}^	0.44437^{7}^	0.49305^{13}^	0.45775^{9}^	0.43703^{6}^	0.40319^{4}^	0.46495^{10}^	0.32165^{2}^	0.46504^{11}^	0.67821^{15}^	0.03086^{1}^	0.47883^{12}^	0.52018^{14}^	0.44722^{8}^
	MSE($\overset{ˆ}{\beta }$)	0.07939^{8}^	0.05558^{2}^	0.12201^{14}^	0.05896^{3}^	0.10718^{12}^	0.07806^{6}^	0.05965^{4}^	0.11191^{13}^	0.07303^{5}^	0.07893^{7}^	0.34759^{15}^	0.0318^{1}^	0.08715^{11}^	0.08234^{10}^	0.07999^{9}^
	MRE($\overset{ˆ}{\alpha }$)	0.36389^{5}^	0.30959^{3}^	0.38298^{10}^	0.39262^{12}^	0.3869^{11}^	0.37303^{7}^	0.36248^{4}^	0.39848^{13}^	0.2809^{2}^	0.3827^{9}^	0.48583^{15}^	0.06438^{1}^	0.38182^{8}^	0.40649^{14}^	0.37025^{6}^
	MRE($\overset{ˆ}{\beta }$)	0.086^{8}^	0.07686^{3}^	0.10904^{14}^	0.07697^{4}^	0.10428^{13}^	0.08534^{6}^	0.07602^{2}^	0.10383^{12}^	0.08356^{5}^	0.09009^{9}^	0.17951^{15}^	0.05762^{1}^	0.09148^{10}^	0.09178^{11}^	0.08579^{7}^
	*D*_*abs*_	0.01551^{1}^	0.0199^{2}^	0.02189^{8}^	0.02025^{5}^	0.02284^{9}^	0.02062^{6}^	0.02018^{4}^	0.01997^{3}^	0.0238^{10}^	0.02438^{12}^	0.0265^{15}^	0.02135^{7}^	0.02397^{11}^	0.02465^{14}^	0.02463^{13}^
	*D*_*max*_	0.02672^{1}^	0.03204^{3}^	0.03687^{8}^	0.03318^{5}^	0.03785^{10}^	0.03444^{7}^	0.03286^{4}^	0.0341^{6}^	0.0377^{9}^	0.03893^{12}^	0.04751^{15}^	0.03165^{2}^	0.0387^{11}^	0.03981^{14}^	0.0391^{13}^
	ASAE	0.01134^{1}^	0.01186^{3}^	0.01238^{8}^	0.01137^{5}^	0.01235^{10}^	0.01096^{7}^	0.01148^{4}^	0.01386^{6}^	0.01611^{9}^	0.01476^{12}^	0.02023^{15}^	0.01545^{2}^	0.01727^{11}^	0.01322^{14}^	0.01636^{13}^
	∑*Ranks*	43^{4}^	27^{2}^	92^{11}^	61^{7}^	94^{12}^	52^{5.5}^	32^{3}^	91^{10}^	52^{5.5}^	88^{9}^	135^{15}^	26^{1}^	95^{13}^	110^{14}^	82^{8}^
200	BIAS($\overset{ˆ}{\alpha }$)	0.46046^{4}^	0.44431^{3}^	0.52353^{11}^	0.53874^{13}^	0.47503^{6}^	0.52147^{10}^	0.47644^{7}^	0.536^{12}^	0.40466^{2}^	0.56626^{14}^	0.62841^{15}^	0.08444^{1}^	0.46073^{5}^	0.51945^{9}^	0.49665^{8}^
	BIAS($\overset{ˆ}{\beta }$)	0.15225^{2}^	0.16785^{3}^	0.21604^{14}^	0.17557^{4}^	0.19115^{10}^	0.1861^{9}^	0.1793^{5}^	0.21086^{13}^	0.18573^{8}^	0.20144^{12}^	0.34666^{15}^	0.11871^{1}^	0.18408^{7}^	0.17984^{6}^	0.19733^{11}^
	MSE($\overset{ˆ}{\alpha }$)	0.3035^{3}^	0.30722^{4}^	0.37114^{10}^	0.41211^{13}^	0.36191^{7}^	0.3671^{8}^	0.34588^{6}^	0.39657^{12}^	0.30197^{2}^	0.45833^{14}^	0.51365^{15}^	0.01843^{1}^	0.31792^{5}^	0.39391^{11}^	0.37045^{9}^
	MSE($\overset{ˆ}{\beta }$)	0.03649^{2}^	0.05173^{6}^	0.06995^{13}^	0.04899^{5}^	0.05644^{10}^	0.05311^{7}^	0.0474^{3}^	0.07605^{14}^	0.05355^{8}^	0.06073^{11}^	0.20269^{15}^	0.02155^{1}^	0.05544^{9}^	0.04862^{4}^	0.06311^{12}^
	MRE($\overset{ˆ}{\alpha }$)	0.30697^{4}^	0.29621^{3}^	0.34902^{11}^	0.35916^{13}^	0.31669^{6}^	0.34765^{10}^	0.31762^{7}^	0.35733^{12}^	0.26977^{2}^	0.3775^{14}^	0.41894^{15}^	0.05629^{1}^	0.30715^{5}^	0.3463^{9}^	0.3311^{8}^
	MRE($\overset{ˆ}{\beta }$)	0.0609^{2}^	0.06714^{3}^	0.08642^{14}^	0.07023^{4}^	0.07646^{10}^	0.07444^{9}^	0.07172^{5}^	0.08434^{13}^	0.07429^{8}^	0.08057^{12}^	0.13866^{15}^	0.04748^{1}^	0.07363^{7}^	0.07194^{6}^	0.07893^{11}^
	*D*_*abs*_	0.01505^{1}^	0.01699^{4}^	0.01885^{9}^	0.01693^{3}^	0.0171^{5}^	0.01787^{7}^	0.01615^{2}^	0.01809^{8}^	0.02311^{15}^	0.02081^{12}^	0.02278^{14}^	0.01745^{6}^	0.02151^{13}^	0.01974^{10}^	0.02057^{11}^
	*D*_*max*_	0.0249^{1}^	0.02751^{4}^	0.03157^{9}^	0.02784^{5}^	0.02825^{6}^	0.02954^{7}^	0.02663^{3}^	0.03029^{8}^	0.0361^{14}^	0.03362^{12}^	0.03949^{15}^	0.02578^{2}^	0.03417^{13}^	0.03172^{10}^	0.0329^{11}^
	ASAE	0.00949^{1}^	0.00971^{4}^	0.00973^{9}^	0.00956^{5}^	0.00981^{6}^	0.00894^{7}^	0.00921^{3}^	0.0122^{8}^	0.01352^{14}^	0.01244^{12}^	0.01613^{15}^	0.0124^{2}^	0.01508^{13}^	0.01085^{10}^	0.01437^{11}^
	∑*Ranks*	22^{1}^	35^{3}^	97^{12}^	64^{5}^	67^{6}^	68^{7}^	40^{4}^	101^{13}^	71^{8}^	112^{14}^	134^{15}^	24^{2}^	78^{10}^	73^{9}^	94^{11}^
250	BIAS($\overset{ˆ}{\alpha }$)	0.41342^{3}^	0.43994^{7}^	0.48613^{11}^	0.42796^{5}^	0.46988^{9}^	0.48752^{12}^	0.43565^{6}^	0.48105^{10}^	0.385^{2}^	0.50562^{14}^	0.57959^{15}^	0.07213^{1}^	0.42665^{4}^	0.50555^{13}^	0.44954^{8}^
	BIAS($\overset{ˆ}{\beta }$)	0.14447^{2}^	0.16657^{10}^	0.19848^{13}^	0.15421^{5}^	0.15729^{6}^	0.17446^{11}^	0.15265^{4}^	0.20916^{14}^	0.1611^{7}^	0.15069^{3}^	0.28519^{15}^	0.1019^{1}^	0.16228^{9}^	0.16206^{8}^	0.18692^{12}^
	MSE($\overset{ˆ}{\alpha }$)	0.26912^{2}^	0.28436^{5}^	0.35749^{12}^	0.30205^{7}^	0.31437^{8}^	0.34038^{9}^	0.27969^{4}^	0.3434^{11}^	0.29467^{6}^	0.35885^{13}^	0.46743^{15}^	0.01728^{1}^	0.27247^{3}^	0.36815^{14}^	0.34243^{10}^
	MSE($\overset{ˆ}{\beta }$)	0.032^{2}^	0.04394^{10}^	0.06551^{13}^	0.03331^{3}^	0.03986^{7}^	0.04707^{11}^	0.03889^{5}^	0.07437^{14}^	0.03856^{4}^	0.04012^{9}^	0.14789^{15}^	0.01729^{1}^	0.03971^{6}^	0.03997^{8}^	0.05543^{12}^
	MRE($\overset{ˆ}{\alpha }$)	0.27562^{3}^	0.29329^{7}^	0.32408^{11}^	0.28531^{5}^	0.31325^{9}^	0.32502^{12}^	0.29043^{6}^	0.3207^{10}^	0.25667^{2}^	0.33708^{14}^	0.38639^{15}^	0.04809^{1}^	0.28443^{4}^	0.33703^{13}^	0.2997^{8}^
	MRE($\overset{ˆ}{\beta }$)	0.05779^{2}^	0.06663^{10}^	0.07939^{13}^	0.06168^{5}^	0.06292^{6}^	0.06978^{11}^	0.06106^{4}^	0.08366^{14}^	0.06444^{7}^	0.06027^{3}^	0.11408^{15}^	0.04076^{1}^	0.06491^{9}^	0.06482^{8}^	0.07477^{12}^
	*D*_*abs*_	0.01427^{3}^	0.01476^{4}^	0.01669^{10}^	0.01487^{5}^	0.01332^{1}^	0.01602^{7}^	0.01411^{2}^	0.0162^{8}^	0.01689^{11}^	0.01786^{12}^	0.01843^{13}^	0.01519^{6}^	0.01884^{14}^	0.01622^{9}^	0.02044^{15}^
	*D*_*max*_	0.02346^{4}^	0.02408^{6}^	0.02789^{11}^	0.02402^{5}^	0.02253^{1}^	0.02632^{7}^	0.02309^{3}^	0.02721^{10}^	0.0268^{9}^	0.02908^{12}^	0.03197^{14}^	0.02264^{2}^	0.02984^{13}^	0.02636^{8}^	0.03268^{15}^
	ASAE	0.00767^{4}^	0.00804^{6}^	0.00915^{11}^	0.00819^{5}^	0.00834^{1}^	0.00777^{7}^	0.00832^{3}^	0.00985^{10}^	0.01134^{9}^	0.0105^{12}^	0.01335^{14}^	0.01075^{2}^	0.01258^{13}^	0.00974^{8}^	0.01281^{15}^
	∑*Ranks*	22^{1}^	62^{7}^	101^{13}^	44^{4}^	53^{5}^	82^{9}^	39^{3}^	100^{12}^	60^{6}^	90^{11}^	132^{15}^	25^{2}^	75^{8}^	89^{10}^	106^{14}^
400	BIAS($\overset{ˆ}{\alpha }$)	0.36741^{2}^	0.38711^{6}^	0.4539^{13}^	0.41608^{11}^	0.41353^{10}^	0.39302^{7}^	0.3808^{4}^	0.44335^{12}^	0.36822^{3}^	0.47579^{14}^	0.51318^{15}^	0.05612^{1}^	0.39452^{8}^	0.38373^{5}^	0.39937^{9}^
	BIAS($\overset{ˆ}{\beta }$)	0.13388^{5}^	0.12949^{4}^	0.14598^{11}^	0.11714^{2}^	0.15658^{14}^	0.13423^{7}^	0.11952^{3}^	0.15566^{13}^	0.13396^{6}^	0.13914^{10}^	0.25005^{15}^	0.06677^{1}^	0.13858^{8}^	0.13897^{9}^	0.15473^{12}^
	MSE($\overset{ˆ}{\alpha }$)	0.2079^{2}^	0.24727^{7}^	0.33051^{13}^	0.26929^{10}^	0.26789^{9}^	0.22296^{4}^	0.22209^{3}^	0.28215^{11}^	0.23421^{5}^	0.33899^{14}^	0.37399^{15}^	0.01086^{1}^	0.25012^{8}^	0.24008^{6}^	0.29065^{12}^
	MSE($\overset{ˆ}{\beta }$)	0.02842^{7}^	0.02535^{4}^	0.03132^{10}^	0.02105^{2}^	0.03564^{12}^	0.02659^{5}^	0.02399^{3}^	0.04103^{14}^	0.02758^{6}^	0.03139^{11}^	0.10143^{15}^	0.00825^{1}^	0.03062^{9}^	0.02909^{8}^	0.03845^{13}^
	MRE($\overset{ˆ}{\alpha }$)	0.24494^{2}^	0.25807^{6}^	0.3026^{13}^	0.27738^{11}^	0.27569^{10}^	0.26201^{7}^	0.25387^{4}^	0.29557^{12}^	0.24548^{3}^	0.31719^{14}^	0.34212^{15}^	0.03741^{1}^	0.26301^{8}^	0.25582^{5}^	0.26625^{9}^
	MRE($\overset{ˆ}{\beta }$)	0.05355^{5}^	0.0518^{4}^	0.05839^{11}^	0.04686^{2}^	0.06263^{14}^	0.05369^{7}^	0.04781^{3}^	0.06227^{13}^	0.05358^{6}^	0.05566^{10}^	0.10002^{15}^	0.02671^{1}^	0.05543^{8}^	0.05559^{9}^	0.06189^{12}^
	*D*_*abs*_	0.01182^{5}^	0.01119^{2}^	0.01355^{10}^	0.01125^{3}^	0.01267^{9}^	0.01254^{6}^	0.0114^{4}^	0.01256^{7}^	0.01437^{11}^	0.01525^{13.5}^	0.0147^{12}^	0.01017^{1}^	0.01525^{13.5}^	0.01257^{8}^	0.01569^{15}^
	*D*_*max*_	0.01949^{5}^	0.01841^{3}^	0.0223^{10}^	0.01835^{2}^	0.02102^{8}^	0.02074^{6}^	0.01878^{4}^	0.02113^{9}^	0.02277^{11}^	0.02445^{12.5}^	0.02572^{15}^	0.01507^{1}^	0.02445^{12.5}^	0.02077^{7}^	0.02547^{14}^
	ASAE	0.00642^{5}^	0.00574^{3}^	0.00635^{10}^	0.00591^{2}^	0.00653^{8}^	0.00595^{6}^	0.00613^{4}^	0.00754^{9}^	0.00898^{11}^	0.00812^{12.5}^	0.01017^{15}^	0.00784^{1}^	0.00951^{12.5}^	0.00783^{7}^	0.00951^{14}^
	∑*Ranks*	39^{4}^	37^{3}^	96^{11}^	45^{5}^	93^{10}^	52^{6}^	32^{2}^	99^{12}^	63^{7}^	110^{13.5}^	132^{15}^	18^{1}^	88^{9}^	66^{8}^	110^{13.5}^

**Table 5:** Numerical values of simulation measures for *α* = 0.6, *β* = 0.9.

n	Est.	MLE	ADE	CVME	MPSE	OLSE	RTADE	WLSE	LTADE	MSADE	MSALDE	ADSOE	KE	MSSD	MSSLD	MSLND
25	BIAS($\overset{ˆ}{\alpha }$)	0.31099^{14}^	0.30314^{12}^	0.30212^{11}^	0.3109^{13}^	0.29499^{10}^	0.31592^{15}^	0.29348^{9}^	0.29286^{8}^	0.2041^{2}^	0.27494^{4}^	0.29052^{7}^	0.07656^{1}^	0.2897^{6}^	0.28569^{5}^	0.27358^{3}^
	BIAS($\overset{ˆ}{\beta }$)	0.10098^{10}^	0.09826^{7}^	0.09585^{4}^	0.08562^{2}^	0.10739^{12}^	0.09809^{6}^	0.09638^{5}^	0.10228^{11}^	0.10047^{9}^	0.11688^{14}^	0.1216^{15}^	0.08304^{1}^	0.11417^{13}^	0.09041^{3}^	0.09868^{8}^
	MSE($\overset{ˆ}{\alpha }$)	0.12682^{14}^	0.12171^{12}^	0.11798^{9}^	0.12648^{13}^	0.11629^{8}^	0.12954^{15}^	0.11444^{5}^	0.11525^{6}^	0.07583^{2}^	0.10429^{4}^	0.11938^{11}^	0.01654^{1}^	0.11814^{10}^	0.11566^{7}^	0.10345^{3}^
	MSE($\overset{ˆ}{\beta }$)	0.01653^{11}^	0.01437^{6}^	0.01363^{4}^	0.01159^{2}^	0.01934^{12}^	0.01433^{5}^	0.01494^{7}^	0.01526^{8}^	0.0162^{10}^	0.02203^{13}^	0.02909^{15}^	0.01107^{1}^	0.0237^{14}^	0.01289^{3}^	0.01551^{9}^
	MRE($\overset{ˆ}{\alpha }$)	0.51832^{14}^	0.50523^{12}^	0.50354^{11}^	0.51816^{13}^	0.49166^{10}^	0.52653^{15}^	0.48913^{9}^	0.48809^{8}^	0.34017^{2}^	0.45823^{4}^	0.4842^{7}^	0.1276^{1}^	0.48283^{6}^	0.47615^{5}^	0.45597^{3}^
	MRE($\overset{ˆ}{\beta }$)	0.1122^{10}^	0.10918^{7}^	0.1065^{4}^	0.09513^{2}^	0.11932^{12}^	0.10899^{6}^	0.10709^{5}^	0.11364^{11}^	0.11164^{9}^	0.12986^{14}^	0.13511^{15}^	0.09227^{1}^	0.12686^{13}^	0.10046^{3}^	0.10964^{8}^
	*D*_*abs*_	0.04838^{6}^	0.05006^{7}^	0.04664^{4}^	0.04523^{2}^	0.05192^{11}^	0.04372^{1}^	0.05108^{10}^	0.05016^{8}^	0.05433^{12}^	0.06238^{15}^	0.05592^{13}^	0.04577^{3}^	0.05782^{14}^	0.04762^{5}^	0.05022^{9}^
	*D*_*max*_	0.07262^{7}^	0.07459^{8}^	0.07083^{5}^	0.06534^{1}^	0.07718^{11}^	0.06729^{3}^	0.0755^{10}^	0.07495^{9}^	0.07891^{12}^	0.08972^{15}^	0.08469^{14}^	0.06591^{2}^	0.08307^{13}^	0.06924^{4}^	0.07222^{6}^
	ASAE	0.38677^{7}^	0.4611^{8}^	0.37814^{5}^	0.2689^{1}^	0.38192^{11}^	0.49742^{3}^	0.3282^{10}^	0.29666^{9}^	0.23485^{12}^	0.36018^{15}^	0.69074^{14}^	0.04578^{2}^	0.68085^{13}^	0.69895^{4}^	0.235^{6}^
	∑*Ranks*	96^{13}^	82^{10}^	60^{5.5}^	52^{3.5}^	95^{12}^	78^{9}^	66^{7}^	74^{8}^	60^{5.5}^	90^{11}^	111^{15}^	12^{1}^	102^{14}^	50^{2}^	52^{3.5}^
75	BIAS($\overset{ˆ}{\alpha }$)	0.24797^{5}^	0.27087^{12}^	0.27032^{11}^	0.21725^{3}^	0.28219^{14}^	0.30594^{15}^	0.26554^{10}^	0.2582^{7}^	0.20355^{2}^	0.25358^{6}^	0.27942^{13}^	0.05926^{1}^	0.26146^{9}^	0.24444^{4}^	0.25998^{8}^
	BIAS($\overset{ˆ}{\beta }$)	0.05091^{2}^	0.05463^{4}^	0.05567^{5}^	0.05619^{6}^	0.05896^{10}^	0.06236^{12}^	0.05376^{3}^	0.0583^{9}^	0.06888^{13}^	0.05828^{8}^	0.07944^{15}^	0.04427^{1}^	0.06114^{11}^	0.05819^{7}^	0.06915^{14}^
	MSE($\overset{ˆ}{\alpha }$)	0.09081^{5}^	0.10059^{10}^	0.101^{12}^	0.07063^{3}^	0.10696^{13}^	0.12435^{15}^	0.09725^{9}^	0.09253^{7}^	0.07022^{2}^	0.09466^{8}^	0.10792^{14}^	0.01146^{1}^	0.10062^{11}^	0.08405^{4}^	0.09083^{6}^
	MSE($\overset{ˆ}{\beta }$)	0.00421^{2}^	0.00488^{6}^	0.00425^{3}^	0.00463^{5}^	0.00568^{10}^	0.00668^{12}^	0.00446^{4}^	0.00511^{8}^	0.00735^{13}^	0.00519^{9}^	0.01056^{15}^	0.00333^{1}^	0.006^{11}^	0.00502^{7}^	0.00772^{14}^
	MRE($\overset{ˆ}{\alpha }$)	0.41328^{5}^	0.45145^{12}^	0.45054^{11}^	0.36209^{3}^	0.47032^{14}^	0.5099^{15}^	0.44257^{10}^	0.43033^{7}^	0.33925^{2}^	0.42264^{6}^	0.46569^{13}^	0.09877^{1}^	0.43576^{9}^	0.4074^{4}^	0.4333^{8}^
	MRE($\overset{ˆ}{\beta }$)	0.05656^{2}^	0.0607^{4}^	0.06186^{5}^	0.06243^{6}^	0.06552^{10}^	0.06929^{12}^	0.05973^{3}^	0.06478^{9}^	0.07653^{13}^	0.06476^{8}^	0.08827^{15}^	0.04919^{1}^	0.06794^{11}^	0.06466^{7}^	0.07684^{14}^
	*D*_*abs*_	0.02351^{1}^	0.02543^{3}^	0.02637^{4}^	0.02766^{6}^	0.02988^{9}^	0.02825^{7}^	0.0274^{5}^	0.02992^{10}^	0.0357^{14}^	0.02859^{8}^	0.03659^{15}^	0.02426^{2}^	0.03201^{12}^	0.03128^{11}^	0.03512^{13}^
	*D*_*max*_	0.03667^{2}^	0.04041^{3}^	0.04122^{4}^	0.04215^{5}^	0.04643^{10}^	0.04489^{8}^	0.04299^{6}^	0.04601^{9}^	0.05337^{14}^	0.04412^{7}^	0.05676^{15}^	0.03504^{1}^	0.04875^{12}^	0.04687^{11}^	0.05265^{13}^
	ASAE	0.1486^{2}^	0.34432^{3}^	0.36124^{4}^	0.03422^{5}^	0.271^{10}^	0.27978^{8}^	0.16092^{6}^	0.11507^{9}^	0.02741^{14}^	0.14818^{7}^	0.16689^{15}^	0.04552^{1}^	0.1023^{12}^	0.07065^{11}^	0.09259^{13}^
	∑*Ranks*	33^{2}^	68^{6.5}^	70^{8}^	39^{3}^	102^{13}^	109^{14}^	60^{5}^	73^{9}^	74^{10}^	68^{6.5}^	126^{15}^	12^{1}^	92^{11}^	59^{4}^	95^{12}^
150	BIAS($\overset{ˆ}{\alpha }$)	0.23197^{9}^	0.22411^{6}^	0.2163^{5}^	0.20915^{4}^	0.25361^{14}^	0.26332^{15}^	0.20641^{3}^	0.24251^{12}^	0.1699^{2}^	0.22454^{7}^	0.24592^{13}^	0.05599^{1}^	0.23785^{10}^	0.22895^{8}^	0.2419^{11}^
	BIAS($\overset{ˆ}{\beta }$)	0.04172^{6}^	0.04261^{7}^	0.04771^{11}^	0.03573^{1}^	0.0477^{10}^	0.04295^{8}^	0.04067^{4}^	0.04836^{12}^	0.05082^{13}^	0.05146^{14}^	0.06494^{15}^	0.03712^{2}^	0.04561^{9}^	0.03745^{3}^	0.04167^{5}^
	MSE($\overset{ˆ}{\alpha }$)	0.07843^{8}^	0.07877^{9}^	0.06961^{5}^	0.06513^{4}^	0.08817^{13}^	0.10003^{15}^	0.0616^{3}^	0.08054^{11}^	0.05083^{2}^	0.07367^{7}^	0.09067^{14}^	0.0107^{1}^	0.07952^{10}^	0.07348^{6}^	0.08393^{12}^
	MSE($\overset{ˆ}{\beta }$)	0.00286^{7}^	0.00271^{5}^	0.00373^{12}^	0.0022^{2}^	0.0034^{10}^	0.0028^{6}^	0.00261^{4}^	0.00364^{11}^	0.00386^{13}^	0.00387^{14}^	0.00643^{15}^	0.00211^{1}^	0.00322^{9}^	0.00232^{3}^	0.00316^{8}^
	MRE($\overset{ˆ}{\alpha }$)	0.38662^{9}^	0.37351^{6}^	0.36049^{5}^	0.34859^{4}^	0.42268^{14}^	0.43887^{15}^	0.34402^{3}^	0.40419^{12}^	0.28316^{2}^	0.37424^{7}^	0.40987^{13}^	0.09331^{1}^	0.39642^{10}^	0.38158^{8}^	0.40316^{11}^
	MRE($\overset{ˆ}{\beta }$)	0.04635^{6}^	0.04734^{7}^	0.05301^{11}^	0.0397^{1}^	0.05299^{10}^	0.04772^{8}^	0.04519^{4}^	0.05374^{12}^	0.05647^{13}^	0.05718^{14}^	0.07216^{15}^	0.04125^{2}^	0.05067^{9}^	0.04161^{3}^	0.0463^{5}^
	*D*_*abs*_	0.01905^{3}^	0.02013^{5}^	0.02382^{10}^	0.0189^{2}^	0.02169^{9}^	0.02037^{7}^	0.01914^{4}^	0.02082^{8}^	0.02667^{15}^	0.02383^{11}^	0.02484^{14}^	0.02018^{6}^	0.02466^{13}^	0.01858^{1}^	0.02391^{12}^
	*D*_*max*_	0.03089^{5}^	0.03136^{6}^	0.03708^{11}^	0.02939^{1}^	0.03477^{9}^	0.03282^{7}^	0.0304^{4}^	0.03349^{8}^	0.04026^{15}^	0.03723^{12}^	0.03979^{14}^	0.02956^{2}^	0.0376^{13}^	0.02958^{3}^	0.03661^{10}^
	ASAE	0.05559^{5}^	0.02696^{6}^	0.18219^{11}^	0.01703^{1}^	0.18528^{9}^	0.1039^{7}^	0.07471^{4}^	0.07087^{8}^	0.01704^{15}^	0.04046^{12}^	0.14855^{14}^	0.03268^{2}^	0.04903^{13}^	0.02494^{3}^	0.01779^{10}^
	∑*Ranks*	62^{6}^	56^{5}^	84^{9}^	20^{1}^	104^{14}^	93^{11.5}^	40^{4}^	96^{13}^	77^{7.5}^	93^{11.5}^	126^{15}^	22^{2}^	91^{10}^	39^{3}^	77^{7.5}^
200	BIAS($\overset{ˆ}{\alpha }$)	0.21403^{9}^	0.20531^{5}^	0.21266^{8}^	0.19135^{3}^	0.21253^{7}^	0.25804^{15}^	0.20469^{4}^	0.23643^{13}^	0.16802^{2}^	0.21669^{10}^	0.24445^{14}^	0.04482^{1}^	0.23006^{11}^	0.21012^{6}^	0.23214^{12}^
	BIAS($\overset{ˆ}{\beta }$)	0.03656^{6}^	0.03725^{7}^	0.04039^{12}^	0.0313^{2}^	0.03591^{4}^	0.03651^{5}^	0.03863^{8}^	0.04733^{14}^	0.03906^{11}^	0.03901^{10}^	0.05148^{15}^	0.02853^{1}^	0.04513^{13}^	0.03526^{3}^	0.03893^{9}^
	MSE($\overset{ˆ}{\alpha }$)	0.06677^{7}^	0.05933^{4}^	0.06802^{8}^	0.05837^{3}^	0.07217^{10}^	0.0941^{15}^	0.06156^{5}^	0.07691^{11}^	0.05005^{2}^	0.06654^{6}^	0.08819^{14}^	0.00475^{1}^	0.07825^{13}^	0.07212^{9}^	0.07811^{12}^
	MSE($\overset{ˆ}{\beta }$)	0.00211^{6}^	0.00204^{3.5}^	0.00261^{12}^	0.0017^{2}^	0.00215^{7}^	0.00204^{3.5}^	0.00232^{10}^	0.00339^{14}^	0.00224^{8}^	0.0023^{9}^	0.00462^{15}^	0.00136^{1}^	0.00295^{13}^	0.00206^{5}^	0.00239^{11}^
	MRE($\overset{ˆ}{\alpha }$)	0.35672^{9}^	0.34219^{5}^	0.35444^{8}^	0.31892^{3}^	0.35422^{7}^	0.43007^{15}^	0.34116^{4}^	0.39405^{13}^	0.28003^{2}^	0.36115^{10}^	0.40742^{14}^	0.0747^{1}^	0.38343^{11}^	0.3502^{6}^	0.3869^{12}^
	MRE($\overset{ˆ}{\beta }$)	0.04063^{6}^	0.04138^{7}^	0.04487^{12}^	0.03478^{2}^	0.0399^{4}^	0.04057^{5}^	0.04292^{8}^	0.05258^{14}^	0.0434^{11}^	0.04335^{10}^	0.0572^{15}^	0.03171^{1}^	0.05014^{13}^	0.03918^{3}^	0.04325^{9}^
	*D*_*abs*_	0.01584^{1}^	0.01711^{5}^	0.02078^{13}^	0.0168^{4}^	0.01626^{3}^	0.01722^{6}^	0.01829^{7}^	0.01986^{11}^	0.01921^{9}^	0.01953^{10}^	0.02013^{12}^	0.01604^{2}^	0.02199^{14}^	0.01834^{8}^	0.02234^{15}^
	*D*_*max*_	0.02565^{2}^	0.02677^{5}^	0.03223^{12}^	0.02616^{3}^	0.02669^{4}^	0.02857^{6}^	0.02876^{7}^	0.0321^{11}^	0.02972^{9}^	0.03051^{10}^	0.03286^{13}^	0.02334^{1}^	0.03411^{14}^	0.02901^{8}^	0.03424^{15}^
	ASAE	0.0478^{2}^	0.01476^{5}^	0.01045^{12}^	0.01034^{3}^	0.0589^{4}^	0.09951^{6}^	0.01501^{7}^	0.0141^{11}^	0.01425^{9}^	0.01768^{10}^	0.02022^{13}^	0.01206^{1}^	0.02686^{14}^	0.01183^{8}^	0.01604^{15}^
	∑*Ranks*	59^{5}^	48.5^{3}^	87^{11}^	23^{2}^	60^{6.5}^	85.5^{10}^	61^{8}^	106^{13}^	60^{6.5}^	85^{9}^	123^{15}^	13^{1}^	114^{14}^	51^{4}^	104^{12}^
250	BIAS($\overset{ˆ}{\alpha }$)	0.2011^{8}^	0.19846^{7}^	0.20509^{9}^	0.17614^{3}^	0.19138^{5}^	0.22775^{15}^	0.19687^{6}^	0.22096^{14}^	0.16308^{2}^	0.21526^{11}^	0.20696^{10}^	0.04048^{1}^	0.22001^{12}^	0.189^{4}^	0.22014^{13}^
	BIAS($\overset{ˆ}{\beta }$)	0.03085^{5}^	0.034^{9}^	0.02954^{2}^	0.02988^{3}^	0.03184^{6}^	0.0321^{7}^	0.0348^{10}^	0.03591^{11}^	0.03058^{4}^	0.03774^{12}^	0.04555^{15}^	0.02632^{1}^	0.04293^{14}^	0.03261^{8}^	0.03835^{13}^
	MSE($\overset{ˆ}{\alpha }$)	0.05927^{8}^	0.05742^{7}^	0.06105^{9}^	0.04577^{2}^	0.05613^{6}^	0.07602^{15}^	0.05534^{4}^	0.07333^{14}^	0.04874^{3}^	0.06502^{11}^	0.0631^{10}^	0.00452^{1}^	0.07298^{13}^	0.05553^{5}^	0.06882^{12}^
	MSE($\overset{ˆ}{\beta }$)	0.00155^{5}^	0.0019^{9.5}^	0.00134^{2}^	0.00139^{3}^	0.00168^{7}^	0.00144^{4}^	0.0019^{9.5}^	0.00193^{11}^	0.0016^{6}^	0.00206^{12}^	0.0033^{15}^	0.00106^{1}^	0.00269^{14}^	0.00179^{8}^	0.00212^{13}^
	MRE($\overset{ˆ}{\alpha }$)	0.33517^{8}^	0.33077^{7}^	0.34181^{9}^	0.29357^{3}^	0.31896^{5}^	0.37959^{15}^	0.32811^{6}^	0.36827^{14}^	0.2718^{2}^	0.35877^{11}^	0.34493^{10}^	0.06747^{1}^	0.36668^{12}^	0.315^{4}^	0.3669^{13}^
	MRE($\overset{ˆ}{\beta }$)	0.03428^{5}^	0.03778^{9}^	0.03282^{2}^	0.0332^{3}^	0.03538^{6}^	0.03567^{7}^	0.03867^{10}^	0.0399^{11}^	0.03398^{4}^	0.04194^{12}^	0.05061^{15}^	0.02925^{1}^	0.0477^{14}^	0.03624^{8}^	0.04261^{13}^
	*D*_*abs*_	0.0155^{4}^	0.01659^{9}^	0.0167^{10}^	0.01462^{2}^	0.01428^{1}^	0.01704^{12}^	0.01648^{8}^	0.01697^{11}^	0.01591^{6}^	0.01641^{7}^	0.0194^{13}^	0.01501^{3}^	0.02156^{15}^	0.01571^{5}^	0.02006^{14}^
	*D*_*max*_	0.02498^{6}^	0.02631^{7}^	0.02651^{8}^	0.02329^{3}^	0.02298^{2}^	0.02695^{11}^	0.02665^{9.5}^	0.02744^{12}^	0.02467^{4}^	0.02665^{9.5}^	0.03098^{13}^	0.02164^{1}^	0.03342^{15}^	0.02481^{5}^	0.0311^{14}^
	ASAE	0.02085^{6}^	0.0086^{7}^	0.00937^{8}^	0.00931^{3}^	0.05698^{2}^	0.01928^{11}^	0.01358^{9.5}^	0.01251^{12}^	0.01231^{4}^	0.01102^{9.5}^	0.01234^{13}^	0.0105^{1}^	0.01309^{15}^	0.01116^{5}^	0.01392^{14}^
	∑*Ranks*	63^{7}^	65.5^{8}^	54^{6}^	24^{2}^	53^{4.5}^	99^{11}^	74^{9}^	107^{12}^	38^{3}^	90.5^{10}^	109^{13}^	14^{1}^	119^{15}^	53^{4.5}^	117^{14}^
400	BIAS($\overset{ˆ}{\alpha }$)	0.15641^{2}^	0.16945^{7}^	0.17594^{8}^	0.15858^{3}^	0.17829^{9}^	0.21761^{15}^	0.1783^{10}^	0.16346^{5}^	0.16087^{4}^	0.19154^{11}^	0.20402^{13}^	0.03705^{1}^	0.19226^{12}^	0.16604^{6}^	0.21282^{14}^
	BIAS($\overset{ˆ}{\beta }$)	0.02464^{2}^	0.02781^{11}^	0.02607^{4}^	0.02763^{10}^	0.02742^{7}^	0.02762^{9}^	0.02558^{3}^	0.02679^{5}^	0.02741^{6}^	0.02759^{8}^	0.03648^{14}^	0.02086^{1}^	0.03658^{15}^	0.028^{12}^	0.03512^{13}^
	MSE($\overset{ˆ}{\alpha }$)	0.03675^{2}^	0.042^{5}^	0.04728^{9}^	0.04076^{3}^	0.04762^{10}^	0.06681^{15}^	0.04634^{8}^	0.04227^{6}^	0.04169^{4}^	0.05601^{12}^	0.06175^{13}^	0.00446^{1}^	0.05554^{11}^	0.0433^{7}^	0.06369^{14}^
	MSE($\overset{ˆ}{\beta }$)	0.00093^{2}^	0.00118^{9}^	0.00111^{5}^	0.00115^{7.5}^	0.00103^{4}^	0.0012^{11}^	0.00102^{3}^	0.00113^{6}^	0.00121^{12}^	0.00115^{7.5}^	0.002^{15}^	0.00071^{1}^	0.00187^{13}^	0.00119^{10}^	0.00199^{14}^
	MRE($\overset{ˆ}{\alpha }$)	0.26068^{2}^	0.28242^{7}^	0.29323^{8}^	0.2643^{3}^	0.29714^{9}^	0.36268^{15}^	0.29717^{10}^	0.27243^{5}^	0.26811^{4}^	0.31923^{11}^	0.34003^{13}^	0.06175^{1}^	0.32043^{12}^	0.27674^{6}^	0.3547^{14}^
	MRE($\overset{ˆ}{\beta }$)	0.02737^{2}^	0.03089^{11}^	0.02897^{4}^	0.03069^{9.5}^	0.03047^{7}^	0.03069^{9.5}^	0.02842^{3}^	0.02976^{5}^	0.03046^{6}^	0.03065^{8}^	0.04054^{14}^	0.02318^{1}^	0.04065^{15}^	0.03111^{12}^	0.03902^{13}^
	*D*_*abs*_	0.01219^{5}^	0.0124^{7}^	0.01208^{3}^	0.01238^{6}^	0.01254^{8}^	0.01207^{2}^	0.01372^{11}^	0.01299^{9}^	0.01365^{10}^	0.01453^{12}^	0.01464^{13}^	0.01171^{1}^	0.01549^{14}^	0.0121^{4}^	0.01734^{15}^
	*D*_*max*_	0.01948^{2}^	0.02002^{6}^	0.01973^{4}^	0.02^{5}^	0.0207^{9}^	0.02067^{8}^	0.02204^{11}^	0.02051^{7}^	0.02151^{10}^	0.0228^{12}^	0.02414^{13}^	0.01713^{1}^	0.02508^{14}^	0.0196^{3}^	0.02766^{15}^
	ASAE	0.00701^{2}^	0.00625^{6}^	0.0066^{4}^	0.0071^{5}^	0.03263^{9}^	0.00638^{8}^	0.00672^{11}^	0.00727^{7}^	0.00904^{10}^	0.00887^{12}^	0.00922^{13}^	0.00758^{1}^	0.01038^{14}^	0.00791^{3}^	0.00965^{15}^
	∑*Ranks*	24^{2}^	64^{7}^	48^{3}^	53^{4}^	78^{10}^	86.5^{11}^	63^{6}^	55^{5}^	67^{8}^	91.5^{12}^	120^{13.5}^	16^{1}^	120^{13.5}^	69^{9}^	125^{15}^

**Table 6:** Numerical values of simulation measures for *α* = 1.0, *β* = 1.0.

n	Est.	MLE	ADE	CVME	MPSE	OLSE	RTADE	WLSE	LTADE	MSADE	MSALDE	ADSOE	KE	MSSD	MSSLD	MSLND
25	BIAS($\overset{ˆ}{\alpha }$)	0.52078^{14}^	0.54253^{15}^	0.50631^{11}^	0.38602^{3}^	0.48702^{9}^	0.46999^{7}^	0.46826^{6}^	0.50964^{12}^	0.33283^{2}^	0.4833^{8}^	0.51827^{13}^	0.10304^{1}^	0.43317^{4}^	0.44202^{5}^	0.49077^{10}^
	BIAS($\overset{ˆ}{\beta }$)	0.11947^{7}^	0.11129^{2}^	0.11421^{3}^	0.10391^{1}^	0.1176^{5}^	0.12717^{11}^	0.12599^{10}^	0.11814^{6}^	0.13082^{12}^	0.13296^{13}^	0.13381^{14}^	0.11498^{4}^	0.12131^{9}^	0.12047^{8}^	0.13909^{15}^
	MSE($\overset{ˆ}{\alpha }$)	0.3483^{12}^	0.36534^{15}^	0.33431^{11}^	0.21002^{3}^	0.31489^{8}^	0.30909^{7}^	0.29118^{6}^	0.35203^{13}^	0.20594^{2}^	0.32877^{10}^	0.35523^{14}^	0.03924^{1}^	0.259^{4}^	0.27648^{5}^	0.32549^{9}^
	MSE($\overset{ˆ}{\beta }$)	0.02264^{5}^	0.02378^{7}^	0.0217^{3}^	0.01789^{1}^	0.02324^{6}^	0.02498^{8}^	0.02706^{11}^	0.02808^{13}^	0.02647^{10}^	0.02801^{12}^	0.03453^{15}^	0.02065^{2}^	0.02627^{9}^	0.02254^{4}^	0.03277^{14}^
	MRE($\overset{ˆ}{\alpha }$)	0.52078^{14}^	0.54253^{15}^	0.50631^{11}^	0.38602^{3}^	0.48702^{9}^	0.46999^{7}^	0.46826^{6}^	0.50964^{12}^	0.33283^{2}^	0.4833^{8}^	0.51827^{13}^	0.10304^{1}^	0.43317^{4}^	0.44202^{5}^	0.49077^{10}^
	MRE($\overset{ˆ}{\beta }$)	0.11947^{7}^	0.11129^{2}^	0.11421^{3}^	0.10391^{1}^	0.1176^{5}^	0.12717^{11}^	0.12599^{10}^	0.11814^{6}^	0.13082^{12}^	0.13296^{13}^	0.13381^{14}^	0.11498^{4}^	0.12131^{9}^	0.12047^{8}^	0.13909^{15}^
	*D*_*abs*_	0.04558^{2}^	0.04706^{6}^	0.04206^{1}^	0.04682^{5}^	0.04648^{4}^	0.0502^{7}^	0.0525^{9}^	0.04632^{3}^	0.05955^{14}^	0.05317^{10}^	0.05507^{12}^	0.05592^{13}^	0.05368^{11}^	0.05105^{8}^	0.0615^{15}^
	*D*_*max*_	0.07229^{4}^	0.07363^{6}^	0.0675^{1}^	0.0689^{2}^	0.0721^{3}^	0.07875^{8}^	0.08094^{11}^	0.07311^{5}^	0.08744^{14}^	0.0802^{10}^	0.08506^{13}^	0.08169^{12}^	0.07912^{9}^	0.07784^{7}^	0.0921^{15}^
	ASAE	0.2296^{4}^	0.25914^{6}^	0.27367^{1}^	0.03948^{2}^	0.0729^{3}^	0.13779^{8}^	0.08705^{11}^	0.37581^{5}^	0.34453^{14}^	0.18814^{10}^	0.33089^{13}^	0.0451^{12}^	0.10888^{9}^	0.15399^{7}^	0.15488^{15}^
	∑*Ranks*	75^{9}^	79^{10}^	56^{4}^	20^{1}^	52^{3}^	72^{7}^	73^{8}^	85^{12}^	82^{11}^	93^{13}^	121^{15}^	40^{2}^	64^{6}^	57^{5}^	111^{14}^
75	BIAS($\overset{ˆ}{\alpha }$)	0.44608^{13}^	0.42733^{12}^	0.39616^{7}^	0.37349^{4}^	0.42232^{11}^	0.39284^{6}^	0.40224^{8}^	0.4602^{14}^	0.28109^{2}^	0.40391^{9}^	0.47615^{15}^	0.07339^{1}^	0.41072^{10}^	0.36937^{3}^	0.38861^{5}^
	BIAS($\overset{ˆ}{\beta }$)	0.07394^{8}^	0.07699^{9}^	0.06303^{2}^	0.06895^{6}^	0.07961^{11}^	0.0783^{10}^	0.06472^{3}^	0.08438^{12}^	0.07073^{7}^	0.06789^{5}^	0.10785^{15}^	0.054^{1}^	0.08519^{13}^	0.06527^{4}^	0.096^{14}^
	MSE($\overset{ˆ}{\alpha }$)	0.25299^{12}^	0.26087^{13}^	0.2219^{5}^	0.20057^{3}^	0.24032^{10}^	0.2249^{7}^	0.22799^{8}^	0.29141^{14}^	0.15486^{2}^	0.23333^{9}^	0.29734^{15}^	0.02434^{1}^	0.24195^{11}^	0.20501^{4}^	0.22338^{6}^
	MSE($\overset{ˆ}{\beta }$)	0.00869^{9}^	0.00952^{10}^	0.00691^{3}^	0.00725^{4}^	0.01003^{11}^	0.00862^{8}^	0.00655^{2}^	0.0114^{13}^	0.00802^{7}^	0.00758^{5}^	0.02128^{15}^	0.0049^{1}^	0.01127^{12}^	0.00761^{6}^	0.01461^{14}^
	MRE($\overset{ˆ}{\alpha }$)	0.44608^{13}^	0.42733^{12}^	0.39616^{7}^	0.37349^{4}^	0.42232^{11}^	0.39284^{6}^	0.40224^{8}^	0.4602^{14}^	0.28109^{2}^	0.40391^{9}^	0.47615^{15}^	0.07339^{1}^	0.41072^{10}^	0.36937^{3}^	0.38861^{5}^
	MRE($\overset{ˆ}{\beta }$)	0.07394^{8}^	0.07699^{9}^	0.06303^{2}^	0.06895^{6}^	0.07961^{11}^	0.0783^{10}^	0.06472^{3}^	0.08438^{12}^	0.07073^{7}^	0.06789^{5}^	0.10785^{15}^	0.054^{1}^	0.08519^{13}^	0.06527^{4}^	0.096^{14}^
	*D*_*abs*_	0.02653^{2}^	0.03214^{11}^	0.02677^{3}^	0.03044^{8}^	0.0286^{6}^	0.02843^{5}^	0.02767^{4}^	0.03229^{12}^	0.03211^{10}^	0.0321^{9}^	0.03508^{13}^	0.02477^{1}^	0.03704^{15}^	0.02988^{7}^	0.03513^{14}^
	*D*_*max*_	0.04345^{2}^	0.05118^{11}^	0.04391^{3}^	0.04779^{8}^	0.04609^{6}^	0.04598^{5}^	0.04434^{4}^	0.05264^{12}^	0.0488^{9}^	0.05^{10}^	0.05778^{14}^	0.0365^{1}^	0.05841^{15}^	0.04692^{7}^	0.05521^{13}^
	ASAE	0.01904^{2}^	0.06729^{11}^	0.0264^{3}^	0.03672^{8}^	0.04591^{6}^	0.02832^{5}^	0.0345^{4}^	0.1391^{12}^	0.02416^{9}^	0.02467^{10}^	0.02747^{14}^	0.04036^{1}^	0.03014^{15}^	0.02355^{7}^	0.04491^{13}^
	∑*Ranks*	68^{9}^	101^{12}^	37^{2}^	53^{6}^	90^{10}^	64^{7}^	49^{4.5}^	118^{14}^	49^{4.5}^	65^{8}^	123^{15}^	19^{1}^	107^{13}^	40^{3}^	97^{11}^
150	BIAS($\overset{ˆ}{\alpha }$)	0.40295^{15}^	0.33796^{4}^	0.34545^{6}^	0.36643^{10}^	0.34484^{5}^	0.35479^{8}^	0.39052^{13}^	0.37061^{11}^	0.24999^{2}^	0.37639^{12}^	0.39774^{14}^	0.04863^{1}^	0.35003^{7}^	0.35608^{9}^	0.3348^{3}^
	BIAS($\overset{ˆ}{\beta }$)	0.05299^{6}^	0.05559^{9}^	0.04853^{2}^	0.05434^{7}^	0.06426^{14}^	0.05109^{4}^	0.04949^{3}^	0.06159^{13}^	0.05464^{8}^	0.05969^{11}^	0.0956^{15}^	0.03952^{1}^	0.06104^{12}^	0.05245^{5}^	0.05923^{10}^
	MSE($\overset{ˆ}{\alpha }$)	0.21607^{15}^	0.16403^{3}^	0.1715^{5}^	0.19267^{10}^	0.1839^{8}^	0.18247^{7}^	0.21341^{13}^	0.2022^{11}^	0.11391^{2}^	0.21373^{14}^	0.21286^{12}^	0.01029^{1}^	0.18089^{6}^	0.19064^{9}^	0.16584^{4}^
	MSE($\overset{ˆ}{\beta }$)	0.00447^{5}^	0.0045^{6}^	0.00381^{3}^	0.00441^{4}^	0.00666^{14}^	0.00451^{7}^	0.0038^{2}^	0.00602^{13}^	0.00497^{9}^	0.00535^{10}^	0.01457^{15}^	0.00243^{1}^	0.0059^{12}^	0.00471^{8}^	0.00577^{11}^
	MRE($\overset{ˆ}{\alpha }$)	0.40295^{15}^	0.33796^{4}^	0.34545^{6}^	0.36643^{10}^	0.34484^{5}^	0.35479^{8}^	0.39052^{13}^	0.37061^{11}^	0.24999^{2}^	0.37639^{12}^	0.39774^{14}^	0.04863^{1}^	0.35003^{7}^	0.35608^{9}^	0.3348^{3}^
	MRE($\overset{ˆ}{\beta }$)	0.05299^{6}^	0.05559^{9}^	0.04853^{2}^	0.05434^{7}^	0.06426^{14}^	0.05109^{4}^	0.04949^{3}^	0.06159^{13}^	0.05464^{8}^	0.05969^{11}^	0.0956^{15}^	0.03952^{1}^	0.06104^{12}^	0.05245^{5}^	0.05923^{10}^
	*D*_*abs*_	0.02078^{5}^	0.0216^{6}^	0.01768^{1}^	0.02332^{11}^	0.02162^{7}^	0.02005^{3}^	0.02006^{4}^	0.02269^{9}^	0.0228^{10}^	0.02267^{8}^	0.0285^{15}^	0.01935^{2}^	0.02704^{14}^	0.0241^{13}^	0.02366^{12}^
	*D*_*max*_	0.03437^{5}^	0.03558^{7}^	0.0295^{2}^	0.03683^{10}^	0.03531^{6}^	0.03331^{4}^	0.03295^{3}^	0.03731^{11}^	0.03586^{8}^	0.03655^{9}^	0.04727^{15}^	0.02853^{1}^	0.04267^{14}^	0.03794^{13}^	0.03757^{12}^
	ASAE	0.0129^{5}^	0.01195^{7}^	0.01178^{2}^	0.01253^{10}^	0.0382^{6}^	0.01216^{4}^	0.01231^{3}^	0.01398^{11}^	0.01572^{8}^	0.01523^{9}^	0.02119^{15}^	0.01527^{1}^	0.0176^{14}^	0.01426^{13}^	0.01882^{12}^
	∑*Ranks*	78^{8.5}^	50^{4}^	28^{2}^	74^{7}^	88^{11}^	48^{3}^	58^{5}^	99^{14}^	60^{6}^	96^{12.5}^	129^{15}^	19^{1}^	96^{12.5}^	79^{10}^	78^{8.5}^
200	BIAS($\overset{ˆ}{\alpha }$)	0.33759^{12}^	0.27964^{4}^	0.27595^{3}^	0.34172^{14}^	0.2797^{5}^	0.31319^{9}^	0.29302^{6}^	0.32758^{11}^	0.23912^{2}^	0.31478^{10}^	0.36327^{15}^	0.03839^{1}^	0.29503^{7}^	0.33861^{13}^	0.30181^{8}^
	BIAS($\overset{ˆ}{\beta }$)	0.04554^{9}^	0.04026^{5}^	0.04403^{8}^	0.0392^{4}^	0.03842^{2}^	0.03887^{3}^	0.04138^{6}^	0.05551^{14}^	0.04942^{11}^	0.04632^{10}^	0.06913^{15}^	0.03156^{1}^	0.05134^{12}^	0.04347^{7}^	0.05187^{13}^
	MSE($\overset{ˆ}{\alpha }$)	0.16472^{13}^	0.12135^{5}^	0.12014^{4}^	0.19021^{14}^	0.11589^{3}^	0.137^{7}^	0.14088^{8}^	0.164^{12}^	0.10542^{2}^	0.15524^{10}^	0.1961^{15}^	0.00504^{1}^	0.12825^{6}^	0.16233^{11}^	0.15013^{9}^
	MSE($\overset{ˆ}{\beta }$)	0.00318^{9}^	0.00241^{4}^	0.003^{7}^	0.00236^{3}^	0.00246^{5}^	0.00226^{2}^	0.00258^{6}^	0.00458^{14}^	0.00395^{11}^	0.0033^{10}^	0.00725^{15}^	0.00158^{1}^	0.00416^{12}^	0.00302^{8}^	0.00433^{13}^
	MRE($\overset{ˆ}{\alpha }$)	0.33759^{12}^	0.27964^{4}^	0.27595^{3}^	0.34172^{14}^	0.2797^{5}^	0.31319^{9}^	0.29302^{6}^	0.32758^{11}^	0.23912^{2}^	0.31478^{10}^	0.36327^{15}^	0.03839^{1}^	0.29503^{7}^	0.33861^{13}^	0.30181^{8}^
	MRE($\overset{ˆ}{\beta }$)	0.04554^{9}^	0.04026^{5}^	0.04403^{8}^	0.0392^{4}^	0.03842^{2}^	0.03887^{3}^	0.04138^{6}^	0.05551^{14}^	0.04942^{11}^	0.04632^{10}^	0.06913^{15}^	0.03156^{1}^	0.05134^{12}^	0.04347^{7}^	0.05187^{13}^
	*D*_*abs*_	0.01711^{10}^	0.01455^{1}^	0.01707^{8}^	0.01509^{4}^	0.01536^{5}^	0.01494^{2}^	0.01625^{7}^	0.01607^{6}^	0.02008^{13}^	0.0171^{9}^	0.01824^{12}^	0.015^{3}^	0.02299^{15}^	0.01734^{11}^	0.02241^{14}^
	*D*_*max*_	0.02842^{10}^	0.02416^{2}^	0.02771^{8}^	0.02513^{4}^	0.02483^{3}^	0.0252^{5}^	0.02708^{7}^	0.02698^{6}^	0.0317^{13}^	0.02816^{9}^	0.03116^{12}^	0.02201^{1}^	0.0358^{15}^	0.02846^{11}^	0.03512^{14}^
	ASAE	0.00893^{10}^	0.00852^{2}^	0.00874^{8}^	0.00884^{4}^	0.00911^{3}^	0.00855^{5}^	0.00848^{7}^	0.00965^{6}^	0.01273^{13}^	0.01056^{9}^	0.01321^{12}^	0.0104^{1}^	0.01418^{15}^	0.01048^{11}^	0.01429^{14}^
	∑*Ranks*	90^{10}^	32^{2}^	53^{5.5}^	66^{7}^	37^{3}^	43^{4}^	53^{5.5}^	96^{12}^	77^{8}^	89^{9}^	127^{15}^	19^{1}^	100^{13}^	91^{11}^	107^{14}^
250	BIAS($\overset{ˆ}{\alpha }$)	0.23809^{3}^	0.25193^{8}^	0.24122^{4}^	0.27257^{11}^	0.25101^{6}^	0.27588^{12}^	0.24576^{5}^	0.25913^{10}^	0.21809^{2}^	0.25894^{9}^	0.3305^{15}^	0.03518^{1}^	0.28407^{13}^	0.25157^{7}^	0.30092^{14}^
	BIAS($\overset{ˆ}{\beta }$)	0.02784^{2}^	0.03448^{7}^	0.03552^{8}^	0.03408^{6}^	0.03685^{11}^	0.03667^{10}^	0.03349^{4}^	0.03251^{3}^	0.03655^{9}^	0.04047^{12}^	0.05717^{15}^	0.02562^{1}^	0.04494^{14}^	0.03395^{5}^	0.04307^{13}^
	MSE($\overset{ˆ}{\alpha }$)	0.09977^{6}^	0.09432^{4}^	0.09365^{3}^	0.11475^{11}^	0.10187^{7}^	0.11674^{13}^	0.10263^{8}^	0.10602^{9}^	0.08524^{2}^	0.1123^{10}^	0.16314^{15}^	0.00381^{1}^	0.11611^{12}^	0.09695^{5}^	0.14452^{14}^
	MSE($\overset{ˆ}{\beta }$)	0.00139^{2}^	0.00197^{7}^	0.00201^{10}^	0.0018^{5}^	0.00199^{8}^	0.00202^{11}^	0.00166^{3}^	0.00176^{4}^	0.002^{9}^	0.0026^{12}^	0.00485^{15}^	0.00097^{1}^	0.00306^{14}^	0.00181^{6}^	0.00305^{13}^
	MRE($\overset{ˆ}{\alpha }$)	0.23809^{3}^	0.25193^{8}^	0.24122^{4}^	0.27257^{11}^	0.25101^{6}^	0.27588^{12}^	0.24576^{5}^	0.25913^{10}^	0.21809^{2}^	0.25894^{9}^	0.3305^{15}^	0.03518^{1}^	0.28407^{13}^	0.25157^{7}^	0.30092^{14}^
	MRE($\overset{ˆ}{\beta }$)	0.02784^{2}^	0.03448^{7}^	0.03552^{8}^	0.03408^{6}^	0.03685^{11}^	0.03667^{10}^	0.03349^{4}^	0.03251^{3}^	0.03655^{9}^	0.04047^{12}^	0.05717^{15}^	0.02562^{1}^	0.04494^{14}^	0.03395^{5}^	0.04307^{13}^
	*D*_*abs*_	0.01306^{7}^	0.01361^{10}^	0.01248^{3}^	0.01322^{9}^	0.01287^{6}^	0.01254^{4}^	0.01213^{2}^	0.01276^{5}^	0.01536^{13}^	0.01436^{11}^	0.01465^{12}^	0.01207^{1}^	0.01686^{14}^	0.01314^{8}^	0.0187^{15}^
	*D*_*max*_	0.02133^{6}^	0.02211^{10}^	0.02069^{3}^	0.02153^{9}^	0.02131^{5}^	0.02146^{7.5}^	0.02014^{2}^	0.02104^{4}^	0.02428^{12}^	0.0235^{11}^	0.02533^{13}^	0.01774^{1}^	0.02719^{14}^	0.02146^{7.5}^	0.0299^{15}^
	ASAE	0.00653^{6}^	0.00665^{10}^	0.00671^{3}^	0.00679^{9}^	0.0063^{5}^	0.00632^{7.5}^	0.00672^{2}^	0.00762^{4}^	0.00911^{12}^	0.00858^{11}^	0.00966^{13}^	0.00773^{1}^	0.01014^{14}^	0.00839^{7.5}^	0.01015^{15}^
	∑*Ranks*	34^{2}^	65^{8}^	48^{4}^	75^{10}^	61^{7}^	81.5^{11}^	39^{3}^	56^{5}^	70^{9}^	97^{12}^	128^{15}^	17^{1}^	122^{13}^	60.5^{6}^	126^{14}^
400	BIAS($\overset{ˆ}{\alpha }$)	0.21375^{9}^	0.19936^{4}^	0.20048^{5}^	0.20663^{6}^	0.21016^{8}^	0.21441^{11}^	0.21422^{10}^	0.19172^{3}^	0.17426^{2}^	0.24006^{13}^	0.29631^{15}^	0.03285^{1}^	0.25044^{14}^	0.20761^{7}^	0.22962^{12}^
	BIAS($\overset{ˆ}{\beta }$)	0.02626^{2}^	0.02957^{7}^	0.03137^{11.5}^	0.02977^{9}^	0.02925^{6}^	0.02788^{4}^	0.02845^{5}^	0.02724^{3}^	0.0318^{13}^	0.03236^{14}^	0.04731^{15}^	0.01936^{1}^	0.03017^{10}^	0.02968^{8}^	0.03137^{11.5}^
	MSE($\overset{ˆ}{\alpha }$)	0.06832^{5}^	0.06147^{4}^	0.07047^{6}^	0.07134^{7}^	0.08476^{12}^	0.07673^{10}^	0.07334^{8}^	0.0583^{2}^	0.0598^{3}^	0.09553^{13}^	0.13923^{15}^	0.00335^{1}^	0.10713^{14}^	0.0738^{9}^	0.08322^{11}^
	MSE($\overset{ˆ}{\beta }$)	0.00104^{2}^	0.0014^{9}^	0.0015^{11}^	0.00151^{12}^	0.00137^{7.5}^	0.00122^{4}^	0.00131^{5}^	0.00111^{3}^	0.00155^{13}^	0.00161^{14}^	0.00383^{15}^	0.00059^{1}^	0.00136^{6}^	0.00137^{7.5}^	0.00149^{10}^
	MRE($\overset{ˆ}{\alpha }$)	0.21375^{9}^	0.19936^{4}^	0.20048^{5}^	0.20663^{6}^	0.21016^{8}^	0.21441^{11}^	0.21422^{10}^	0.19172^{3}^	0.17426^{2}^	0.24006^{13}^	0.29631^{15}^	0.03285^{1}^	0.25044^{14}^	0.20761^{7}^	0.22962^{12}^
	MRE($\overset{ˆ}{\beta }$)	0.02626^{2}^	0.02957^{7}^	0.03137^{11.5}^	0.02977^{9}^	0.02925^{6}^	0.02788^{4}^	0.02845^{5}^	0.02724^{3}^	0.0318^{13}^	0.03236^{14}^	0.04731^{15}^	0.01936^{1}^	0.03017^{10}^	0.02968^{8}^	0.03137^{11.5}^
	*D*_*abs*_	0.00967^{2}^	0.0102^{3}^	0.01025^{4}^	0.01122^{9}^	0.0112^{8}^	0.01045^{5}^	0.01071^{7}^	0.01066^{6}^	0.01341^{14}^	0.01226^{11}^	0.01279^{12}^	0.00909^{1}^	0.01436^{15}^	0.01129^{10}^	0.01309^{13}^
	*D*_*max*_	0.01634^{2}^	0.01722^{4}^	0.01687^{3}^	0.01825^{8}^	0.01837^{9}^	0.01729^{5}^	0.01775^{7}^	0.01736^{6}^	0.02132^{12}^	0.02035^{11}^	0.0223^{14}^	0.01341^{1}^	0.02299^{15}^	0.01843^{10}^	0.02134^{13}^
	ASAE	0.00531^{2}^	0.00534^{4}^	0.00521^{3}^	0.00507^{8}^	0.00529^{9}^	0.00499^{5}^	0.00532^{7}^	0.00549^{6}^	0.00714^{12}^	0.00678^{11}^	0.00832^{14}^	0.00615^{1}^	0.00788^{15}^	0.00619^{10}^	0.00793^{13}^
	∑*Ranks*	38^{3}^	49^{4}^	60^{6}^	68^{8}^	68.5^{9}^	55^{5}^	63^{7}^	37^{2}^	84^{11}^	114^{14}^	131^{15}^	17^{1}^	111^{13}^	76.5^{10}^	108^{12}^

**Table 7:** Numerical values of simulation measures for *α* = 0.75, *β* = 1.5.

n	Est.	MLE	ADE	CVME	MPSE	OLSE	RTADE	WLSE	LTADE	MSADE	MSALDE	ADSOE	KE	MSSD	MSSLD	MSLND
25	BIAS($\overset{ˆ}{\alpha }$)	0.35118^{8}^	0.34745^{6}^	0.37242^{10}^	0.32754^{4}^	0.38354^{13}^	0.3827^{12}^	0.37528^{11}^	0.39594^{15}^	0.27879^{2}^	0.31869^{3}^	0.38772^{14}^	0.12957^{1}^	0.34293^{5}^	0.37154^{9}^	0.34993^{7}^
	BIAS($\overset{ˆ}{\beta }$)	0.15304^{2}^	0.17584^{5}^	0.21042^{13}^	0.17522^{4}^	0.17683^{6}^	0.1873^{10}^	0.19978^{11}^	0.21906^{14}^	0.20022^{12}^	0.18577^{9}^	0.25142^{15}^	0.15135^{1}^	0.18013^{7}^	0.16187^{3}^	0.18511^{8}^
	MSE($\overset{ˆ}{\alpha }$)	0.16603^{7}^	0.16558^{6}^	0.18717^{12}^	0.1535^{4}^	0.187^{11}^	0.19423^{13}^	0.17605^{9}^	0.20005^{14}^	0.1328^{2}^	0.14972^{3}^	0.20037^{15}^	0.03881^{1}^	0.16539^{5}^	0.17633^{10}^	0.16775^{8}^
	MSE($\overset{ˆ}{\beta }$)	0.04394^{2}^	0.04884^{5}^	0.07688^{13}^	0.04847^{4}^	0.05211^{7}^	0.06065^{10}^	0.07066^{12}^	0.08188^{14}^	0.06431^{11}^	0.05931^{8}^	0.11205^{15}^	0.03668^{1}^	0.04952^{6}^	0.04674^{3}^	0.06012^{9}^
	MRE($\overset{ˆ}{\alpha }$)	0.46824^{8}^	0.46327^{6}^	0.49656^{10}^	0.43672^{4}^	0.51139^{13}^	0.51026^{12}^	0.50037^{11}^	0.52792^{15}^	0.37172^{2}^	0.42492^{3}^	0.51696^{14}^	0.17275^{1}^	0.45724^{5}^	0.49538^{9}^	0.46657^{7}^
	MRE($\overset{ˆ}{\beta }$)	0.10203^{2}^	0.11723^{5}^	0.14028^{13}^	0.11681^{4}^	0.11789^{6}^	0.12487^{10}^	0.13319^{11}^	0.14604^{14}^	0.13348^{12}^	0.12385^{9}^	0.16761^{15}^	0.1009^{1}^	0.12008^{7}^	0.10791^{3}^	0.1234^{8}^
	*D*_*abs*_	0.04003^{1}^	0.04938^{8}^	0.04983^{9}^	0.04934^{7}^	0.04555^{2}^	0.04678^{4}^	0.05138^{12}^	0.04601^{3}^	0.05976^{15}^	0.05074^{10}^	0.05463^{13}^	0.04914^{6}^	0.05111^{11}^	0.04813^{5}^	0.05946^{14}^
	*D*_*max*_	0.06318^{1}^	0.07618^{9}^	0.07938^{11}^	0.07326^{5}^	0.07098^{2}^	0.07406^{6}^	0.07946^{12}^	0.07476^{7}^	0.08776^{15}^	0.07518^{8}^	0.08682^{14}^	0.07145^{3}^	0.07671^{10}^	0.07199^{4}^	0.0867^{13}^
	ASAE	0.04086^{1}^	0.09485^{9}^	0.1717^{11}^	0.0408^{5}^	0.08148^{2}^	0.19729^{6}^	0.06632^{12}^	0.06186^{7}^	0.1417^{15}^	0.0763^{8}^	0.11535^{14}^	0.04607^{3}^	0.10825^{10}^	0.10004^{4}^	0.1193^{13}^
	∑*Ranks*	33^{2}^	58^{5}^	105^{14}^	37^{3}^	67^{8}^	92^{11}^	94^{12}^	100^{13}^	84^{9}^	59^{6}^	126^{15}^	18^{1}^	66^{7}^	55^{4}^	86^{10}^
75	BIAS($\overset{ˆ}{\alpha }$)	0.31561^{6}^	0.34088^{13}^	0.34779^{14}^	0.30034^{4}^	0.31872^{8}^	0.32522^{10}^	0.31775^{7}^	0.34007^{12}^	0.2562^{3}^	0.25556^{2}^	0.34863^{15}^	0.10889^{1}^	0.32459^{9}^	0.33471^{11}^	0.31241^{5}^
	BIAS($\overset{ˆ}{\beta }$)	0.10799^{3}^	0.11645^{7}^	0.14194^{14}^	0.11209^{4}^	0.12847^{8}^	0.13027^{10}^	0.11338^{5}^	0.137^{11}^	0.14171^{13}^	0.11428^{6}^	0.18208^{15}^	0.09256^{1}^	0.12953^{9}^	0.10298^{2}^	0.1402^{12}^
	MSE($\overset{ˆ}{\alpha }$)	0.14459^{8}^	0.15276^{12}^	0.15964^{14}^	0.1245^{4}^	0.14558^{9}^	0.13944^{5}^	0.14132^{7}^	0.15566^{13}^	0.11053^{3}^	0.09887^{2}^	0.16694^{15}^	0.03212^{1}^	0.14805^{10}^	0.15152^{11}^	0.1412^{6}^
	MSE($\overset{ˆ}{\beta }$)	0.01994^{6}^	0.01908^{5}^	0.03047^{12}^	0.01871^{4}^	0.02492^{8}^	0.02788^{10}^	0.02181^{7}^	0.03448^{14}^	0.02899^{11}^	0.01864^{3}^	0.06105^{15}^	0.01283^{1}^	0.02704^{9}^	0.01821^{2}^	0.03291^{13}^
	MRE($\overset{ˆ}{\alpha }$)	0.42081^{6}^	0.45451^{13}^	0.46372^{14}^	0.40045^{4}^	0.42496^{8}^	0.43363^{10}^	0.42367^{7}^	0.45342^{12}^	0.3416^{3}^	0.34075^{2}^	0.46484^{15}^	0.14519^{1}^	0.43279^{9}^	0.44627^{11}^	0.41655^{5}^
	MRE($\overset{ˆ}{\beta }$)	0.07199^{3}^	0.07763^{7}^	0.09462^{14}^	0.07473^{4}^	0.08565^{8}^	0.08685^{10}^	0.07559^{5}^	0.09133^{11}^	0.09448^{13}^	0.07619^{6}^	0.12139^{15}^	0.0617^{1}^	0.08635^{9}^	0.06865^{2}^	0.09347^{12}^
	*D*_*abs*_	0.02594^{1}^	0.0309^{8}^	0.03203^{9}^	0.027^{2}^	0.02891^{5}^	0.02921^{6}^	0.02731^{4}^	0.03565^{12}^	0.03855^{15}^	0.03234^{10}^	0.03601^{14}^	0.02723^{3}^	0.03507^{11}^	0.03077^{7}^	0.03574^{13}^
	*D*_*max*_	0.04231^{2}^	0.0496^{8}^	0.05287^{10}^	0.04307^{3}^	0.04689^{5}^	0.04751^{6}^	0.04475^{4}^	0.05732^{13}^	0.059^{14}^	0.04977^{9}^	0.05964^{15}^	0.04045^{1}^	0.05513^{11}^	0.04879^{7}^	0.05599^{12}^
	ASAE	0.01852^{2}^	0.01847^{8}^	0.05015^{10}^	0.0187^{3}^	0.02749^{5}^	0.06885^{6}^	0.02983^{4}^	0.03242^{13}^	0.0245^{14}^	0.02602^{9}^	0.02845^{15}^	0.02183^{1}^	0.02696^{11}^	0.02134^{7}^	0.05878^{12}^
	∑*Ranks*	37^{3}^	74^{8}^	114^{14}^	32^{2}^	68^{7}^	82^{10}^	57^{5.5}^	110^{13}^	81^{9}^	47^{4}^	129^{15}^	15^{1}^	85^{11}^	57^{5.5}^	92^{12}^
150	BIAS($\overset{ˆ}{\alpha }$)	0.25101^{6}^	0.27215^{10}^	0.28456^{13}^	0.25002^{5}^	0.25407^{7}^	0.30775^{15}^	0.26373^{8}^	0.27692^{12}^	0.2381^{2}^	0.24157^{3}^	0.29549^{14}^	0.09568^{1}^	0.24375^{4}^	0.26707^{9}^	0.27522^{11}^
	BIAS($\overset{ˆ}{\beta }$)	0.08353^{4}^	0.09577^{9}^	0.09822^{10}^	0.08049^{3}^	0.08746^{5}^	0.07731^{2}^	0.09236^{7}^	0.10806^{14}^	0.10333^{11}^	0.08815^{6}^	0.13652^{15}^	0.06708^{1}^	0.10782^{12}^	0.09511^{8}^	0.10796^{13}^
	MSE($\overset{ˆ}{\alpha }$)	0.09587^{7}^	0.10009^{8}^	0.11877^{13}^	0.09145^{5}^	0.0914^{4}^	0.12832^{15}^	0.10246^{10}^	0.10923^{12}^	0.09224^{6}^	0.08791^{2}^	0.1219^{14}^	0.02501^{1}^	0.09119^{3}^	0.10107^{9}^	0.1089^{11}^
	MSE($\overset{ˆ}{\beta }$)	0.01101^{4}^	0.01495^{9}^	0.01681^{12}^	0.01039^{3}^	0.01112^{5}^	0.00916^{2}^	0.01403^{8}^	0.01856^{14}^	0.01517^{10}^	0.01168^{6}^	0.03112^{15}^	0.00703^{1}^	0.01817^{13}^	0.01393^{7}^	0.01661^{11}^
	MRE($\overset{ˆ}{\alpha }$)	0.33468^{6}^	0.36287^{10}^	0.37941^{13}^	0.33336^{5}^	0.33876^{7}^	0.41033^{15}^	0.35164^{8}^	0.36923^{12}^	0.31746^{2}^	0.3221^{3}^	0.39399^{14}^	0.12758^{1}^	0.32499^{4}^	0.35609^{9}^	0.36696^{11}^
	MRE($\overset{ˆ}{\beta }$)	0.05569^{4}^	0.06385^{9}^	0.06548^{10}^	0.05366^{3}^	0.05831^{5}^	0.05154^{2}^	0.06157^{7}^	0.07204^{14}^	0.06889^{11}^	0.05877^{6}^	0.09102^{15}^	0.04472^{1}^	0.07188^{12}^	0.06341^{8}^	0.07197^{13}^
	*D*_*abs*_	0.0183^{1}^	0.02027^{4}^	0.0203^{5}^	0.01973^{2}^	0.02294^{10}^	0.01976^{3}^	0.02156^{8}^	0.02461^{13}^	0.02397^{11}^	0.0214^{7}^	0.02546^{15}^	0.02105^{6}^	0.02496^{14}^	0.02175^{9}^	0.02429^{12}^
	*D*_*max*_	0.02997^{1}^	0.03331^{5}^	0.03424^{6}^	0.03161^{3}^	0.03678^{10}^	0.03264^{4}^	0.03517^{8}^	0.03975^{14}^	0.03841^{11}^	0.03482^{7}^	0.04207^{15}^	0.03142^{2}^	0.03955^{13}^	0.03552^{9}^	0.03939^{12}^
	ASAE	0.01192^{1}^	0.01156^{5}^	0.01196^{6}^	0.01232^{3}^	0.01243^{10}^	0.03145^{4}^	0.01198^{8}^	0.01413^{14}^	0.01532^{11}^	0.0148^{7}^	0.01907^{15}^	0.01492^{2}^	0.01889^{13}^	0.01403^{9}^	0.01659^{12}^
	∑*Ranks*	35^{3}^	65^{6}^	85^{11}^	34^{2}^	59^{5}^	73^{8}^	68^{7}^	113^{14}^	75^{9.5}^	49^{4}^	131^{15}^	24^{1}^	88^{12}^	75^{9.5}^	106^{13}^
200	BIAS($\overset{ˆ}{\alpha }$)	0.22931^{5}^	0.26638^{14}^	0.25912^{12}^	0.24781^{9}^	0.19511^{2}^	0.23438^{7}^	0.23024^{6}^	0.25424^{10}^	0.196^{3}^	0.22816^{4}^	0.29534^{15}^	0.0743^{1}^	0.23725^{8}^	0.26282^{13}^	0.2557^{11}^
	BIAS($\overset{ˆ}{\beta }$)	0.06743^{2}^	0.08075^{11}^	0.07457^{7}^	0.07312^{5}^	0.07773^{9}^	0.06936^{4}^	0.06933^{3}^	0.08758^{13}^	0.07446^{6}^	0.07622^{8}^	0.12706^{15}^	0.0557^{1}^	0.08493^{12}^	0.0796^{10}^	0.08779^{14}^
	MSE($\overset{ˆ}{\alpha }$)	0.07936^{5}^	0.09841^{12}^	0.096^{11}^	0.08964^{9}^	0.05989^{2}^	0.08481^{8}^	0.07779^{4}^	0.09515^{10}^	0.06293^{3}^	0.07956^{6}^	0.11872^{15}^	0.01778^{1}^	0.08335^{7}^	0.10038^{13}^	0.10651^{14}^
	MSE($\overset{ˆ}{\beta }$)	0.00786^{5}^	0.01037^{10}^	0.00828^{6}^	0.0078^{4}^	0.01051^{11}^	0.00736^{2}^	0.00774^{3}^	0.01074^{12}^	0.00876^{7}^	0.009^{9}^	0.02593^{15}^	0.00451^{1}^	0.01189^{13}^	0.00897^{8}^	0.01253^{14}^
	MRE($\overset{ˆ}{\alpha }$)	0.30574^{5}^	0.35517^{14}^	0.34549^{12}^	0.33041^{9}^	0.26015^{2}^	0.31251^{7}^	0.30699^{6}^	0.33899^{10}^	0.26134^{3}^	0.30422^{4}^	0.39378^{15}^	0.09906^{1}^	0.31634^{8}^	0.35043^{13}^	0.34093^{11}^
	MRE($\overset{ˆ}{\beta }$)	0.04496^{2}^	0.05384^{11}^	0.04971^{7}^	0.04874^{5}^	0.05182^{9}^	0.04624^{4}^	0.04622^{3}^	0.05838^{13}^	0.04964^{6}^	0.05081^{8}^	0.0847^{15}^	0.03713^{1}^	0.05662^{12}^	0.05307^{10}^	0.05853^{14}^
	*D*_*abs*_	0.01536^{1}^	0.01775^{7}^	0.01699^{3}^	0.01768^{6}^	0.01706^{4}^	0.01853^{9}^	0.01713^{5}^	0.01788^{8}^	0.02206^{13}^	0.02102^{11}^	0.02182^{12}^	0.0167^{2}^	0.02397^{15}^	0.01878^{10}^	0.02241^{14}^
	*D*_*max*_	0.02534^{2}^	0.02998^{9}^	0.02827^{4}^	0.02856^{6}^	0.0278^{3}^	0.02993^{8}^	0.02831^{5}^	0.02992^{7}^	0.03474^{12}^	0.03328^{11}^	0.03745^{14}^	0.02492^{1}^	0.03749^{15}^	0.03049^{10}^	0.03563^{13}^
	ASAE	0.00999^{2}^	0.00981^{9}^	0.01012^{4}^	0.00994^{6}^	0.01021^{3}^	0.01136^{8}^	0.00994^{5}^	0.01128^{7}^	0.01332^{12}^	0.01263^{11}^	0.01537^{14}^	0.01171^{1}^	0.01555^{15}^	0.01214^{10}^	0.01493^{13}^
	∑*Ranks*	31^{2}^	89^{10}^	67^{8}^	56^{5}^	48^{4}^	57^{6}^	37^{3}^	90^{11}^	65^{7}^	72^{9}^	130^{15}^	18^{1}^	105^{13}^	97^{12}^	118^{14}^
250	BIAS($\overset{ˆ}{\alpha }$)	0.21852^{8}^	0.22606^{12}^	0.19663^{4}^	0.22065^{10}^	0.18709^{3}^	0.21751^{7}^	0.20795^{5}^	0.21868^{9}^	0.18271^{2}^	0.22116^{11}^	0.2882^{15}^	0.07316^{1}^	0.21718^{6}^	0.24406^{14}^	0.23882^{13}^
	BIAS($\overset{ˆ}{\beta }$)	0.06198^{3}^	0.06211^{4}^	0.06805^{8}^	0.06799^{7}^	0.06069^{2}^	0.06591^{6}^	0.06823^{9}^	0.0732^{13}^	0.06251^{5}^	0.07277^{12}^	0.11079^{15}^	0.05107^{1}^	0.07928^{14}^	0.07182^{10}^	0.07243^{11}^
	MSE($\overset{ˆ}{\alpha }$)	0.06782^{5}^	0.0752^{9}^	0.06225^{4}^	0.07651^{11}^	0.05493^{2}^	0.06981^{7}^	0.06843^{6}^	0.07489^{8}^	0.05552^{3}^	0.07614^{10}^	0.11805^{15}^	0.01325^{1}^	0.07707^{12}^	0.09539^{14}^	0.08862^{13}^
	MSE($\overset{ˆ}{\beta }$)	0.00571^{2}^	0.00622^{3.5}^	0.00753^{9}^	0.00718^{8}^	0.00622^{3.5}^	0.00695^{6}^	0.00706^{7}^	0.0087^{13}^	0.00629^{5}^	0.00791^{11}^	0.02044^{15}^	0.00428^{1}^	0.00927^{14}^	0.00818^{12}^	0.00765^{10}^
	MRE($\overset{ˆ}{\alpha }$)	0.29136^{8}^	0.30142^{12}^	0.26217^{4}^	0.2942^{10}^	0.24946^{3}^	0.29001^{7}^	0.27726^{5}^	0.29157^{9}^	0.24361^{2}^	0.29487^{11}^	0.38427^{15}^	0.09754^{1}^	0.28957^{6}^	0.32541^{14}^	0.31842^{13}^
	MRE($\overset{ˆ}{\beta }$)	0.04132^{3}^	0.04141^{4}^	0.04537^{8}^	0.04533^{7}^	0.04046^{2}^	0.04394^{6}^	0.04549^{9}^	0.0488^{13}^	0.04167^{5}^	0.04851^{12}^	0.07386^{15}^	0.03405^{1}^	0.05286^{14}^	0.04788^{10}^	0.04829^{11}^
	*D*_*abs*_	0.0145^{1}^	0.0171^{10}^	0.01642^{6}^	0.01468^{2}^	0.01684^{9}^	0.01497^{3}^	0.01659^{7}^	0.01664^{8}^	0.0158^{5}^	0.01774^{11}^	0.02045^{14}^	0.01536^{4}^	0.02114^{15}^	0.01777^{12}^	0.01874^{13}^
	*D*_*max*_	0.02418^{3}^	0.02776^{10}^	0.02665^{7}^	0.024^{2}^	0.02683^{8}^	0.02478^{4}^	0.0266^{6}^	0.02748^{9}^	0.02548^{5}^	0.02892^{12}^	0.03432^{15}^	0.02316^{1}^	0.03318^{14}^	0.02861^{11}^	0.02985^{13}^
	ASAE	0.00897^{3}^	0.00898^{10}^	0.00891^{7}^	0.00857^{2}^	0.00868^{8}^	0.00865^{4}^	0.0087^{6}^	0.00955^{9}^	0.01207^{5}^	0.01156^{12}^	0.01384^{15}^	0.01024^{1}^	0.01326^{14}^	0.01007^{11}^	0.01211^{13}^
	∑*Ranks*	39^{3}^	71.5^{9}^	55^{6}^	58^{7.5}^	35.5^{2}^	48^{5}^	58^{7.5}^	90^{10}^	44^{4}^	101^{11}^	134^{15}^	21^{1}^	109^{13}^	106^{12}^	110^{14}^
400	BIAS($\overset{ˆ}{\alpha }$)	0.16756^{3}^	0.20431^{8}^	0.19591^{7}^	0.2065^{9}^	0.17541^{5}^	0.21139^{12}^	0.1641^{2}^	0.20897^{10}^	0.17274^{4}^	0.21123^{11}^	0.24913^{15}^	0.06758^{1}^	0.21453^{13}^	0.1947^{6}^	0.21483^{14}^
	BIAS($\overset{ˆ}{\beta }$)	0.0485^{2}^	0.05668^{8}^	0.06028^{9}^	0.05603^{7}^	0.05543^{6}^	0.05112^{5}^	0.04966^{3}^	0.06266^{11}^	0.06208^{10}^	0.0646^{13}^	0.08307^{15}^	0.04371^{1}^	0.0639^{12}^	0.04981^{4}^	0.06775^{14}^
	MSE($\overset{ˆ}{\alpha }$)	0.04743^{2}^	0.0676^{9}^	0.05719^{6}^	0.06965^{11}^	0.05196^{5}^	0.06249^{7}^	0.04779^{3}^	0.06924^{10}^	0.0518^{4}^	0.07239^{12}^	0.0947^{15}^	0.00962^{1}^	0.07597^{13}^	0.06366^{8}^	0.07785^{14}^
	MSE($\overset{ˆ}{\beta }$)	0.00367^{2}^	0.00482^{8}^	0.00564^{9}^	0.00476^{7}^	0.00462^{6}^	0.0045^{5}^	0.00404^{3}^	0.00615^{12}^	0.00621^{13}^	0.0059^{10}^	0.01167^{15}^	0.00295^{1}^	0.00601^{11}^	0.00417^{4}^	0.00723^{14}^
	MRE($\overset{ˆ}{\alpha }$)	0.22341^{3}^	0.27241^{8}^	0.26122^{7}^	0.27533^{9}^	0.23388^{5}^	0.28185^{12}^	0.2188^{2}^	0.27863^{10}^	0.23032^{4}^	0.28164^{11}^	0.33218^{15}^	0.09011^{1}^	0.28604^{13}^	0.25961^{6}^	0.28644^{14}^
	MRE($\overset{ˆ}{\beta }$)	0.03234^{2}^	0.03779^{8}^	0.04019^{9}^	0.03735^{7}^	0.03695^{6}^	0.03408^{5}^	0.03311^{3}^	0.04177^{11}^	0.04139^{10}^	0.04307^{13}^	0.05538^{15}^	0.02914^{1}^	0.0426^{12}^	0.03321^{4}^	0.04517^{14}^
	*D*_*abs*_	0.01224^{2}^	0.01226^{3}^	0.01305^{6}^	0.01257^{4}^	0.01287^{5}^	0.01369^{9}^	0.01069^{1}^	0.01364^{8}^	0.01563^{13}^	0.01516^{12}^	0.01387^{10}^	0.01348^{7}^	0.01822^{15}^	0.01418^{11}^	0.01639^{14}^
	*D*_*max*_	0.01991^{2}^	0.02055^{4}^	0.02164^{7}^	0.02105^{6}^	0.02102^{5}^	0.0226^{8}^	0.0178^{1}^	0.02272^{9}^	0.02486^{13}^	0.02454^{12}^	0.02376^{11}^	0.02029^{3}^	0.02914^{15}^	0.02279^{10}^	0.02675^{14}^
	ASAE	0.00631^{2}^	0.00623^{4}^	0.00668^{7}^	0.00641^{6}^	0.00664^{5}^	0.00609^{8}^	0.00644^{1}^	0.00772^{9}^	0.00876^{13}^	0.00813^{12}^	0.00988^{11}^	0.00707^{3}^	0.00966^{15}^	0.00781^{10}^	0.01024^{14}^
	∑*Ranks*	21^{1}^	58^{5}^	67^{9}^	64^{7.5}^	49^{4}^	64^{7.5}^	23^{2}^	90^{11}^	83^{10}^	105^{12}^	125^{14}^	24^{3}^	117^{13}^	63^{6}^	127^{15}^

**Table 8:** Numerical values of simulation measures for *α* = 2.5, *β* = 3.5.

n	Est.	MLE	ADE	CVME	MPSE	OLSE	RTADE	WLSE	LTADE	MSADE	MSALDE	ADSOE	KE	MSSD	MSSLD	MSLND
25	BIAS($\overset{ˆ}{\alpha }$)	1.48288^{8}^	1.53232^{11}^	1.61765^{13}^	1.3132^{6}^	1.52779^{10}^	1.56818^{12}^	1.52182^{9}^	1.69694^{14}^	0.62456^{2}^	1.0629^{3}^	1.75268^{15}^	0.17627^{1}^	1.22452^{5}^	1.47905^{7}^	1.21583^{4}^
	BIAS($\overset{ˆ}{\beta }$)	0.86594^{10}^	0.89132^{12}^	0.96387^{13}^	0.64722^{4}^	0.85777^{8}^	0.86556^{9}^	0.876^{11}^	1.15111^{14}^	0.60453^{2}^	0.67044^{5}^	1.31014^{15}^	0.54314^{1}^	0.60576^{3}^	0.81016^{7}^	0.69091^{6}^
	MSE($\overset{ˆ}{\alpha }$)	2.63006^{8}^	2.798^{11}^	3.01999^{13}^	2.20217^{6}^	2.76929^{10}^	2.91424^{12}^	2.74519^{9}^	3.28017^{14}^	0.98907^{2}^	1.82638^{3}^	3.48534^{15}^	0.06829^{1}^	2.01762^{5}^	2.6241^{7}^	1.95762^{4}^
	MSE($\overset{ˆ}{\beta }$)	1.24696^{8}^	1.33296^{12}^	1.52635^{13}^	0.69277^{4}^	1.29946^{10}^	1.24869^{9}^	1.30263^{11}^	2.00488^{14}^	0.59969^{2}^	0.77071^{6}^	2.65405^{15}^	0.50897^{1}^	0.63455^{3}^	1.13359^{7}^	0.75929^{5}^
	MRE($\overset{ˆ}{\alpha }$)	0.59315^{8}^	0.61293^{11}^	0.64706^{13}^	0.52528^{6}^	0.61112^{10}^	0.62727^{12}^	0.60873^{9}^	0.67878^{14}^	0.24982^{2}^	0.42516^{3}^	0.70107^{15}^	0.07051^{1}^	0.48981^{5}^	0.59162^{7}^	0.48633^{4}^
	MRE($\overset{ˆ}{\beta }$)	0.24741^{10}^	0.25466^{12}^	0.27539^{13}^	0.18492^{4}^	0.24508^{8}^	0.2473^{9}^	0.25028^{11}^	0.32889^{14}^	0.17272^{2}^	0.19155^{5}^	0.37432^{15}^	0.15518^{1}^	0.17307^{3}^	0.23147^{7}^	0.1974^{6}^
	*D*_*abs*_	0.04696^{2}^	0.04983^{7}^	0.05007^{8}^	0.04787^{3}^	0.04868^{6}^	0.05185^{9}^	0.05267^{11}^	0.05385^{12}^	0.04847^{5}^	0.04843^{4}^	0.05674^{13}^	0.04695^{1}^	0.06562^{15}^	0.05187^{10}^	0.0644^{14}^
	*D*_*max*_	0.07727^{5}^	0.08103^{7}^	0.08412^{9}^	0.07347^{3}^	0.07904^{6}^	0.08595^{11}^	0.08458^{10}^	0.09025^{12}^	0.07304^{2}^	0.0742^{4}^	0.09602^{14}^	0.06877^{1}^	0.09654^{15}^	0.08197^{8}^	0.09558^{13}^
	ASAE	0.03936^{5}^	0.03516^{7}^	0.0376^{9}^	0.03617^{3}^	0.03676^{6}^	0.03559^{11}^	0.03579^{10}^	0.04199^{12}^	0.04768^{2}^	0.04414^{4}^	0.05106^{14}^	0.04872^{1}^	0.05676^{15}^	0.04192^{8}^	0.05772^{13}^
	∑*Ranks*	66^{5}^	84^{10.5}^	101^{13}^	40^{3}^	73^{9}^	85^{12}^	84^{10.5}^	117^{14}^	30^{2}^	43^{4}^	130^{15}^	20^{1}^	68^{6.5}^	68^{6.5}^	71^{8}^
75	BIAS($\overset{ˆ}{\alpha }$)	1.18102^{7}^	1.21207^{9}^	1.38334^{14}^	1.14209^{6}^	1.31331^{12}^	1.23314^{10}^	1.24221^{11}^	1.3468^{13}^	0.60732^{2}^	1.05741^{3}^	1.53315^{15}^	0.09902^{1}^	1.10287^{4}^	1.20754^{8}^	1.12056^{5}^
	BIAS($\overset{ˆ}{\beta }$)	0.54193^{8}^	0.59765^{11}^	0.72688^{13}^	0.45766^{4}^	0.66199^{12}^	0.5688^{10}^	0.56092^{9}^	0.7431^{14}^	0.39776^{2}^	0.46816^{5}^	1.11287^{15}^	0.30135^{1}^	0.47793^{6}^	0.51925^{7}^	0.4514^{3}^
	MSE($\overset{ˆ}{\alpha }$)	1.78826^{7}^	1.85143^{8}^	2.2657^{14}^	1.74572^{5}^	2.09786^{12}^	1.93397^{11}^	1.92458^{10}^	2.18406^{13}^	0.89167^{2}^	1.63154^{3}^	2.78624^{15}^	0.01992^{1}^	1.7277^{4}^	1.85182^{9}^	1.76636^{6}^
	MSE($\overset{ˆ}{\beta }$)	0.47617^{8}^	0.58064^{11}^	0.82024^{13}^	0.35658^{4}^	0.70582^{12}^	0.53517^{9}^	0.53723^{10}^	0.93187^{14}^	0.25832^{2}^	0.36439^{5}^	1.99493^{15}^	0.15004^{1}^	0.38239^{6}^	0.46059^{7}^	0.32914^{3}^
	MRE($\overset{ˆ}{\alpha }$)	0.47241^{7}^	0.48483^{9}^	0.55334^{14}^	0.45684^{6}^	0.52532^{12}^	0.49326^{10}^	0.49689^{11}^	0.53872^{13}^	0.24293^{2}^	0.42296^{3}^	0.61326^{15}^	0.03961^{1}^	0.44115^{4}^	0.48302^{8}^	0.44822^{5}^
	MRE($\overset{ˆ}{\beta }$)	0.15484^{8}^	0.17076^{11}^	0.20768^{13}^	0.13076^{4}^	0.18914^{12}^	0.16251^{10}^	0.16026^{9}^	0.21231^{14}^	0.11365^{2}^	0.13376^{5}^	0.31796^{15}^	0.0861^{1}^	0.13655^{6}^	0.14836^{7}^	0.12897^{3}^
	*D*_*abs*_	0.02771^{2}^	0.02986^{5}^	0.03268^{11}^	0.02842^{3}^	0.03286^{12}^	0.02964^{4}^	0.02996^{6}^	0.03113^{8}^	0.03045^{7}^	0.03159^{10}^	0.03673^{13}^	0.02677^{1}^	0.03688^{15}^	0.03133^{9}^	0.03684^{14}^
	*D*_*max*_	0.04556^{3}^	0.04922^{6}^	0.0555^{12}^	0.04486^{2}^	0.05481^{11}^	0.04948^{8}^	0.04919^{5}^	0.05324^{10}^	0.04635^{4}^	0.04942^{7}^	0.06517^{15}^	0.03937^{1}^	0.05675^{14}^	0.05017^{9}^	0.05631^{13}^
	ASAE	0.01847^{3}^	0.01738^{6}^	0.01922^{12}^	0.01785^{2}^	0.01868^{11}^	0.0168^{8}^	0.01807^{5}^	0.02087^{10}^	0.0243^{4}^	0.02226^{7}^	0.02974^{15}^	0.02362^{1}^	0.02726^{14}^	0.02087^{9}^	0.0274^{13}^
	∑*Ranks*	55^{5}^	72^{7.5}^	111^{14}^	37^{3}^	101^{12}^	73^{9.5}^	75^{11}^	107^{13}^	35^{2}^	51^{4}^	133^{15}^	19^{1}^	72^{7.5}^	73^{9.5}^	66^{6}^
150	BIAS($\overset{ˆ}{\alpha }$)	1.00203^{6}^	1.07945^{12}^	1.08943^{13}^	0.95719^{4}^	1.04245^{10}^	1.05253^{11}^	1.02845^{8}^	1.09536^{14}^	0.6008^{2}^	0.99947^{5}^	1.3498^{15}^	0.07377^{1}^	1.00858^{7}^	1.03052^{9}^	0.95379^{3}^
	BIAS($\overset{ˆ}{\beta }$)	0.37797^{8}^	0.4242^{11}^	0.47964^{13}^	0.32614^{3}^	0.44657^{12}^	0.40077^{9}^	0.41034^{10}^	0.54851^{14}^	0.31215^{2}^	0.37051^{6}^	0.86818^{15}^	0.21551^{1}^	0.34015^{4}^	0.37316^{7}^	0.34625^{5}^
	MSE($\overset{ˆ}{\alpha }$)	1.35369^{4}^	1.49149^{12}^	1.51848^{13}^	1.33127^{3}^	1.40963^{8}^	1.45058^{10}^	1.3891^{5}^	1.54847^{14}^	0.86076^{2}^	1.39784^{6}^	2.22901^{15}^	0.01287^{1}^	1.48665^{11}^	1.40708^{7}^	1.41706^{9}^
	MSE($\overset{ˆ}{\beta }$)	0.23433^{8}^	0.29162^{11}^	0.39463^{13}^	0.17823^{3}^	0.33563^{12}^	0.27965^{9}^	0.28935^{10}^	0.49627^{14}^	0.1602^{2}^	0.23375^{7}^	1.25448^{15}^	0.07538^{1}^	0.18168^{4}^	0.22929^{6}^	0.18998^{5}^
	MRE($\overset{ˆ}{\alpha }$)	0.40081^{6}^	0.43178^{12}^	0.43577^{13}^	0.38287^{4}^	0.41698^{10}^	0.42101^{11}^	0.41138^{8}^	0.43814^{14}^	0.24032^{2}^	0.39979^{5}^	0.53992^{15}^	0.02951^{1}^	0.40343^{7}^	0.41221^{9}^	0.38152^{3}^
	MRE($\overset{ˆ}{\beta }$)	0.10799^{8}^	0.1212^{11}^	0.13704^{13}^	0.09318^{3}^	0.12759^{12}^	0.11451^{9}^	0.11724^{10}^	0.15672^{14}^	0.08918^{2}^	0.10586^{6}^	0.24805^{15}^	0.06157^{1}^	0.09719^{4}^	0.10662^{7}^	0.09893^{5}^
	*D*_*abs*_	0.01886^{1}^	0.02075^{6}^	0.02227^{10}^	0.01963^{3}^	0.02123^{8}^	0.02011^{5}^	0.01976^{4}^	0.02196^{9}^	0.02231^{11}^	0.02316^{12}^	0.02656^{13}^	0.01913^{2}^	0.02679^{15}^	0.02122^{7}^	0.02659^{14}^
	*D*_*max*_	0.031^{2}^	0.03436^{8}^	0.03726^{11}^	0.03113^{3}^	0.03537^{9}^	0.03342^{5}^	0.03251^{4}^	0.03728^{12}^	0.03428^{6}^	0.03659^{10}^	0.04781^{15}^	0.02822^{1}^	0.04129^{14}^	0.03429^{7}^	0.04099^{13}^
	ASAE	0.01154^{2}^	0.01123^{8}^	0.01211^{11}^	0.01113^{3}^	0.01203^{9}^	0.0108^{5}^	0.01133^{4}^	0.01381^{12}^	0.01555^{6}^	0.01407^{10}^	0.01989^{15}^	0.01521^{1}^	0.018^{14}^	0.01337^{7}^	0.01733^{13}^
	∑*Ranks*	48^{4}^	86^{11}^	106^{13}^	28^{2}^	87^{12}^	70^{8.5}^	63^{5}^	114^{14}^	41^{3}^	67^{6.5}^	133^{15}^	20^{1}^	80^{10}^	67^{6.5}^	70^{8.5}^
200	BIAS($\overset{ˆ}{\alpha }$)	0.94192^{4}^	0.97585^{12}^	0.97111^{11}^	0.95588^{7}^	0.95474^{6}^	0.96076^{9}^	1.00598^{13}^	1.0517^{14}^	0.59666^{2}^	0.9541^{5}^	1.28631^{15}^	0.06127^{1}^	0.97009^{10}^	0.95862^{8}^	0.90552^{3}^
	BIAS($\overset{ˆ}{\beta }$)	0.3216^{6}^	0.37146^{10}^	0.43395^{13}^	0.28723^{3}^	0.4136^{12}^	0.33624^{7}^	0.37232^{11}^	0.47717^{14}^	0.27174^{2}^	0.33971^{8}^	0.7601^{15}^	0.1876^{1}^	0.31032^{4}^	0.34007^{9}^	0.31329^{5}^
	MSE($\overset{ˆ}{\alpha }$)	1.20999^{4}^	1.23911^{6}^	1.25547^{7}^	1.31195^{11}^	1.20754^{3}^	1.21062^{5}^	1.33841^{12}^	1.43268^{14}^	0.81688^{2}^	1.27996^{9}^	2.04928^{15}^	0.00711^{1}^	1.38742^{13}^	1.26403^{8}^	1.30866^{10}^
	MSE($\overset{ˆ}{\beta }$)	0.17197^{6}^	0.21715^{10}^	0.30854^{13}^	0.13796^{3}^	0.27912^{12}^	0.1906^{9}^	0.22531^{11}^	0.35604^{14}^	0.12077^{2}^	0.181^{7}^	0.95621^{15}^	0.05636^{1}^	0.1554^{4}^	0.18886^{8}^	0.1556^{5}^
	MRE($\overset{ˆ}{\alpha }$)	0.37677^{4}^	0.39034^{12}^	0.38844^{11}^	0.38235^{7}^	0.3819^{6}^	0.38431^{9}^	0.40239^{13}^	0.42068^{14}^	0.23866^{2}^	0.38164^{5}^	0.51452^{15}^	0.02451^{1}^	0.38803^{10}^	0.38345^{8}^	0.36221^{3}^
	MRE($\overset{ˆ}{\beta }$)	0.09188^{6}^	0.10613^{10}^	0.12399^{13}^	0.08207^{3}^	0.11817^{12}^	0.09607^{7}^	0.10638^{11}^	0.13634^{14}^	0.07764^{2}^	0.09706^{8}^	0.21717^{15}^	0.0536^{1}^	0.08866^{4}^	0.09716^{9}^	0.08951^{5}^
	*D*_*abs*_	0.01654^{2}^	0.01735^{4}^	0.01861^{7}^	0.01633^{1}^	0.01925^{10}^	0.01766^{5}^	0.01818^{6}^	0.01955^{12}^	0.0191^{8}^	0.01923^{9}^	0.02341^{15}^	0.01664^{3}^	0.0223^{14}^	0.01946^{11}^	0.02214^{13}^
	*D*_*max*_	0.02699^{3}^	0.02867^{4}^	0.03135^{10}^	0.02612^{2}^	0.03202^{11}^	0.02894^{5}^	0.02988^{7}^	0.03295^{12}^	0.02943^{6}^	0.03078^{8}^	0.042^{15}^	0.02453^{1}^	0.03467^{14}^	0.03117^{9}^	0.03439^{13}^
	ASAE	0.00957^{3}^	0.00937^{4}^	0.01016^{10}^	0.00923^{2}^	0.01026^{11}^	0.009^{5}^	0.00942^{7}^	0.01138^{12}^	0.01305^{6}^	0.01209^{8}^	0.01677^{15}^	0.01253^{1}^	0.01467^{14}^	0.01131^{9}^	0.01446^{13}^
	∑*Ranks*	40^{4}^	71^{8}^	91^{13}^	39^{3}^	79^{10}^	57^{5}^	88^{12}^	117^{14}^	38^{2}^	69^{6}^	135^{15}^	21^{1}^	87^{11}^	78^{9}^	70^{7}^
250	BIAS($\overset{ˆ}{\alpha }$)	0.89915^{8}^	0.9218^{11}^	0.89471^{6}^	0.86958^{4}^	0.90135^{9}^	0.89781^{7}^	0.95519^{13}^	0.99548^{14}^	0.57787^{2}^	0.94947^{12}^	1.25247^{15}^	0.0575^{1}^	0.88649^{5}^	0.91617^{10}^	0.80993^{3}^
	BIAS($\overset{ˆ}{\beta }$)	0.30805^{7}^	0.31306^{10}^	0.37803^{13}^	0.25526^{3}^	0.37142^{12}^	0.30831^{8}^	0.33164^{11}^	0.41755^{14}^	0.25192^{2}^	0.31127^{9}^	0.70426^{15}^	0.17145^{1}^	0.27887^{4}^	0.30065^{6}^	0.28997^{5}^
	MSE($\overset{ˆ}{\alpha }$)	1.11189^{6}^	1.13696^{8}^	1.07875^{3}^	1.12449^{7}^	1.08775^{4}^	1.0898^{5}^	1.20944^{12}^	1.29749^{14}^	0.79412^{2}^	1.27945^{13}^	1.9262^{15}^	0.007^{1}^	1.19954^{11}^	1.15042^{10}^	1.14042^{9}^
	MSE($\overset{ˆ}{\beta }$)	0.15248^{7}^	0.15877^{9}^	0.23066^{13}^	0.10584^{3}^	0.21618^{12}^	0.16203^{10}^	0.17406^{11}^	0.29036^{14}^	0.10394^{2}^	0.15354^{8}^	0.82268^{15}^	0.0454^{1}^	0.12343^{4}^	0.14308^{6}^	0.13914^{5}^
	MRE($\overset{ˆ}{\alpha }$)	0.35966^{8}^	0.36872^{11}^	0.35788^{6}^	0.34783^{4}^	0.36054^{9}^	0.35912^{7}^	0.38208^{13}^	0.39819^{14}^	0.23115^{2}^	0.37979^{12}^	0.50099^{15}^	0.023^{1}^	0.3546^{5}^	0.36647^{10}^	0.32397^{3}^
	MRE($\overset{ˆ}{\beta }$)	0.08801^{7}^	0.08945^{10}^	0.10801^{13}^	0.07293^{3}^	0.10612^{12}^	0.08809^{8}^	0.09476^{11}^	0.1193^{14}^	0.07198^{2}^	0.08894^{9}^	0.20122^{15}^	0.04899^{1}^	0.07968^{4}^	0.0859^{6}^	0.08285^{5}^
	*D*_*abs*_	0.01437^{2}^	0.01471^{3}^	0.01676^{8}^	0.01431^{1}^	0.01685^{10}^	0.01523^{4}^	0.01599^{6}^	0.01675^{7}^	0.0172^{11}^	0.01792^{12}^	0.02058^{15}^	0.01529^{5}^	0.02^{14}^	0.01684^{9}^	0.01906^{13}^
	*D*_*max*_	0.02377^{3}^	0.02428^{4}^	0.02815^{9}^	0.02282^{2}^	0.02818^{10}^	0.02503^{5}^	0.02631^{6}^	0.02845^{11}^	0.0266^{7}^	0.02871^{12}^	0.03725^{15}^	0.02257^{1}^	0.03116^{14}^	0.02704^{8}^	0.02972^{13}^
	ASAE	0.00821^{3}^	0.0082^{4}^	0.00901^{9}^	0.00813^{2}^	0.0087^{10}^	0.00787^{5}^	0.00824^{6}^	0.00997^{11}^	0.01115^{7}^	0.01041^{12}^	0.01475^{15}^	0.01103^{1}^	0.01239^{14}^	0.00966^{8}^	0.01242^{13}^
	∑*Ranks*	52^{4}^	69^{6}^	78^{10}^	29^{2}^	84^{11}^	55^{5}^	88^{12}^	111^{14}^	42^{3}^	97^{13}^	135^{15}^	23^{1}^	74^{9}^	73^{8}^	70^{7}^
400	BIAS($\overset{ˆ}{\alpha }$)	0.81073^{8}^	0.85487^{12}^	0.79208^{5}^	0.82732^{10}^	0.80311^{6}^	0.82421^{9}^	0.80676^{7}^	0.89825^{14}^	0.56718^{2}^	0.85575^{13}^	1.15114^{15}^	0.04449^{1}^	0.76167^{4}^	0.84546^{11}^	0.69598^{3}^
	BIAS($\overset{ˆ}{\beta }$)	0.24106^{6}^	0.27021^{11}^	0.2979^{12}^	0.21989^{3}^	0.30105^{13}^	0.24154^{7}^	0.25477^{9}^	0.34098^{14}^	0.21422^{2}^	0.24602^{8}^	0.58883^{15}^	0.13318^{1}^	0.2399^{5}^	0.25736^{10}^	0.23072^{4}^
	MSE($\overset{ˆ}{\alpha }$)	0.94131^{7}^	1.0301^{11}^	0.88787^{4}^	1.05768^{13}^	0.88055^{3}^	0.96021^{8}^	0.925^{5}^	1.06866^{14}^	0.7199^{2}^	1.03776^{12}^	1.6502^{15}^	0.00387^{1}^	1.01982^{10}^	1.00117^{9}^	0.93436^{6}^
	MSE($\overset{ˆ}{\beta }$)	0.08982^{5}^	0.11445^{11}^	0.14286^{12}^	0.07489^{2}^	0.14464^{13}^	0.09543^{8}^	0.10274^{9}^	0.19187^{14}^	0.07951^{3}^	0.09384^{6}^	0.56292^{15}^	0.02856^{1}^	0.09473^{7}^	0.1029^{10}^	0.08821^{4}^
	MRE($\overset{ˆ}{\alpha }$)	0.32429^{8}^	0.34195^{12}^	0.31683^{5}^	0.33093^{10}^	0.32124^{6}^	0.32968^{9}^	0.3227^{7}^	0.3593^{14}^	0.22687^{2}^	0.3423^{13}^	0.46046^{15}^	0.0178^{1}^	0.30467^{4}^	0.33818^{11}^	0.27839^{3}^
	MRE($\overset{ˆ}{\beta }$)	0.06887^{6}^	0.0772^{11}^	0.08511^{12}^	0.06282^{3}^	0.08601^{13}^	0.06901^{7}^	0.07279^{9}^	0.09742^{14}^	0.06121^{2}^	0.07029^{8}^	0.16824^{15}^	0.03805^{1}^	0.06854^{5}^	0.07353^{10}^	0.06592^{4}^
	*D*_*abs*_	0.01086^{1}^	0.01227^{5}^	0.0131^{8}^	0.01184^{3}^	0.01256^{7}^	0.01233^{6}^	0.01193^{4}^	0.01341^{9}^	0.01375^{11}^	0.01371^{10}^	0.01668^{15}^	0.01182^{2}^	0.01568^{14}^	0.01389^{12}^	0.01532^{13}^
	*D*_*max*_	0.01793^{2}^	0.02022^{6}^	0.02179^{9}^	0.01901^{3}^	0.02108^{7}^	0.02014^{5}^	0.01963^{4}^	0.02274^{12}^	0.02142^{8}^	0.02198^{10}^	0.03028^{15}^	0.01744^{1}^	0.02456^{14}^	0.0224^{11}^	0.02391^{13}^
	ASAE	0.00613^{2}^	0.00602^{6}^	0.00667^{9}^	0.00615^{3}^	0.00656^{7}^	0.00581^{5}^	0.00614^{4}^	0.00731^{12}^	0.0084^{8}^	0.00779^{10}^	0.01126^{15}^	0.00825^{1}^	0.00937^{14}^	0.00726^{11}^	0.00928^{13}^
	∑*Ranks*	46^{3}^	81^{11}^	74^{8.5}^	52^{4}^	74^{8.5}^	60^{6}^	58^{5}^	114^{14}^	44^{2}^	90^{12}^	135^{15}^	20^{1}^	77^{10}^	92^{13}^	63^{7}^

**Table 9:** Partial and overall ranks of all the methods of estimation of proposed distribution by various values of model parameters.

Parameter	*n*	MLE	ADE	CVME	MPSE	OLSE	RTADE	WLSE	LTADE	MSADE	MSALDE	ADSOE	KE	MSSD	MSSLD	MSLND
*α* = 1.5, *β* = 2.5	25	12.0	9.0	14.0	2.0	3.0	6.5	8.0	13.0	6.5	5.0	15.0	1.0	11.0	4.0	10.0
	75	5.0	12.0	14.0	4.0	10.0	11.0	6.0	13.0	3.0	7.0	15.0	1.0	8.0	2.0	9.0
	150	4.0	2.0	11.0	7.0	12.0	5.5	3.0	10.0	5.5	9.0	15.0	1.0	13.0	14.0	8.0
	200	1.0	3.0	12.0	5.0	6.0	7.0	4.0	13.0	8.0	14.0	15.0	2.0	10.0	9.0	11.0
	250	1.0	7.0	13.0	4.0	5.0	9.0	3.0	12.0	6.0	11.0	15.0	2.0	8.0	10.0	14.0
	400	4.0	3.0	11.0	5.0	10.0	6.0	2.0	12.0	7.0	13.5	15.0	1.0	9.0	8.0	13.5

*α* = 0.6, *β* = 0.9	25	13.0	10.0	5.5	3.5	12.0	9.0	7.0	8.0	5.5	11.0	15.0	1.0	14.0	2.0	3.5
	75	2.0	6.5	8.0	3.0	13.0	14.0	5.0	9.0	10.0	6.5	15.0	1.0	11.0	4.0	12.0
	150	6.0	5.0	9.0	1.0	14.0	11.5	4.0	13.0	7.5	11.5	15.0	2.0	10.0	3.0	7.5
	200	5.0	3.0	11.0	2.0	6.5	10.0	8.0	13.0	6.5	9.0	15.0	1.0	14.0	4.0	12.0
	250	7.0	8.0	6.0	2.0	4.5	11.0	9.0	12.0	3.0	10.0	13.0	1.0	15.0	4.5	14.0
	400	2.0	7.0	3.0	4.0	10.0	11.0	6.0	5.0	8.0	12.0	13.5	1.0	13.5	9.0	15.0

*α* = 1.0, *β* = 1.0	25	9.0	10.0	4.0	1.0	3.0	7.0	8.0	12.0	11.0	13.0	15.0	2.0	6.0	5.0	14.0
	75	9.0	12.0	2.0	6.0	10.0	7.0	4.5	14.0	4.5	8.0	15.0	1.0	13.0	3.0	11.0
	150	8.5	4.0	2.0	7.0	11.0	3.0	5.0	14.0	6.0	12.5	15.0	1.0	12.5	10.0	8.5
	200	10.0	2.0	5.5	7.0	3.0	4.0	5.5	12.0	8.0	9.0	15.0	1.0	13.0	11.0	14.0
	250	2.0	8.0	4.0	10.0	7.0	11.0	3.0	5.0	9.0	12.0	15.0	1.0	13.0	6.0	14.0
	400	3.0	4.0	6.0	8.0	9.0	5.0	7.0	2.0	11.0	14.0	15.0	1.0	13.0	10.0	12.0

*α* = 0.75, *β* = 1.5	25	2.0	5.0	14.0	3.0	8.0	11.0	12.0	13.0	9.0	6.0	15.0	1.0	7.0	4.0	10.0
	75	3.0	8.0	14.0	2.0	7.0	10.0	5.5	13.0	9.0	4.0	15.0	1.0	11.0	5.5	12.0
	150	3.0	6.0	11.0	2.0	5.0	8.0	7.0	14.0	9.5	4.0	15.0	1.0	12.0	9.5	13.0
	200	2.0	10.0	8.0	5.0	4.0	6.0	3.0	11.0	7.0	9.0	15.0	1.0	13.0	12.0	14.0
	250	3.0	9.0	6.0	7.5	2.0	5.0	7.5	10.0	4.0	11.0	15.0	1.0	13.0	12.0	14.0
	400	1.0	5.0	9.0	7.5	4.0	7.5	2.0	11.0	10.0	12.0	14.0	3.0	13.0	6.0	15.0

*α* = 2.5, *β* = 3.5	25	5.0	10.5	13.0	3.0	9.0	12.0	10.5	14.0	2.0	4.0	15.0	1.0	6.5	6.5	8.0
	75	5.0	7.5	14.0	3.0	12.0	9.5	11.0	13.0	2.0	4.0	15.0	1.0	7.5	9.5	6.0
	150	4.0	11.0	13.0	2.0	12.0	8.5	5.0	14.0	3.0	6.5	15.0	1.0	10.0	6.5	8.5
	200	4.0	8.0	13.0	3.0	10.0	5.0	12.0	14.0	2.0	6.0	15.0	1.0	11.0	9.0	7.0
	250	4.0	6.0	10.0	2.0	11.0	5.0	12.0	14.0	3.0	13.0	15.0	1.0	9.0	8.0	7.0
	400	3.0	11.0	8.5	4.0	8.5	6.0	5.0	14.0	2.0	12.0	15.0	1.0	10.0	13.0	7.0

∑ Ranks		142.5	212.5	274.5	125.5	241.5	242.0	190.5	347.0	188.5	279.5	445.5	36.0	330.0	220.0	324.5
Overall Rank		3	6	10	2	8	9	5	14	4	11	15	1	13	7	12

## 6 Illustrations with real data

In this section, we have considered three real datasets to assess the proposed model's capability of goodness-of-fit and adequacy of the model. These are as follows:

• Data set I by [16]: 1.43, 0.11, 0.71, 0.77, 2.63, 1.49, 3.46, 2.46, 0.59, 0.74, 1.23, 0.94, 4.36, 0.40, 1.74, 4.73, 2.23, 0.45, 0.70, 1.06, 1.46, 0.30, 1.82, 2.37, 0.63, 1.23, 1.24, 1.97, 1.86, 1.17.
• Data set II by [16]: 0.008, 0.017, 0.058, 0.061, 0.084, 0.090, 0.134, 0.238, 0.245, 0.353, 0.374, 0.480, 0.495, 0.535, 0.564, 0.681, 0.686, 0.688, 0.921, 0.959, 1.022, 1.092, 1.260, 1.284, 1.295, 1.373, 1.395, 1.414, 1.760, 1.858, 1.892, 1.921, 1.926, 1.933, 2.135, 2.169, 2.301, 2.320, 2.405, 2.506, 2.598, 2.808, 2.971, 3.087, 3.492, 3.669, 3.926, 4.446, 5.119, 8.596.
• Data set III by [13]: 17.88, 28.92, 33.0, 41.52, 42.12, 45.6, 48.8, 51.84, 51.96, 54.12, 55.56, 67.8, 68.44, 68.88, 84.12, 93.12, 98.64, 105.12, 105.84, 105.84, 127.92, 128.04, 173.4.

### 6.1 Model selection and goodness of fit test

In this subsection, we analyzed data sets I, II, and III, utilizing several established goodness-of-fit statistics and model selection criteria. The assessment of the fitted models encompassed a range of metrics, including the log-likelihood value (-2logL), Akaike information criterion (AIC), Hannan-Quinn information criterion (HQIC), Anderson-Darling (AD) statistic, Kolmogorov-Smirnov (KS) test, and Cramer-von Mises (CVM) test. The mathematical expressions can be found in work by Sapkota and Kumar (2023) [21] for these statistics computations.

All the necessary calculations and graphical representations were executed using the R software (R core team, 2023) [20]. To gauge the fitting performance of the NTPQED, we compared it against several other models, namely

1. Extension of exponential (EE) distribution (Nadarajah and Haghighi, 2011) [17]
2. XLindley (XL) distribution (Nawel et al., 2023) [18]
3. Modified XLindley (MXL) distribution (Gemeay et al., 2023) [10]
4. Burr type X (BurrX) distribution (Burr, 1942) [6]

The MLEs for all the examined distributions are provided in Table 10, Table 11, Table 12 for the respective datasets. These tables also include the estimate's corresponding standard errors (SE).

**Table 10:** Estimated parameters and SE for dataset-I.

Model	parameter	SE	parameter	SE
NTPQED(*α*,*β*)	0.0235	0.2518	1.5761	0.1941
BurrX(*λ*,*θ*)	0.6284	0.1412	0.4467	0.0618
Exp(*λ*)	0.6482	0.1183	–	–
XL(*β*)	0.9976	0.1585	–	–
MXL(*δ*)	1.1886	0.1919	–	–
EE(*α*,*θ*)	4.4381	6.9134	0.0979	0.1746

**Table 11:** Estimated parameters and SE for dataset-II.

Model	parameter	SE	parameter	SE
NTPQED(*α*,*β*)	3.5039	3.1887	1.0390	0.1789
BurrX(*λ*,*θ*)	0.3442	0.0553	0.2903	0.0380
Exp(*λ*)	0.5978	0.0845	–	–
XL(*β*)	0.8868	0.1103	–	–
MXL(*δ*)	0.2141	0.0286	–	–
EE(*α*,*θ*)	1.1800	0.4537	0.4634	0.2761

**Table 12:** Estimated parameters and SE for dataset-III.

Model	parameter	SE	parameter	SE
NTPQED(*α*,*β*)	0.0186	221.4690	0.0406	0.0000
BurrX(*λ*,*θ*)	1.1794	0.3359	0.0127	0.0017
Exp(*λ*)	0.0135	0.0028	–	–
XL(*β*)	0.0135	0.0028	–	–
MXL(*δ*)	87.5162	0.0028	–	–
EE(*α*,*θ*)	33.0173	29.5086	3.00E-04	3.00E-04

**Figure 8:**
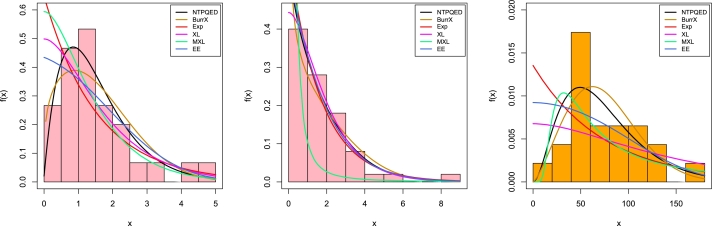
Fitted PDF of all models under comparison for data sets I, II, and III, respectively.

**Figure 9:**
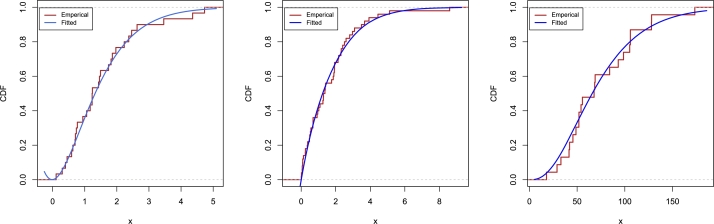
Fitted CDF of all models under comparison for data sets I, II, and III, respectively.

**Figure 10:**
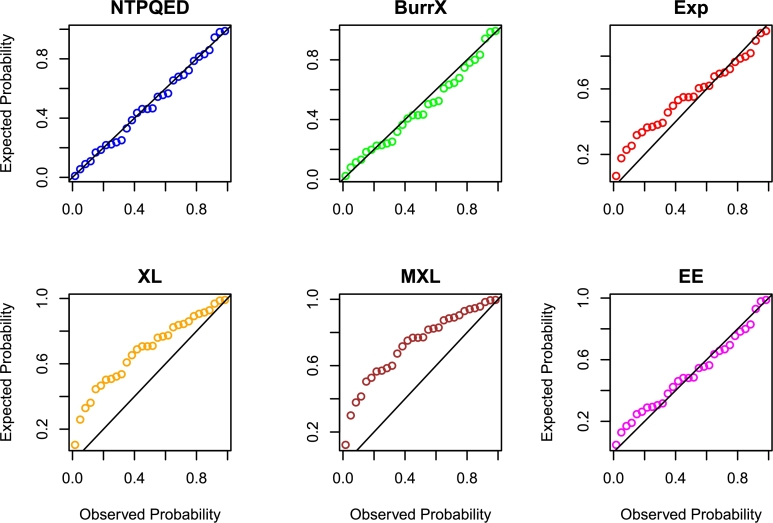
P-P plots of all models under comparison for data set I.

**Figure 11:**
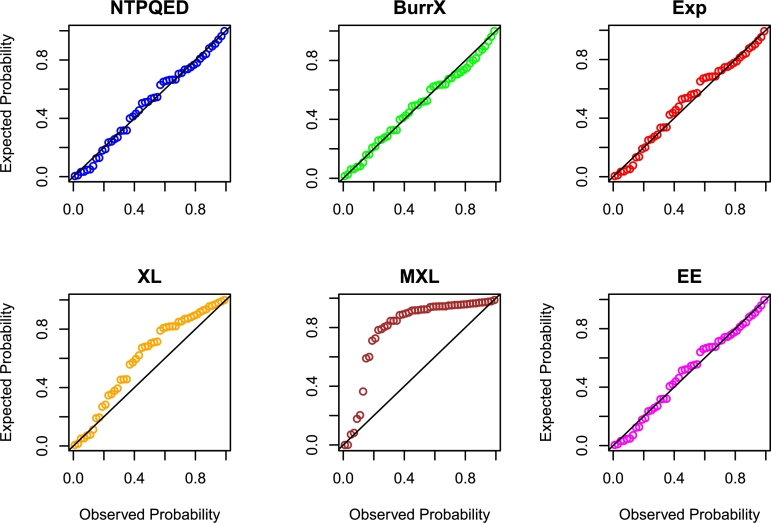
P-P plots of all models under comparison for data set II.

**Figure 12:**
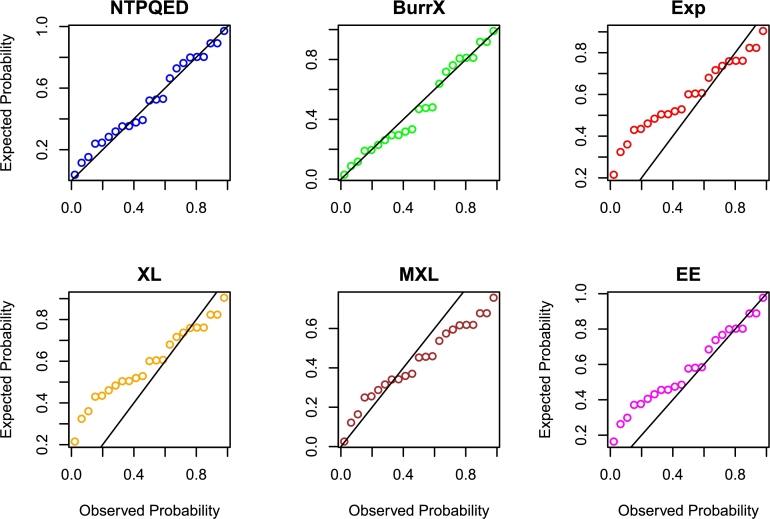
P-P plots of all models under comparison for data set III.

Moreover, Table 13, Table 14, Table 15 demonstrate the criteria used for model selection, including log-likelihood, HQIC, and AIC, as well as the goodness of fit statistics like KS, AD, and CVME with associated p-values for all three datasets. Our analysis reveals that among the BurrX, Exp, XL, MXL, and EE distributions, the NTPQED exhibits the lowest statistics and simultaneously the highest corresponding p-values. Further, our model is far better for data sets I and III, but BurrX and NTPQED provide similar fits for data sets II. This means BurrX and NTPQED are performing quite similarly for heavy-tailed right-skewed data. These results imply that the NTPQED model offers greater flexibility and a more favorable fit. Additionally, graphical representations of the fitted models are presented in Figure 8, Figure 9 for all three datasets, providing visual support for our conclusion that the NTPQED model surpasses the performance of the other considered models. Further, we have also displayed the probability-probability (P-P) plots of all the models under comparison for all three data sets. As illustrated, our model demonstrates a closer alignment to the diagonal line, indicating a better fit to the observed data than the competing models. This suggests that our model more accurately captures the underlying distribution of the data (see Figure 10, Figure 11, Figure 12).

**Table 13:** Goodness of fit and model selection criterion for dataset-I.

Model	-2logL	AIC	HQIC	KS	p(KS)	CVM	p(CVM)	AD	p(AD)
NTPQED	79.4691	83.4691	84.3656	0.0828	0.9863	0.0219	0.9956	0.1727	0.9962
Burr	81.1461	85.1461	86.0426	0.1091	0.8678	0.064	0.7926	0.4043	0.8433
Exp	86.0108	88.0108	88.4590	0.1845	0.2590	0.2324	0.2129	1.3298	0.2228
XL	83.1250	85.1250	85.5733	0.3115	0.0059	1.1757	8.00E-04	5.8462	0.0012
MXL	93.8849	95.8849	96.3332	0.1460	0.5446	0.1921	0.2842	1.2572	0.2465
EE	82.3079	86.3079	87.2045	0.1132	0.837	0.0709	0.7496	0.5163	0.7289

**Table 14:** Goodness of fit and model selection criterion for dataset-II.

Model	-2logL	AIC	HQIC	KS	p(KS)	CVM	p(CVM)	AD	p(AD)
NTPQED	150.9367	154.9367	156.3929	0.0704	0.9503	0.0414	0.9276	0.3877	0.8602
BurrX	151.1598	155.1598	156.6160	0.0699	0.9533	0.0384	0.9428	0.2892	0.9458
Exp	151.4547	153.4547	154.1828	0.0908	0.7704	0.0683	0.7640	0.4747	0.7720
XL	151.8104	153.8104	154.5385	0.2329	0.0072	0.8427	0.0055	4.5730	0.0046
MXL	282.8590	284.8590	285.5871	0.5630	0.0000	5.9703	0.0000	37.5083	0.0000
EE	151.2550	155.2550	156.7112	0.0808	0.8738	0.0519	0.8666	0.4435	0.8040

**Table 15:** Goodness of fit and model selection criterion for dataset-III.

Model	-2logL	AIC	HQIC	KS	p(KS)	CVM	p(CVM)	AD	p(AD)
NTPQED	228.3677	232.3677	232.9388	0.109	0.9476	0.0435	0.9185	0.2881	0.9464
BurrX	228.218	232.218	232.7891	0.1447	0.7217	0.0626	0.8028	0.3331	0.9105
Exp	243.8918	245.8918	246.1773	0.2996	0.0322	0.5126	0.0361	2.7274	0.0382
XL	245.2743	247.2743	247.5599	0.2996	0.0322	0.5126	0.0361	2.7274	0.0382
MXL	241.077	243.077	243.3626	0.2791	0.0555	0.3676	0.0874	2.0476	0.087
EE	235.359	239.359	239.9301	0.2408	0.1388	0.2805	0.1534	1.5758	0.1596

## 7 Conclusion

This research work has focused on exploring a novel two-parameter quadratic exponential distribution. The study delves into the fundamental statistical characteristics of this recently introduced distribution, such as its asymptotic behavior, moments, related measures, order statistics, and entropies. A brief overview of fuzzy reliability has been presented, including numerical values of fuzzy reliability, value at risk, mean excess function, limited expected value function, tail value at risk, and tail variance. Based on our analysis, a comprehensive simulation study was conducted to evaluate fifteen parameter estimation methods, with Kolmogorov estimation emerging as the most efficient method. Three sets of real-world data were utilized to demonstrate the practical utility of the proposed distribution. The performance of the suggested distribution model was assessed using various model selection criteria and goodness-of-fit test statistics. Empirical findings from these evaluations provide substantial evidence that the proposed model outperforms other existing models.

## CRediT authorship contribution statement

**Fatma Zohra Bousseba:** Writing – review & editing, Supervision, Methodology, Formal analysis, Conceptualization. **Halim Zeghdoudi:** Writing – review & editing, Validation, Resources, Methodology, Data curation, Conceptualization. **Laxmi Prasad Sapkota:** Writing – original draft, Validation, Methodology, Data curation, Conceptualization. **Yusra A. Tashkandy:** Validation, Software, Methodology, Formal analysis, Conceptualization. **M.E. Bakr:** Writing – review & editing, Writing – original draft, Software, Investigation, Formal analysis, Conceptualization. **Anoop Kumar:** Supervision, Software, Methodology. **Ahmed M. Gemeay:** Writing – review & editing, Supervision, Software, Resources, Project administration, Formal analysis, Data curation, Conceptualization.

## Declaration of Competing Interest

The authors declare that they have no known competing financial interests or personal relationships that could have appeared to influence the work reported in this paper.

## Data Availability

The data that supports the findings of this study are available within the article.

## References

[1] Aguilar G.A., Moala F.A., Cordeiro G.M. (2019). Zero-truncated poisson exponentiated gamma distribution: application and estimation methods. J. Stat. Theory Pract..

[2] Almetwally E.M., Meraou M.A. (2022). Application of environmental data with new extension of nadarajah-haghighi distribution. Comput. J. Math. Stat. Sci..

[3] Anderson T.W., Darling D.A. (1952). Asymptotic theory of certain “goodness of fit” criteria based on stochastic processes. Ann. Math. Stat..

[4] Beghriche A., Zeghdoudi H., Raman V., Chouia S. (2022). New polynomial exponential distribution: properties and applications. Stat. Trans. New Ser..

[5] Belili M.C., Alshangiti A.M., Gemeay A.M., Zeghdoudi H., Karakaya K., Bakr M.E., Balogun O.S., Atchadé M.N., Hussam E. (2023). Two-parameter family of distributions: properties, estimation, and applications. AIP Adv..

[6] Burr I.W. (1942). Cumulative frequency functions. Ann. Math. Stat..

[7] Chen G., Pham T.T. (2001). Introduction to fuzzy sets, fuzzy logic, and fuzzy control systems. Appl. Mech. Rev..

[8] Choi K., Bulgren W.G. (1968). An estimation procedure for mixtures of distributions. J. R. Stat. Soc., Ser. B, Methodol..

[9] Eghwerido J.T., Ogbo J.O., Omotoye A.E. (2021). The Marshall-Olkin Gompertz distribution: properties and applications. Statistica.

[10] Gemeay A.M., Beghriche A., Sapkota L.P., Zeghdoudi H., Makumi N., Bakr M., Balogun O.S. (2023). Modified xlindley distribution: properties, estimation, and applications. AIP Adv..

[11] Gemeay A.M., Halim Z., Abd El-Raouf M.M., Hussam E., Abdulrahman A.T., Mashaqbah N.K., Alshammari N., Makumi N. (2023). General two-parameter distribution: statistical properties, estimation, and application on covid-19. PLoS ONE.

[12] Kao J.H.K. (1958). Computer methods for estimating Weibull parameters in reliability studies. IRE Trans. Reliab. Qual. Control.

[13] Lawless J.F. (2011).

[14] Meriem B., Gemeay A.M., Almetwally E.M., Halim Z., Alshawarbeh E., Abdulrahman A.T., El-Raouf M.M.A., Hussam E. (2022). The power xlindley distribution: statistical inference, fuzzy reliability, and covid-19 application. J. Funct. Spaces.

[15] Muhammed H.Z., Almetwally E.M. (2024). Bayesian and non-bayesian estimation for the shape parameters of new versions of bivariate inverse Weibull distribution based on progressive type ii censoring. Comput. J. Math. Stat. Sci..

[16] Murthy D.N.P., Xie M., Jiang R. (2004).

[17] Nadarajah S., Haghighi F. (2011). An extension of the exponential distribution. Statistics.

[18] Nawel K., Gemeay A.M., Zeghdoudi H., Karakaya K., Alshangiti A.M., Bakr M., Balogun O.S., Muse A.H., Hussam E. (2023). Modeling voltage real data set by a new version of Lindley distribution. IEEE Access.

[19] Nedjar S., Zeghdoudi H. (2016). On gamma Lindley distribution: properties and simulations. J. Comput. Appl. Math..

[20] R Core Team (2023).

[21] Sapkota L.P., Kumar V. (2023). Chen exponential distribution with applications to engineering data. Int. J. Stat. Reliab. Eng..

[22] Swain J.J., Venkatraman S., Wilson J.R. (1988). Least-squares estimation of distribution functions in Johnson's translation system. J. Stat. Comput. Simul..

[23] Torabi H. (2008). A general method for estimating and hypotheses testing using spacings. J. Stat. Theory Appl..

